# Bis(2,2,2 trifluoroethyl) Phosphonate as a Convenient Precursor for the Synthesis of H-Phosphonates

**DOI:** 10.3390/molecules29112432

**Published:** 2024-05-21

**Authors:** Jean-Marie Pohl, Fabian Stöhr, Tim Kramer, Jonathan Becker, Richard Göttlich

**Affiliations:** 1 Institute for Organic Chemistry, Justus-Liebig-Universität Giessen, Heinrich-Buff-Ring 17, 35392 Giessen, Germany; 2 Institute for Inorganic and Analytical Chemistry, Justus-Liebig-Universität Giessen, Heinrich-Buff-Ring 17, 35392 Giessen, Germany

**Keywords:** H-phosphonate, microwave, dialkyl phosphite, dioxaphospholane, transesterification

## Abstract

A microwave-assisted synthesis of dialkyl and cyclic H-phosphonates via bis(2,2,2 trifluoroethyl) phosphonate (BTFEP) is described. This method enables the synthesis of various cyclic H-phosphonates and hetero-substituted dialkyl H-phosphonates by simple alcoholysis under non-inert and additive-free conditions. Short reaction times and the requirement for only stoichiometric amounts of alcohol render this method attractive for synthetic applications.

## 1. Introduction

Organophosphorus chemistry is a wide and well-established field in modern organic chemistry. One group of particular interest is that of H-phosphonates, the mono- and dialkyl esters of phosphonic acid. Their versatility is based on their unique chemical properties, merging the characteristics of P(III) phosphites and P(V) phosphates [1]. Based on their tautomerism between the P(III) phosphite form and the P(V) H-phosphonate form, they can either react as nucleophiles or as electrophiles [2,3]. Their broad use spans from building blocks for the formation of compounds, such as aminophosphonates [4], bisphosphonates [5], phosphates [6], nucleotides [7], to their application as catalysts [8,9] and ligands [10]. The synthesis of H-phosphonates is typically accomplished by direct substitution from phosphorous trichloride, either in a tert-butanolysis reaction, with excess alcohol or in the presence of a base. In recent years, considerable efforts were undertaken to develop alternative methods for the synthesis of this useful group of compounds [11,12]. The major alternative is based on the transesterification reaction, which in the past was utilized for the synthesis of hetero-substituted [13] and cyclic H-phosphonates [14]. A promising starting material for this type of reaction is bis(2,2,2-trifluoroethyl)phosphonate (BTFEP), which was first employed by Gibbs et al. in a reaction for the synthesis of mono-substituted H-phosphonates, utilizing transesterification via heating, to hetero-substituted H-phosphonates with subsequent saponification [15]. Similarly, good results were achieved with diphenyl H-phosphonate in a base-catalyzed reaction at room temperature for the synthesis of mono-substituted H-phosphonates [16]. This approach requires an excess of base for activation, utilizing pyridine as the solvent. Recently, BTFEP was utilized in the zinc-catalyzed synthesis of hetero-substituted H-phosphonates [17]. In previous studies by Keglevich et al., the microwave-assisted alcoholysis of phosphonates showed a promising acceleration in reaction speed in comparison to thermal heating, thereby making the addition of additives superfluous [18,19]. Building upon both strategies, we investigate herein the transesterification of BTFEP under microwave irradiation for the synthesis of H-phosphonates.

## 2. Results and Discussion

### 2.1. General Considerations

**Scheme 1:**
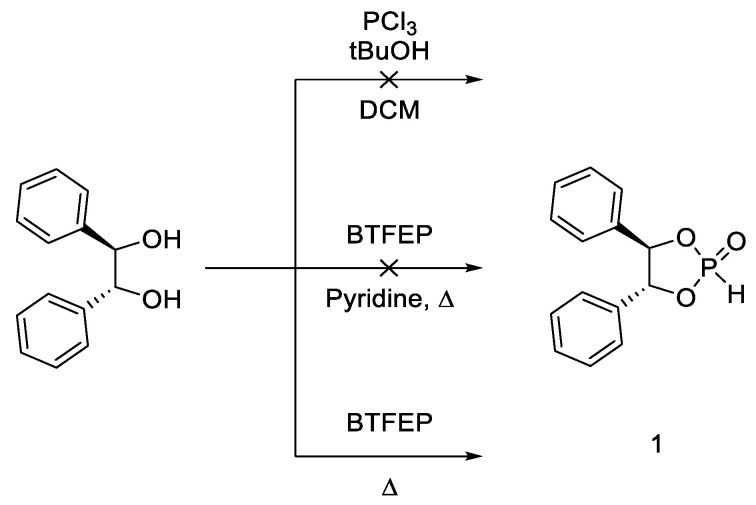
Approaches for the synthesis of H-phosphonate **1**.

One advantage of BTFEP is that it is easier to handle and less toxic than phosphorous halides [20]. BTFEP is stable under ambient conditions without any degradation of quality and does not generate noxious gases. While other H-phosphonates like diphenyl H-phosphonate show similar reactivity and an even higher stability under ambient conditions, one of the main benefits of BTFEP compared to diphenyl phosphonate is that 2,2,2-trifluoroethanol (TFE) is volatile and can therefore easily be removed in vacuo or by heating, even in the presence of phosphonates with low molecular weight. During our investigations, we were looking for an efficient preparation of chiral H-phosphonates, with our focus directed toward H-phosphonate **1** (Scheme 1).

The synthesis of this compound in a reaction with PCl_3_ as the reagent was previously described [21], but unfortunately, in our experiment, this method was not fully reproducible. The base-catalyzed reaction of BTFEP with hydrobenzoin to form **1** was attempted in the literature, but the authors described solely polymerization [22]. We took this generation of polymers as a sign of overreaction and therefore decided to omit the addition of base. This led to the formation of the desired product (Scheme 1). Yet, the uncatalyzed thermal transesterification reaction necessitated prolonged reaction times of 2 d under heating in THF, 1,4-dioxane or toluene. Under these conditions, BTFEP starts to decompose, thereby decreasing the yield considerably and hampering reproducibility.

### 2.2. Synthesis of Cyclic H-Phosphonates

Considering the results by Keglevich et al., we decided to attempt a microwave-assisted reaction to shorten the reaction time and thereby suppress decomposition of BTFEP.

**Scheme 2:**
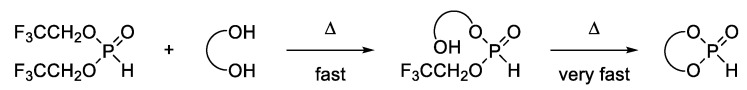
General reaction scheme for the synthesis of cyclic H-phosphonates.

Although this is a two-step reaction (Scheme 2), the second step of the transesterification proceeds at least at an equal or even faster rate in comparison to the first step due to a chelating effect. Therefore, in our experiment, the intermediate was not detectable. This exemplified that TFE is a suitable leaving group for this reaction.

**Scheme 3:**
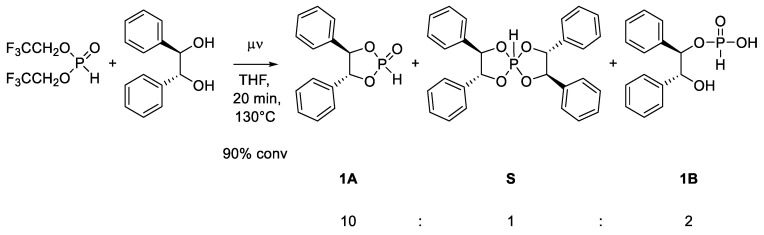
Reaction of hydrobenzoin with BTFEP. Ratios estimated via integration of all signals in ^31^P-NMR (accurate within ~10%) [24].

**Scheme 4:**
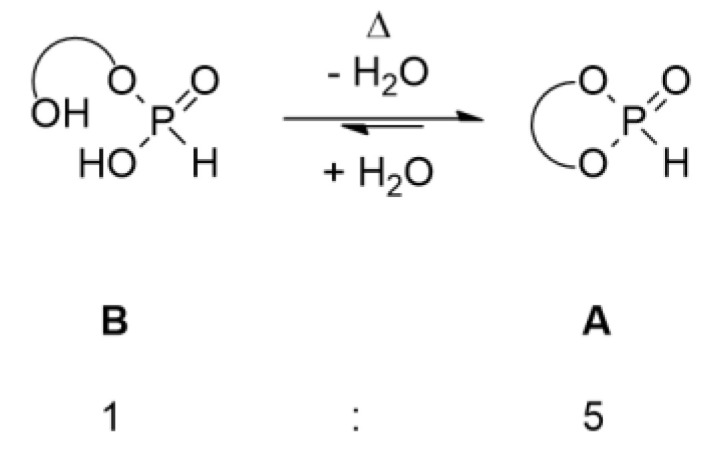
Proposed equilibrium of cyclic H-phosphonates.

However, we obtained products of hydrolysis and overreaction (Scheme 3). The reaction with hydrobenzoin shows, in addition to generation of the desired dioxaphospholane **1A**, the formation of spirophosphorane **S**, as well as the open-chain hydrolyzed product **1B**. The formation of spirophosphorane suggests that a second substitution takes place, which occurs after the formation of dioxaphospholane [23]. This unwanted side reaction was effectively suppressed by increasing the ratio of BTFEP, as well as working under dilute conditions (Appendix A). Thus, we found that the optimized conditions of 1.4 equiv. BTFEP and 0.5 mol/L diol completely suppressed the formation of spirophosphorane. We also noted that phosphonate **1** showed quick hydrolysis, which at a ratio of roughly 5:1 decelerated and then only slowly continued. This observation indicates that the ring opening/closing is in equilibrium (Scheme 4).

**Figure 1:**
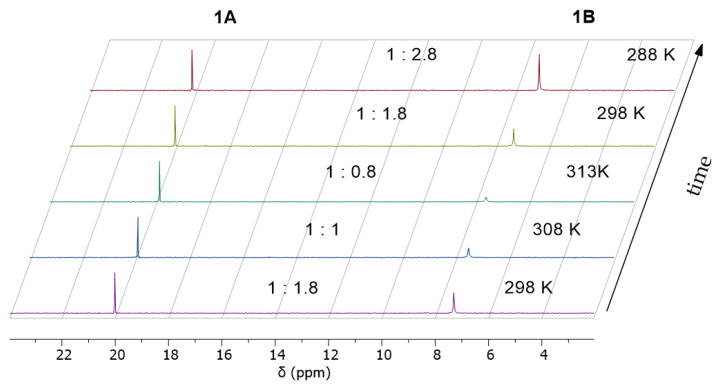
Temperature dependence of the ratio between cyclic form (**1A**) and open-chain form (**1B**) of **1**. A sample with significant hydrolysis was chosen.

Temperature-dependent ^31^P-NMR measurements show that a variation in temperature leads to a reversible shift in the ratio between cyclic form **1A** and open-chain form **1B** of the H-phosphonate, with **1A** being preferred at elevated temperatures (Figure 1).

It appears that the ring closing/opening is in a strict equilibrium, which can be completely cycled by variation in temperature. Such an equilibrium was postulated in the past for the glycol phosphonate [25]. Similarly, it was previously described that the dimethylamine salts of acyclic phosphonates can be reclosed to the dioxaphosphorinane-based H-phosphonate by heating [26].

During our attempts to achieve complete ring closure of the purified H-phosphonate with basic desiccants, we noticed the formation of an insoluble “polymeric” solid and complete decomposition of **1**. All other attempts, e.g., heating, removal by vacuum or desiccants, either led to incomplete removal of water or decomposition. Two noticeable impurities which appeared during these attempts (two signals in ^31^P-NMR around 31 ppm) correspond to C-phosphonates and suggest some form of elimination during the reaction. This would explain the apparent generation of water, even while precautions to work under anhydrous conditions were taken.

**Figure 2:**
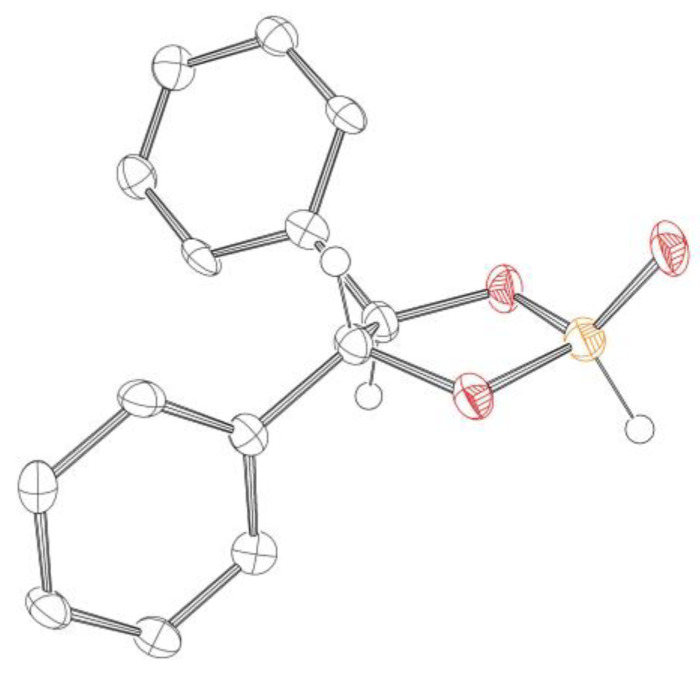
Molecular structure obtained by SC-XRD of phosphonate **1**. Hydrogens are omitted for clarity (except for P−H and aliphatic C−H); anisotropic displacement ellipsoids are set to 50% probability. (Colors were assigned as follows: Hydrogen in white, carbon in gray, oxygen in red and phosphorus in orange).

Even though we were not able to measure the NMR spectra of pure compound **1** in its closed form **1A** due to the observed equilibrium, the crystals obtained from THF/pentane, which were used for NMR analysis, were of sufficient quality for XRD measurement (Figure 2), confirming its structure.

**Scheme 5:**
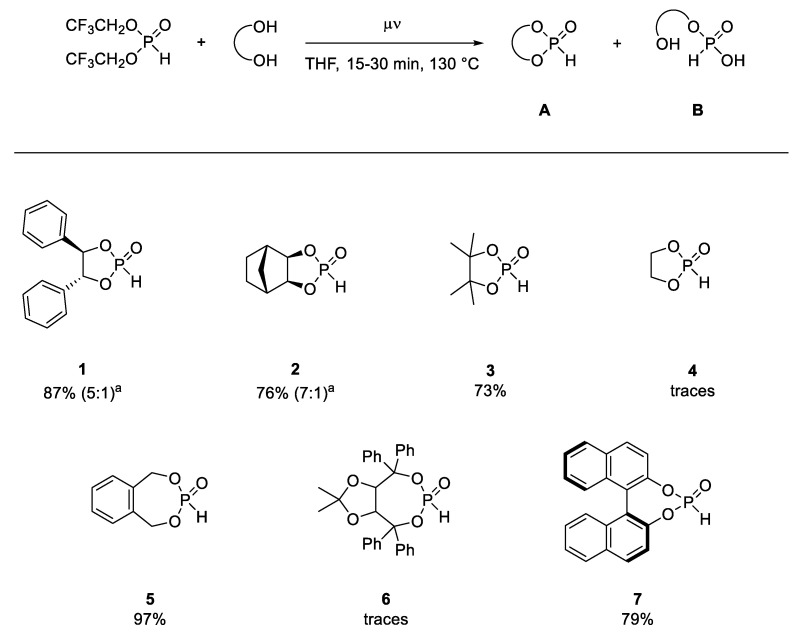
Scope of the synthesis of cyclic H-phosphonates. ^a^ Yield of closed form (**A**) and open form (**B**), calculated for the closed form (**A**) as sole product.

With our optimized reaction conditions in hand, we investigated the scope of this method (Scheme 5).

Good yields for most investigated diols were acquired under the previously established conditions, with the exception of phosphonates **4** and **6**. For the dioxaphospholane-based H-phosphonates, compound **2** showed similar behavior in the hydrolysis to compound **1**. H-phosphonate **3**, on the other hand, did not appear to be prone to hydrolysis at room temperature. For H-phosphonates **4** and **6**, the reaction resulted in an undefined mixture of side products under full conversion of BTFEP. Even a reduction in temperature and dilution of the reaction mixture did not lead to the formation of the desired compounds **4** and **6** in higher than trace amounts. In the case of H-phosphonate **6**, this was most probably caused by a disadvantageous geometry, which undermined the ring closing in favor of an intermolecular attack on an oligo-/polymeric product or stopping at the hetero-substituted product, with one trifluoroethanol moiety still intact. In the case of H-phosphonate **4,** polymerization during or after the formation of the cyclic phosphonate was expected, since the polymerization reaction of these unadorned phosphonates was established [27], albeit transesterification with diethyl H-phosphonate provides H-phosphonate **4** with good yields, as described in the literature [14]. This means that our conditions were most likely too harsh for the synthesis of this product. On the other hand, H-phosphonate **6** was easily prepared under *tert*-butanolysis conditions, without significant side products. Therefore, these two examples exemplify the limits of transesterification with BTFEP for the synthesis of cyclic H-phosphonates, representing a balancing act between too high and too low reactivity.

### 2.3. Synthesis of Dialkyl H-Phosphonates

With these results in hand, we continued to investigate this method for the synthesis of non-cyclic dialkyl H-phosphonates, without further optimization.

We were able to synthesize seven dialkyl H-phosphonates (Table 1). Alcohols with low molecular weight, i.e., methanol, ethanol and *iso*-propanol, did not react in a satisfactory or reproducible manner. This was due to the boiling points of these alcohols being below or similar to the boiling point of TFE. This might also be the reason for the comparatively low yields obtained with *n*-propanol and *iso*-butanol (Table 1, Entries 1 and 2). A decrease in yield was also observed for the sterically more demanding alcohols (Table 1, Entries 5–7). The increased steric demand caused a significantly decreased reaction rate in the second transesterification step (vide infra). While we observed that the complete conversion of BTFEP was fast, the second substitution required prolonged heating and did not proceed to completion even after extended reaction times. A slight excess of 0.2 equiv. of the alcohol improved the second substitution as well as reproducibility for all reactions; further increasing the amount of alcohol did not significantly improve the course of the reaction.

**Figure 3:**
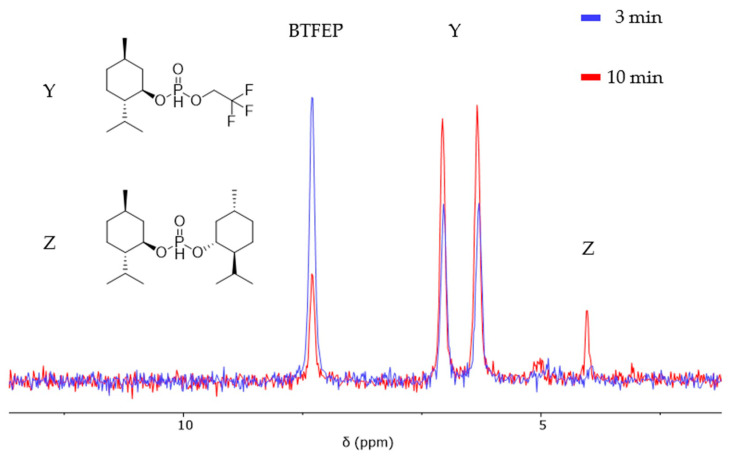
Reaction of two equivalents of (−)-menthol with BTFEP at 100 °C in THF.

Unlike the reaction with diols, the reaction proceeds in two distinct steps. In the first step, the chosen alcohol quickly exchanges the first TFE, while the second substitution proceeds more slowly, especially in the case of alcohols with a higher steric demand. The exemplary reaction of BTFEP with (−)-menthol in a 1:2 ratio (Figure 3) showed a pronounced generation of the mixed phosphonate **Y**; the second substitution appeared to be delayed until a threshold of approximately 50% was reached.

This opens a possible approach for the synthesis of mixed H-phosphonates by utilizing only one equivalent of alcohol. These mixed phosphonates kindled our interest because they allow the combination of a chiral moiety with the trifluoroethanol moiety, which proved to have an accelerating effect on H-phosphonate addition reactions in previous works [28,29,30]. Furthermore, TFE is a hydrolysis-labile substituent, which facilitates selective hydrolysis [31].

The previously mentioned work of Gibbs et al. [15] already described the synthesis of this type of hetero-substituted phosphonates, with good yield and selectivity, even though an excess of 2–3 equiv. BTFEP was used for the synthesis. In that regard, we saw an opportunity to reduce the required amount of phosphonate precursor and still remain in an additive-free environment.

Based on the previously established reaction conditions as a starting point, we varied the reaction parameters as well as the amount of BTFEP to optimize for the singular substitution reaction (Appendix B). Again, microwave irradiation provided good control over the progress of the reaction, whereby we were able to achieve mono-substitution reaction using equimolar ratios of the reactants (Table 2).

All reactions eventually stopped before full conversion was reached, without regard for the reaction temperature and concentration. Analysis of the reaction mixture showed that, in addition to the hetero-substituted product **Y**, an approximate 1:1 ratio of BTFEP and di-substituted H-phosphonate **Z** remained. The di-substitution consumed another equivalent of alcohol, thereby limiting the practical maximum yield. The observed rate of di-substitution varied depending on the alcohol and was consistent for the corresponding system. While the conversion of diphenyl H-phosphonate in pyridine was described as quantitative for the hetero- and di-substituted products [16], similar behavior was recently noticed in the reaction of dibenzyl H-phosphonate and *n*-butanol [32]. Increasing the amount of BTFEP up to 1.4 equiv. also did not provide any significant improvement in selectivity between mono- and di-substitution. It appears that a reverse reaction takes place, whereby the hetero-substituted H-phosphonate **Y** reacts with TFE to form back BTFEP; the liberated alcohol is then available to react with product **Y** to form the homo-di-substituted H-phosphonate **Z**. This results in about 10–25% of each BTFEP and homo-di-substituted phosphonate in the reaction mixture at the equilibrium state, which varies depending on the alcohol. This trend also manifests directly in their corresponding stability toward hydrolysis. Phosphonates **15–17** in particular tend to substitute both the introduced alcohol as well as the TFE moiety, with phosphonate **17** showing no selectivity between benzyl and TFE substitution, leading to a statistical distribution in the reaction mixture at the equilibrium state. In line with this, a considerable loss of yield of products **15**–**17** during purification was observed, with the best results being achieved via liquid chromatography at high R_F_-values of 0.8–0.9 in Diethylether. Phosphonates **18**–**20**, on the other hand, were more stable, with only the TFE moiety being labile to hydrolysis. A possible explanation for this is that increased steric hindrance might shield the phosphorus from hydrolysis, which would also be in line with the slightly increased yield from product **15** to **16**.

Furthermore, the introduction of chiral alcohols did not provide a diastereoisomeric excess in the corresponding product. This could be a sign that both diastereoisomeres are equally thermodynamically favored, and therefore, the interconversion via a P(III) species at elevated temperatures might lead to epimerization.

## 3. Materials and Methods

### 3.1. General Methods

All purchased solvents were distilled prior to use and freed of peroxides if necessary. Anhydrous THF was purchased from Acros Organics (Germany) (AcroSeal^®^ tetrahydrofuran stabilized over molecular sieve). Microwave reactions were carried out in a CEM Discover S system in sealed reaction vessels. Heating was controlled in dynamic mode with a maximum energy input of 300 W and under constant cooling and stirring. The coupling constants (*J*) are given in Hertz (Hz), and chemical shifts are denoted in parts per million (ppm) relative to the internal standard, chloroform (CHCl_3_) at 7.26 ppm in 1H-NMR and chloroform-d (CDCl_3_) at 77.16 ppm in ^13^C-NMR. ^31^P-NMR chemical shifts are given relative to the external standard, 85% H_3_PO_4_ at 0.00 ppm. The abbreviations s, d, t, q, h, m, dd, dt, dp, dtt refer to singlet, doublet, triplet, quartet, sextet, multiplet, doublet of doublets, doublet of triplets, doublet of quintet and doublet of triplets of triplets, respectively. Proton nuclear magnetic resonance (^1^H NMR, 400 MHz) spectra, carbon nuclear magnetic resonance (^13^C{^1^H}-NMR, 101 MHz) and phosphorous nuclear magnetic resonance spectra (^31^P-NMR, 162 MHz) were recorded on a Bruker “Avance II” or “Avance III HD” spectrometer. In situ yields were determined by taking an aliquot of 50–100 mg of the reaction mixture directly after reaction; the aliquot was diluted with CDCl_3_, and ^31^P-NMR were measured. The integration of all signals gave the corresponding yields in situ, which were reproducible and, according to the literature, should be accurate within 10% [11,24]. High-resolution mass measurements were carried out by using electrospray ionization (ESI) on a Bruker “MICROTOF LC“ spectrometer with methanol, except for compounds 1 and 4, which were measured with acetonitrile:formic acid (85%) in a 9:1 ratio as solvent. Column chromatography utilized Aldrich technical grade silica gel (60 Å, 230–400 mesh); for rinsing (General procedure B; H-phosphonates **8**–**11**), silica 60 M from Macherey-Nagel (60 Å, 230–400 mesh) was used. The utilized precursors bis(2,2,2 trifluoroethyl) H-phosphonate [33], (1R,2S,3R,4S) bicyclo[2.2.1]heptane-2,3-diol [34] and 1,2-Benzenedimethanol [35] were prepared according to procedures outlined in the literature.

### 3.2. General Procedure for the Synthesis of Cyclic H-Phosphonates ***1***–***7***

A 10 mL microwave vessel was charged (in order) with 1 mmol of diol, 2 mL of THF and 1.4 mmol of BTFEP. The microwave vessel was sealed with a snap cap and heated in a microwave reactor for 15–30 min at 130 °C in dynamic mode. Purification was accomplished by overlaying the reaction mixture with pentane for precipitation. If precipitation did not occur, 0.2 mL of methanol was added, and all volatiles were removed in vacuo. Afterward, the raw product was dissolved in as little THF as possible and precipitated with pentane, yielding the product.

*(4R,5R)-4,5-diphenyl-2-Hydro-2-oxo-1,3,2-dioxaphospholane* (**1**) (reaction time: 15 min); colorless needles; yield: 225 mg (0.87 mmol; 87%); ^1^H NMR (400 MHz, Chloroform-d) δ 7.97 (brs, 1H; B), 7.70 (d, *J* = 718.9 Hz, 1H; A), 7.43–7.34 (m, 6H; A), 7.32–7.27 (m, 2H; A), 7.23–7.12 (m, 8H; 2H A and 6 H B), 7.11–6.99 (m, 4H; B), 6.78 (d, *J* = 719.6 Hz, 1H; B), 5.43–5.33 (m, 2H; 1H A and 1H B), 5.32 (dd, *J* = 8.7, 2.3 Hz, 1H; A), 4.86 (d, *J* = 8.4 Hz, 1H; B) ppm; ^13^C NMR (101 MHz, Chloroform-d) δ 134.4 (d, *J* = 10 Hz; A), 133.7 (d, *J* = 8 Hz; A), 129.8 (A), 129.8 (A), 129.1 (A), 129.1 (A), 128.3 (B), 128.3 (B), 127.5 (B), 126.8 (A), 126.7 (A), 87.4 (A), 85.7 (A), 84.3 (d, *J* = 6 Hz; B), 78.4 (d, *J* = 5 Hz; B) ppm; ^31^P NMR (162 MHz, Chloroform-d) δ 20.02 (dd, *J* = 718.9, 2.6 Hz; A), 7.57 (dd, *J* = 719.8, 8.7 Hz; B) ppm; HRMS–ESI (Micro-Tof, *m*/*z*): closed: [M + Na]^+^ calcd for C_14_H_13_NaO_3_P, 283.0495; found, 283.0508; open: [M + Na]^+^ calcd for C_14_H_15_NaO_4_P, 301.0600; found, 301.0610.

*2-hydro-(3aR,4S,7R,7aS)-rel-4,7-Methano-2-oxo-1,3,2-hexahydro-benzodioxaphospholane* (**2**) (reaction time: 30 min); colorless solid; yield: 133 mg (0.76 mmol, 76%); ^1^H NMR (400 MHz, Chloroform-d) δ 7.33 (d, *J* = 720.8 Hz, 1H; B), 7.20 (d, *J* = 713.6 Hz, 1H; A), 4.58 (dd, *J* = 7.1, 1.6 Hz, 2H; B), 4.37 (dd, *J* = 9.8, 1.7 Hz, 2H; A), 2.52 (s, 2H; A), 2.42 (s, 2H; B), 2.03 (dt, *J* = 11.5, 2.0 Hz, 1H; A), 1.64–1.53 (m, 4H; 2H A and 2H B), 1.47 (dq, *J* = 11.4, 1.9 Hz, 1H; B), 1.41–1.30 (m, 2H; 1H A and 1H B), 1.16–1.07 (m, 2H; B), 1.06–0.97 (m, 2H; A) ppm; ^13^C NMR (101 MHz, Chloroform-d) δ 85.1 (d, *J* = 2 Hz; minor), 84.8 (major), 40.9 (m; both), 32.8 (minor), 31.7 (major), 22.9 (major), 22.7 (minor) ppm; ^31^P NMR (162 MHz, Chloroform-d) δ 24.34 (d, *J* = 720.8 Hz; minor), 23.46 (dt, *J* = 713.9, 9.7 Hz; major), 7.24 (open form) ppm; HRMS–ESI (Micro-Tof, m/z): [M + Na]^+^ calcd for C_14_H_13_NaO_3_P, 197.0338; found, 197.0339.

*2-Hydro-2-oxo-4,4,5,5-tetramethyl-1,3,2-dioxaphospholane* (**3**) (reaction time: 15 min); colorless crystals; yield: 119 mg (0.73 mmol; 73%); ^1^H NMR (400 MHz, Chloroform-d) δ 7.21 (d, *J* = 709.9 Hz, 1H), 1.47 (s, 6H), 1.37 (s, 6H) ppm; ^13^C NMR (101 MHz, Chloroform-d) δ 89.2, 24.7 (d, *J* = 4 Hz), 24.0 (d, *J* = 6 Hz) ppm; ^31^P NMR (162 MHz, Chloroform-d) δ 16.83 (d, *J* = 710.0 Hz) ppm; HRMS–ESI (Micro-Tof, *m*/*z*): [M + Na]^+^ calcd for C_6_H_13_NaO_3_P, 187.0495; found, 187.0497; analytical data are in agreement with the literature [2].

*(S)-Dinaphtho[2,1-d:1′,2′-f]-2-hydro-2-oxo-[1,3,2]dioxaphosphepine* (**5**) (reaction time: 30 min); colorless solid; yield: 262 mg (0.79 mmol; 79%); ^1^H NMR (400 MHz, Chloroform-d) δ 7.94 (dd, *J* = 8.9, 2.0 Hz, 2H), 7.86 (d, *J* = 8.3, 2H), 7.51 (dd, *J* = 8.9, 1.1 Hz, 1H), 7.47–7.34 (m, 3H), 7.32–7.10 (m, 4H), 7.20 (d, *J* = 732.0 Hz, 1H) ppm; ^13^C NMR (101 MHz, Chloroform-d) δ 145.7 (d, *J* = 10 Hz), 144.9 (d, *J* = 11 Hz), 132.6 (d, *J* = 1 Hz), 132.3 (d, *J* = 1 Hz), 132.0 (d, *J* = 1.0 Hz), 131.6, 131.5, 128.7, 128.6, 127.3, 127.2, 127.0, 127.0, 126.2, 126.1, 122.2 (d, *J* = 3 Hz), 121.7 (d, *J* = 3 Hz), 121.0 (d, *J* = 2 Hz), 120.2 (d, *J* = 3 Hz) ppm; ^31^P NMR (162 MHz, Chloroform-d) δ 13.83 (d, *J* = 732.1 Hz) ppm; HRMS–ESI (Micro-Tof, *m*/*z*): [M + H]^+^ calcd for C_20_H_14_O_3_P, 333.0675; found, 333.0677; analytical data are in agreement with the literature [36].

*2-Hydro-2-oxo-4,7-dihydro-1,3,2-benzodioxaphosphepine* (**7**) (reaction time: 15 min); colorless needles; yield: 178 mg (0.97 mmol; 97%); ^1^H NMR (400 MHz, Chloroform-d) δ 7.42–7.32 (m, 4H), 7.32–7.20 (m, 4H), 7.03 (d, *J* = 702.3 Hz, 1H), 5.40 (dd, *J* = 18.0, 13.9 Hz, 2H), 5.18 (dd, *J* = 15.6, 13.9 Hz, 2H) ppm; ^13^C NMR (101 MHz, Chloroform-d) δ 134.7, 129.1, 128.6, 66.3 (d, *J* = 6 Hz) ppm; ^31^P NMR (162 MHz, Chloroform-d) δ 13.09 (dtt, *J* = 702.3 Hz, *J* = 17.7, 15.7 Hz) ppm; HRMS–ESI (Micro-Tof, *m*/*z*): [M + Na]^+^ calcd for C_8_H_9_NaO_3_P, 207.0182; found, 207.0178; analytical data are in agreement with the literature [9].

### 3.3. General Procedure for the Synthesis of Di-Substituted H-Phosphonates ***8***–***14***

A 10 mL microwave vessel was charged (in order) with 2.2 mmol of alcohol, 1 mL of THF and 1 mmol of BTFEP. The microwave vessel was sealed with a snap cap and heated in a microwave reactor for 30 min at 130 °C in dynamic mode. In the case of phosphonates **8**–**11**, 2 mmol of water was added after the reaction, and the mixture was stirred for 20 min. The reaction mixture was subsequently rinsed through a short silica gel plug (2 cm wide, 0.5 cm thick) with 10 mL of a mixture of ethylacetate/cyclohexane (1:9). The solvent and excess alcohol were removed in vacuo at 90 mmHg (note: most of the short-chained dialkyl H-phosphonates are volatile; prolonged exposure to vacuum results in a considerable loss of product), yielding the dialkyl phosphites (Appendix A). H-phosphonates **12**, **13** and **14** were purified with column chromatography, with diethylether as the eluent; the remaining excess alcohol was subsequently removed via Kugelrohr distillation.

*Di-n-propyl H-phosphonate* (**8**) (reaction time: 30 min); colorless oil; yield: 122 mg (0.73 mmol; 73%); ^1^H NMR (400 MHz, Chloroform-d) δ 6.80 (d, *J* = 693.4 Hz, 1H), 4.08–3.96 (m, 4H), 1.71 (h, *J* = 7.1 Hz, 4H), 0.96 (t, *J* = 7.4 Hz, 6H) ppm; ^13^C NMR (101 MHz, Chloroform-d) δ 67.4 (d, *J* = 6 Hz), 23.9 (d, *J* = 6 Hz), 10.1 ppm; ^31^P NMR (162 MHz, Chloroform-d) δ 7.81 (dp, *J* = 693.5, 8.6 Hz) ppm; HRMS–ESI (Micro-Tof, *m*/*z*): Monomer: [M + Na]^+^ calcd for C_6_H_15_NaO_3_P, 189.0651; found, 189.0657; analytical data are in agreement with the literature [37].

*Di-i-butyl H-phosphonate* (**9**) (reaction time: 30 min); colorless oil; yield: 155 mg (0.80 mmol; 80%); ^1^H NMR (400 MHz, Chloroform-d) δ 6.82 (d, *J* = 693.9 Hz, 1H), 4.70–3.09 (m, 4H), 1.97 (hept, *J* = 6.7 Hz, 2H), 0.96 (d, *J* = 6.9 Hz, 12H) ppm; ^13^C NMR (101 MHz, Chloroform-d) δ 71.8 (d, *J* = 6 Hz), 29.3 (d, *J* = 6 Hz), 18.8 (d, *J* = 2 Hz) ppm; ^31^P NMR (162 MHz, Chloroform-d) δ 7.95 (dp, *J* = 694.0, 8.0 Hz) ppm; HRMS–ESI (Micro-Tof, *m*/*z*): Monomer [M + Na]^+^ calcd for C_8_H_19_NaO_3_P, 217.0964; found, 217.0968; analytical data are in agreement with the literature [11].

*Di-n-butyl H-phosphonate* (**10**) (reaction time: 30 min); colorless oil; yield 174 mg (0.90 mmol; 90%); ^1^H NMR (400 MHz, Chloroform-d) δ 6.78 (d, *J* = 692.3 Hz, 1H), 4.06 (td, *J* = 7.7, 7.2, 5.4 Hz, 4H), 2.01–1.58 (m, 4H), 1.40 (q, *J* = 7.6 Hz, 4H), 0.92 (t, *J* = 7.4 Hz, 7H) ppm; ^13^C NMR (101 MHz, Chloroform-d) δ 65.6 (d, *J* = 6 Hz), 32.5 (d, *J* = 6 Hz), 18.8, 13.6 ppm; ^31^P NMR (162 MHz, Chloroform-d) δ 7.79 (dp, *J* = 692.4, 8.4 Hz) ppm; HRMS–ESI (Micro-Tof, *m*/*z*): Monomer [M + Na]^+^ calcd for C_8_H_19_NaO_3_P, 217.0964; found, 217.0975; analytical data are in agreement with the literature [11].

*Di-i-amyl H-phosphonate* (**11**) (reaction time: 30 min); colorless oil; yield: 195 mg (0.88 mmol; 88%); ^1^H NMR (400 MHz, Chloroform-d) δ 6.77 (d, *J* = 692.5 Hz, 1H), 4.08 (dtd, *J* = 8.0, 6.7, 1.3 Hz, 4H), 1.72 (dth, *J* = 24.1, 13.4, 6.7 Hz, 2H), 1.56 (q, *J* = 6.8 Hz, 4H), 0.90 (d, *J* = 6.6 Hz, 12H) ppm; ^13^C NMR (101 MHz, Chloroform-d) δ 64.4 (d, *J* = 6 Hz), 39.2 (d, *J* = 6 Hz), 24.6, 22.4, 22.4 ppm; ^31^P NMR (162 MHz, Chloroform-d) δ 7.69 (dp, *J* = 692.6, 8.3 Hz) ppm; HRMS–ESI (Micro-Tof, *m*/*z*): Monomer [M + Na]^+^ calcd for C_10_H_23_NaO_3_P, 245.1277; found, 245.1279; analytical data are in agreement with the literature [38].

*Di-adamantyl H-phosphonate* (**12**) (reaction time: 90 min); colorless solid; yield: 233 mg (0.67 mmol; 67%); ^1^H NMR (400 MHz, Chloroform-d) δ 7.04 (d, *J* = 680.6 Hz, 1H), 2.18 (s, 6H), 2.16–2.04 (m, 12H), 1.71–1.57 (m, 12H) ppm; ^13^C NMR (101 MHz, Chloroform-d) δ 82.5 (d, *J* = 8 Hz), 44.2 (d, *J* = 5 Hz), 35.9, 31.2 ppm; ^31^P NMR (162 MHz, Chloroform-d) δ -4.04 (d, *J* = 680.2 Hz) ppm; HRMS–ESI (Micro-Tof, *m*/*z*): [M + Na]^+^ calcd for C_20_H_31_NaO_3_P, 373.1903; found, 373.1901; analytical data are in agreement with the literature [11].

*Di-(−)-menthyl H-phosphonate* (**13**) (reaction time: 90 min); colorless oil; yield: 289 mg (0.81 mmol; 81%); ^1^H NMR (400 MHz, Chloroform-d) δ 6.88 (d, *J* = 686.9 Hz, 1H), 4.21 (m, 2H), 2.23–1.98 (m, 4H), 1.65 (dt, *J* = 11.8, 2.7 Hz, 4H), 1.52–1.38 (m, 1H), 1.34 (m, 2H), 1.26–1.11 (m, 2H), 1.05–0.93 (m, 3H), 0.90 (2 d, *J* = 6,7 Hz and *J* = 7 Hz, 6H), 0.89 (d, *J* = 1.8 Hz, 7H), 0.87–0.81 (m, 1H), 0.80 (d, *J* = 1.7 Hz, 3H), 0.79 (2 d, *J* = 7 Hz and *J* = 6,7 Hz, 3H) ppm; ^13^C NMR (101 MHz, Chloroform-d) δ 78.0 (d, *J* = 7 Hz), 77.9 (d, *J* = 7 Hz), 48.5 (d, *J* = 3 Hz), 48.4 (d, *J* = 3 Hz), 43.5, 43.0 (d, *J* = 1 Hz), 34.1, 34.1, 31.7, 31.7, 25.9, 25.6, 23.0, 23.0, 22.9, 22.0, 21.0, 21.0, 15.8, 15.8 ppm; ^31^P NMR (162 MHz, Chloroform-d) δ 5.39 (dt, *J* = 686.8, 8.7 Hz) ppm; HRMS–ESI (Micro-Tof, *m*/*z*): [M + Na]^+^ calcd for C_20_H_39_NaO_3_P, 381.2529; found, 381.2531; analytical data are in agreement with the literature [11].

*Di-(+)-α-fenchyl H-phosphonate* (**14**) (reaction time: 90 min); colorless oil; yield: 231 mg (0.65 mmol; 65%); ^1^H NMR (400 MHz, Chloroform-d) δ 6.80 (d, *J* = 686.9 Hz, 1H), 3.93 (dd, *J* = 24.5, 11.1 Hz, 2H), 1.75–1.58 (m, 6H), 1.50–1.43 (m, 2H), 1.42–1.32 (m, 2H), 1.12 (dd, *J* = 10.6, 4.5 Hz, 2H), 1.06 (d, *J* = 8.4 Hz, 6H), 1.00 (s, 8H), 0.87 (d, *J* = 3.1 Hz, 6H) ppm; ^13^C NMR (101 MHz, Chloroform-d) δ 89.6 (d, *J* = 7 Hz), 89.4 (d, *J* = 7 Hz), 49.3 (d, *J* = 5 Hz), 48.1, 41.1, 41.0, 39.7–39.5 (m), 30.1, 29.8, 26.0, 25.9 (d, *J* = 2 Hz), 21.5 (d, *J* = 2 Hz), 19.5, 19.4 ppm; ^31^P NMR (162 MHz, Chloroform-d) δ 8.13 (dt, *J* = 686.8, 11.1 Hz) ppm; HRMS–ESI (Micro-Tof, *m*/*z*): [M + Na]^+^ calcd for C_20_H_35_NaO_3_P, 377.2216; found, 377.2214; analytical data are in agreement with the literature [11].

### 3.4. General Procedure for the Synthesis of H-Phosphonates ***15***–***20***

A 10 mL microwave vessel was charged (in order) with 4 mmol of alcohol, 4 mL of THF and 4 mmol of BTFEP. The microwave vessel was sealed with a snap cap and heated in a microwave reactor for 30 min (in the case of H-phosphonates **15**, **16** and **17**) or 60 min (in the case of H-phosphonates **18**, **19** and **20**) at 130 °C in dynamic mode. The reaction solvent and all volatiles were removed in vacuo. Afterward, column chromatography with diethylether (H-phosphonates **15**, **16** and **17**) or DCM (H-phosphonates **18**, **19** and **20**) yielded the hetero-substituted dialkyl phosphites (Appendix B).

*Trifluoroethyl-n-butyl H-phosphonate* (**15**) (reaction time: 30 min); colorless oil; yield: 306 mg (1.31 mmol; 35%); ^1^H NMR (400 MHz, Chloroform-d) δ 6.90 (d, *J* = 724.0 Hz, 1H), 4.39 (dq, *J* = 9.6, 8.1 Hz, 2H), 4.27–3.98 (m, 2H), 1.72–1.63 (m, 2H), 1.40 (h, *J* = 7.4 Hz, 2H), 0.93 (t, *J* = 7.4 Hz, 3H) ppm. ^13^C NMR (101 MHz, Chloroform-d) δ 122.8 (qd, *J* = 278, 7 Hz), 66.3 (d, *J* = 7 Hz), 61.9 (qd, *J* = 38, 5 Hz), 32.3 (d, *J* = 6 Hz), 18.7, 13.5 ppm; ^31^P NMR (162 MHz, Chloroform-d) δ 7.64 (dp, *J* = 724.3, 9.2 Hz) ppm; HRMS–ESI (Micro-Tof, *m*/*z*): [M + Na]^+^ calcd for C_6_H_12_F_3_NaO_3_P, 243.0368; found, 243.0363.

*Trifluoroethyl-i-amyl H-phosphonate* (**16**) (reaction time: 30 min); colorless oil; yield: 389 mg (1.66 mmol; 42%); ^1^H NMR (400 MHz, Chloroform-d) δ 6.90 (d, *J* = 723.4 Hz, 1H), 4.55–4.26 (m, 2H), 4.24–4.02 (m, 2H), 1.73 (dp, *J* = 13.3, 6.7 Hz, 1H), 1.59 (q, *J* = 6.8 Hz, 2H), 0.92 (d, *J* = 6.6 Hz, 6H) ppm; ^13^C NMR (101 MHz, Chloroform-d) δ 122.8 (qd, *J* = 278, 7 Hz), 65.1 (d, *J* = 7 Hz), 61.9 (qd, *J* = 38, 5 Hz), 39.0 (d, *J* = 6 Hz), 24.6, 22.3 ppm; ^31^P NMR (162 MHz, Chloroform-d) δ 7.61 (dp, *J* = 723.2, 9.2 Hz) ppm; HRMS–ESI (Micro-Tof, *m*/*z*): [M + Na]^+^ calcd for C_7_H_14_F_3_NaO_3_P, 257.0525; found, 257.052.

*Trifluoroethyl-benzyl H-phosphonate* (**17**) (reaction time: 30 min); colorless oil; yield: 288 mg (1.13 mmol; 28%); ^1^H NMR (400 MHz, Chloroform-d) δ 7.42–7.32 (m, 5H), 6.98 (d, *J* = 731.0 Hz, 1H), 5.21–5.09 (m, 2H), 4.42–4.19 (m, 2H) ppm; ^13^C NMR (101 MHz, Chloroform-d) δ 135.0 (d, *J* = 6 Hz), 129.2, 129.0, 128.4, 122.7 (qd, *J* = 278, 8 Hz), 68.1 (d, *J* = 6 Hz), 61.8 (qd, *J* = 38, 5 Hz) ppm; ^31^P NMR (162 MHz, Chloroform-d) δ 7.70 (dp, *J* = 731.4, 9.8 Hz) ppm; HRMS–ESI (Micro-Tof, *m*/*z*): [M + Na]^+^ calcd for C_9_H_10_F_3_NaO_3_P, 277.0212; found, 277.0218.

*Trifluoroethyl-(−)-menthyl H-phosphonate* (**18**) (reaction time: 60 min); colorless oil (R_F_: 0.32 in DCM; yield: 974 mg (3.22 mmol; 81%); ^1^H NMR (400 MHz, Chloroform-d) δ 6.96 (2 d, *J* = 720.4 and *J* = 720.7 Hz, 1H), 4.52–4.21 (m, 3H), 2.23–2.00 (m, 2H), 1.74–1.62 (m, 2H), 1.53–1.34 (m, 2H), 1.31–1.17 (m, 1H), 1.08–0.95 (m, 1H), 0.95–0.90 (m, 6H), 0.90–0.84 (m, 1H), 0.80 (t, *J* = 6.7 Hz, 3H) ppm; ^13^C NMR (101 MHz, Chloroform-d) δ 124.9–120.9 (m), 79.7 (d, *J* = 7 Hz), 79.5 (d, *J* = 7 Hz), 62.4–61.2 (m), 48.3, 48.3, 43.2, 43.1, 33.9, 33.9, 31.7, 26.0, 25.7, 23.0, 22.0, 22.0, 21.0, 20.9, 15.7, 15.6 ppm; ^31^P NMR (162 MHz, Chloroform-d) δ 6.71 (dq, *J* = 720.9, 9.5 Hz), 6.30 (dq, *J* = 720.2, 9.0 Hz) ppm; HRMS–ESI (Micro-Tof, *m*/*z*): [M + Na]^+^ calcd for C_12_H_22_F_3_NaO_3_P, 325.1151; found, 325.1149.

*Trifluoroethyl-(+)-α-fenchyl H-phosphonate* (**19**) (reaction time: 60 min); colorless oil (R_F_: 0.26 in DCM); yield: 936 mg (3.12 mmol; 78%); ^1^H NMR (400 MHz, Chloroform-d) δ 6.96 (2 d, *J* = 721.2 Hz and *J* = 723.9 Hz, 1H), 4.52–4.30 (m, 2H), 4.05 (ddd, *J* = 11.0, 4.3, 1.9 Hz, 1H), 1.83–1.63 (m, 3H), 1.57–1.40 (m, 2H), 1.26–1.20 (m, 1H), 1.11 (d, *J* = 7.3 Hz, 4H), 1.05 (d, *J* = 6.1 Hz, 3H), 0.92 (d, *J* = 6.2 Hz, 3H) ppm; ^13^C NMR (101 MHz, Chloroform-d) δ 124.3–121.0 (m), 90.6 (d, *J* = 4 Hz), 90.5 (d, *J* = 4 Hz), 62.0 (qd, *J* = 38, 5 Hz), 49.3 (d, *J* = 5 Hz), 49.2 (d, *J* = 5 Hz), 48.0, 47.9, 41.1, 41.0, 39.6 (d, *J* = 2 Hz), 39.6 (d, *J* = 3 Hz), 29.8, 29.7, 26.0, 25.9, 25.8, 25.7, 21.3, 21.1, 19.2, 19.1 ppm; ^31^P NMR (162 MHz, Chloroform-d) δ 7.62, 7.53 ppm*; HRMS–ESI (Micro-Tof, *m*/*z*): [M + Na]^+^ calcd for C_12_H_20_F_3_NaO_3_P, 323.0994; found, 323.0996. * Coupling constants could not be determined because of overlaying signals (see Supplementary Materials, attached spectra, page S56).

*Trifluoroethyl-adamantyl H-phosphonate* (**20**) (reaction time: 60 min); colorless oil (R_F_: 0.20 in DCM); yield: 872 mg (2.92 mmol; 73%); ^1^H NMR (400 MHz, Chloroform-d) δ 6.98 (d, *J* = 717.7 Hz, 1H), 4.45–4.25 (m, 2H), 2.20 (s, 3H), 2.09 (d, *J* = 3.0 Hz 6H), 1.63 (t, *J* = 3.1 Hz 6H) ppm; ^13^C NMR (101 MHz, Chloroform-d) δ 134.9–109.9 (m), 85.4 (d, *J* = 9 Hz), 61.6 (qd, *J* = 38, 5 Hz), 44.2 (d, *J* = 4 Hz), 35.6, 31.3 ppm; ^31^P NMR (162 MHz, Chloroform-d) δ 2.13 (dt, *J* = 717.8, 9.3 Hz) ppm; HRMS–ESI (Micro-Tof, *m*/*z*): [M + Na]^+^ calcd for C_12_H_18_F_3_NaO_3_P, 321.0838; found, 321.0838.

## 4. Conclusions

In conclusion, we were able to utilize the transesterification of bis-(2,2,2-trifluoroethyl) H-phosphonate (BTFEP) under microwave conditions to synthesize seven homo- and six hetero-di-substituted dialkyl H-phosphonates, as well as five cyclic H-phosphonates. In the case of reaction with two monohydric alcohols, full conversion of BTFEP was accomplished, with the limiting factor in the reaction time being the second alcoholysis reaction to the di-substituted H-phosphonate. The deceleration in the second alcoholysis step enabled the selective synthesis of mixed phosphonates by introducing only one equivalent of alcohol. The synthesis of cyclic H-phosphonates was readily accomplished, and the reactivity of the dioxaphospholane-based phosphonates toward water was described. Therefore, this method exhibited good results for all three types of H-phosphonates. In comparison to previous methods, we were able to apply lower reaction times and temperatures, combined with preparation under ambient conditions employed in previous microwave methodologies. We are currently working on utilizing these novel H-phosphonates for the synthesis of α-aminophosphonates.

## Figures and Tables

**Table 1:** Scope for the synthesis of homo-dialkyl H-phosphonates.

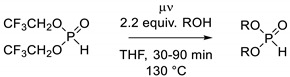					
Entry	R	Product	t [min]	Yield In Situ ^a^ [%]	Isolated Yield [%]
1	nPr	8	30	76	73
2	iBu	9	30	78	80
3	nBu	10	30	86	90
4	iAmyl	11	30	87	88
5	1-Adamantyl	12	90	74	67
6	(−)-Menthyl	13	90	83	81
7	(+)-Fenchyl	14	90	74	65

^a^ Yields estimated via integration of all signals in ^31^P-NMR. Samples were taken directly from the reaction mixture after completion of reaction runtime, without any purification steps (accurate within ~10% [24]).

**Table 2:** Scope for the synthesis of hetero-dialkyl H-phosphonates.

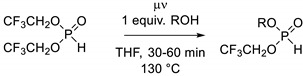					
Entry	R	Product	t [min]	Yield In Situ ^a^ [%]	Isolated Yield [%]
1	*n*Bu	15	30	64	35
2	*i*Amyl	16	30	74	42
3	Benzyl	17	30	50	28
4	(−)-Menthyl	18	60	81	81
5	(+)-Fenchyl	19	60	74	78
6	1-Adamantyl	20	60	78	73

^a^ Yields estimated via integration of all signals in ^31^P-NMR. Samples were taken directly from the reaction mixture after completion of reaction runtime, without any purification steps (accurate within ~10% [24]).

## Data Availability

The crystallographic data have been deposited at the Cambridge Crystallographic Data Centre as CCDC No. 2036186 and can be obtained free of charge (https://www.ccdc.cam.ac.uk/structures/).

## Supplementary Materials

The following are available online at https://www.mdpi.com/article/10.3390/molecules29112432/s1, ^1^H-NMR spectra, ^13^C-NMR spectra, ^31^P-NMR spectra, crystallographic data.

## Appendix A

Several attempts were undertaken to optimize the synthesis of cyclic H-phosphonate 1 (Table A1). While the variables of time, temperature and reaction time were varied, we decided upon THF as the solvent early into our investigation. The reasoning behind this decision was two-fold. On the one hand, the temperatures necessary prohibited low-boiling solvents from being used due to overpressure in the sealed reaction vessel. On the other hand, early direct crystallization from the reaction mixture with unipolar solvents, i.e., pentane, toluene, etc., excluded immiscible solvents for the reaction. As an alternative, 1,4-dioxane showed similar-to-identical reactivity and results in our research.

**Table A1:** Optimization of the synthesis of cyclic H-phosphonate **1**.

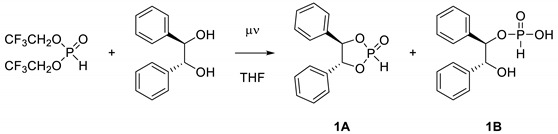

Entry	Equivalent BTFEP	Concentration [Mol/L]	T [°C]	t [min]	Isolated Yield ^a^
1	1	0.5	130	30	61
2	1.4	0.5	130	15	87
3	1.4	0.5	100	15	72
4	1.4	1	100	30	82
5	1.4	1	130	10	73

^a^ Yield of closed form (**1A**) and open form (**1B**), calculated for the closed form (**1A**) as sole product.

## Appendix B

The hetero-substitution reaction was optimized in the synthesis of trifluoroethyl-(−)-menthyl H-phosphonate (Y) (Table A2).

**Table A2:** Optimization of the synthesis of hetero-substituted H-phosphonates.

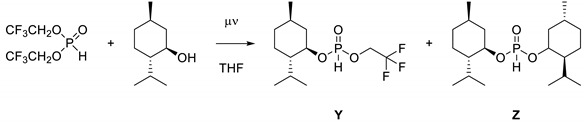

Entry	Equivalent BTFEP	t [min]	T [°C]	Conversion [%]	Yield In Situ ^a^ [%]	Ratio Y:Z
1	1	20	100	57	53	24:1
2	1	40	100	86	73	11:1
3	1	60	100	90	72	7:1
4	1	120	100	87	60	5:1
5	1.05	30	100	79 ^b^	68	11:1
6	1.1	30	110	90 ^b^	79	12:1
7	1	60	90	75	66	12:1
8	1	60	130	93	81	13:1

^a^ Yields estimated via integration of all signals in ^31^P-NMR. Samples were taken directly from the reaction mixture after completion of reaction runtime, without any purification steps (accurate within ~10% [24]); ^b^ Excess BTFEP was calculated for a given conversion for increased legibility.

Variation in BTFEP loading did not favorably influence the reaction outcome, with non-significant deviation for the ratio between Z and Y in all cases. Solely the progress of the reaction appeared to be the determining factor for the generation of the di-substituted H-phosphonate. It is noteworthy that prolonged heating time led to decomposition, with 1 h being determined as the maximum feasible time before decomposition overtook the generation of the desired product (Table A2, Entries 1–4). The probable decomposition pathway was hydrolysis of the phosphonate-Z-yielding mono-substituted (−)-menthyl phosphonate. The combination of elevated temperatures of 130 °C as well as an equimolar amount of BTFEP in alcohol gave the most satisfactory balance of conversion, the ratio of both H-phosphonate products and the overall yield (Table A2, Entry 8).

## Appendix C

We found that a quick removal of volatiles via the rotary evaporator directly after reaction yielded products 8–11 with a workable purity and slightly higher yields than those determined via in situ NMR measurements. For further use, this might be sufficient as a purification step, but we also found a considerable fluctuation in the yields with the length of evaporation, to a degree where reproducibility was generally not provided. While for a gram scale, fractioned distillation might be the most straightforward purification method, we decided to develop a purification method for a low mmol scale. Because trifluoroethanol is quickly exchanged, as previously established, and since dialkyl phosphites, even though susceptible to hydrolysis, are stable against water for a short period of time, we came to the conclusion that the addition of water will lead to a quick hydrolysis of mixed H-phosphonates without significantly decomposing our target product. This in and of itself would not alleviate the problem of removing these undesired side products, but it enabled us to quickly remove them via rinsing through a short silica plug, thereby leaving solely the solvents, excess alcohol and trifluoroethanol as impurities. The second problem with purification is the removal of alcohols, especially in the case of the more volatile H-phosphonates, which are easily co-evaporated. In this case, azeotropic removal of alcohol with cyclohexane presented itself as a quick and efficient method. With this method in hand, we were able to reproducibly purify the H-phosphonates, with close to maximum yield, as given in the in situ NMRs.

## Appendix D

It was observed that prolonged exposure of all hetero-di-substituted H-phosphonates to the stationary phase did lead to a considerable loss of product. From our experience, the runtime of liquid chromatography should not considerably exceed 5 min.
