# Supplementary material for: Bis(2,2,2 trifluoroethyl) Phosphonate as a Convenient Precursor for the Synthesis of H-Phosphonates
Source: Molecules. 2024 May 21;29(11):2432. doi: 10.3390/molecules29112432 (PMC11173472; doi:10.3390/molecules29112432)
Supplement: Supplementary file 1 [file molecules-29-02432-s001.zip › molecules-3004143-supplementary.pdf]

Supporting Information

For

Bis-(2,2,2-trifluoroethyl) Phosphonate as a Convenient Precursor for the Synthesis of H-Phosphonates

Jean-Marie Pohl [1], Fabian Stöhr [1], Tim Kramer [1], Jonathan Becker [2]

and Richard Göttlich [1,*]

Address: [1] Institute for Organic Chemistry, Justus-Liebig-Universität Giessen, Heinrich-Buff-Ring 17, 35392 Giessen, Germany

[2] Institute for Inorganic and Analytical Chemistry, Justus-Liebig-Universität Giessen, Heinrich-Buff-Ring 17, 35392 Giessen, Germany

* Correspondence: richard.goettlich@org.chemie.uni-giessen.de

NMR Spectra and Crystallographic Data

Table of Contents

Page

**NMR spectra**	S2–S59
**Crystallographic data of 1**	S60–S66
**References**	S67

^{1}H-NMR spectra of 1

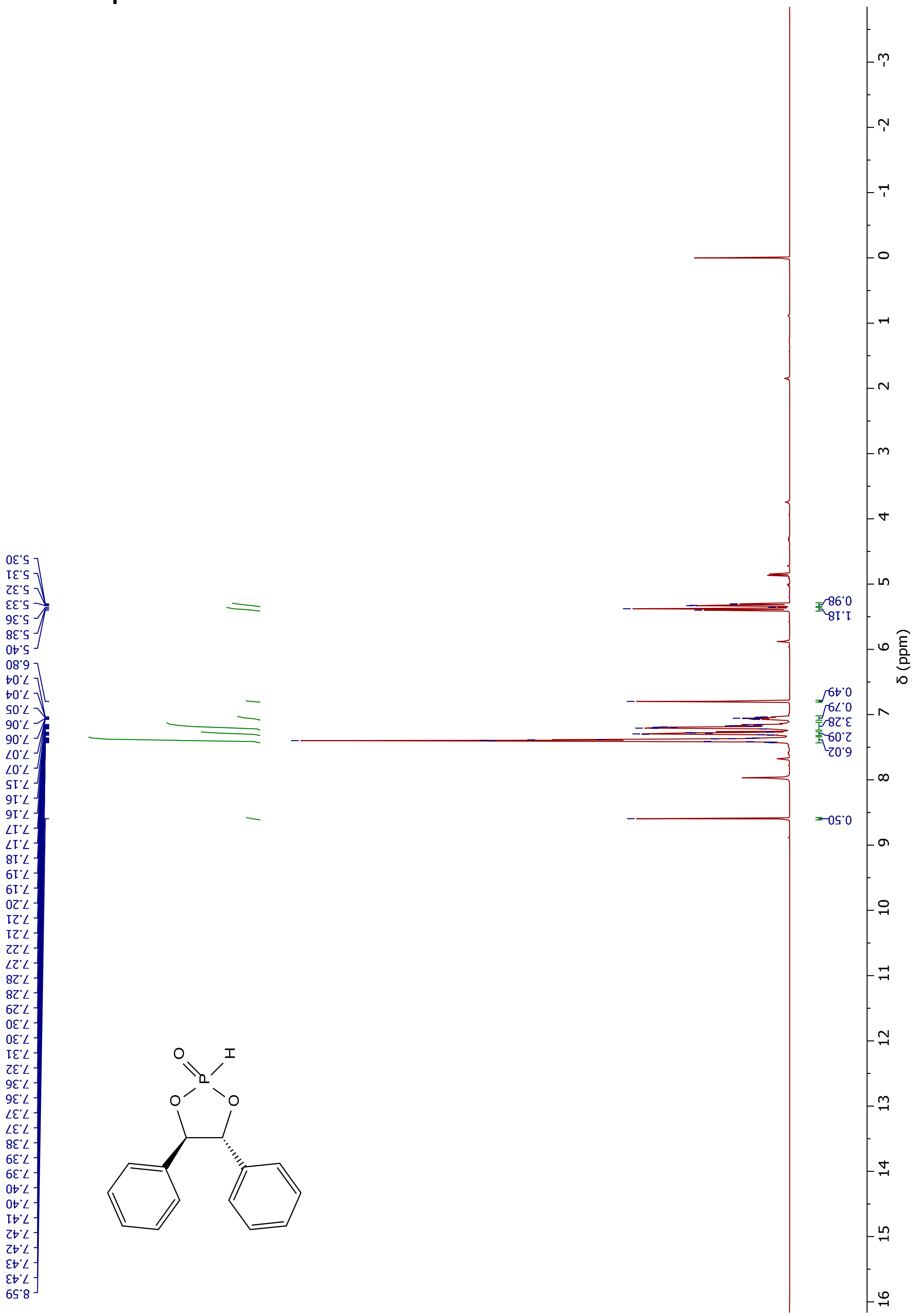

^{1}H-NMR spectra of 1 (detail)

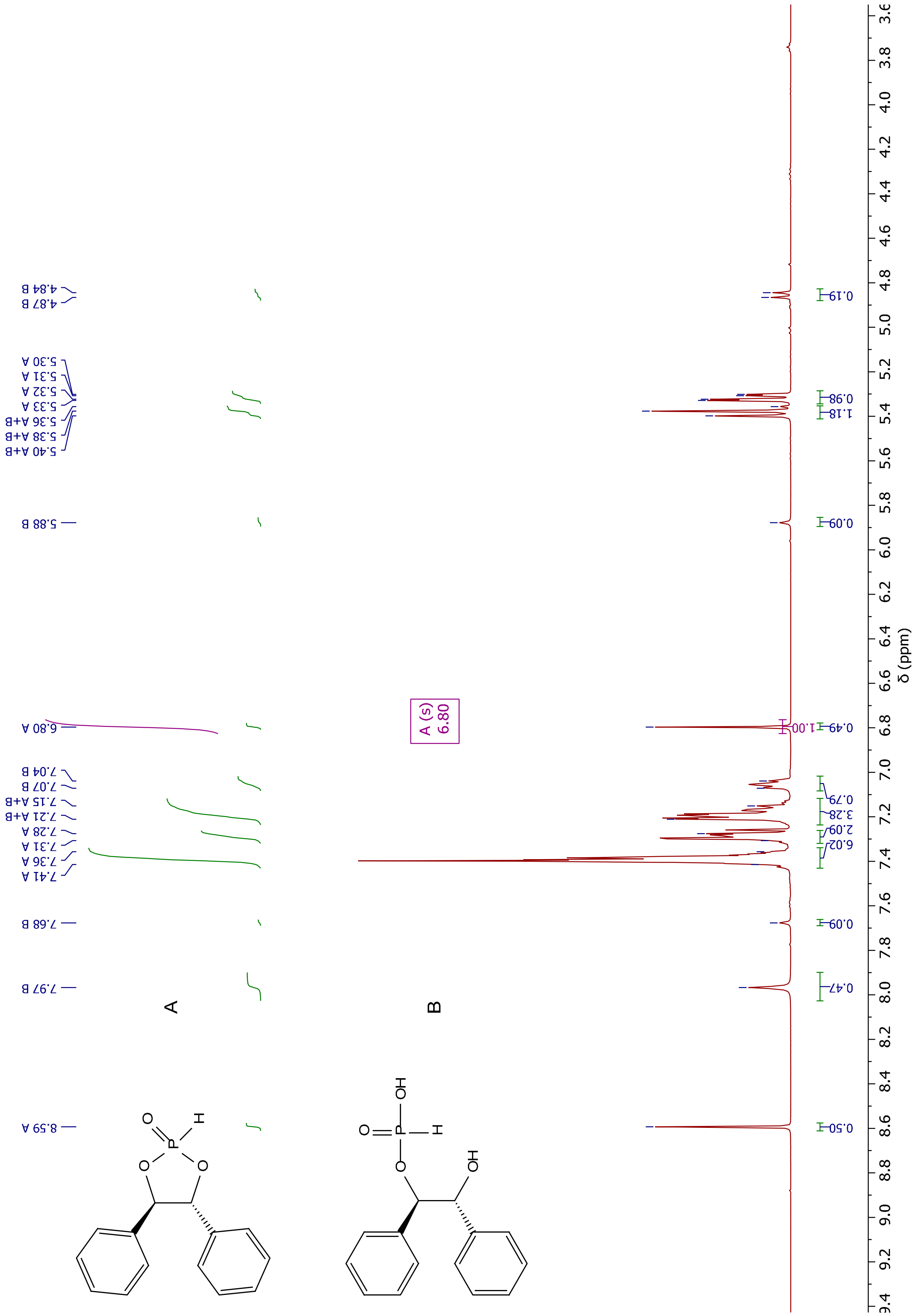

$^{13}C\{^1H\}$-NMR spectra of 1 (cyclic form A)

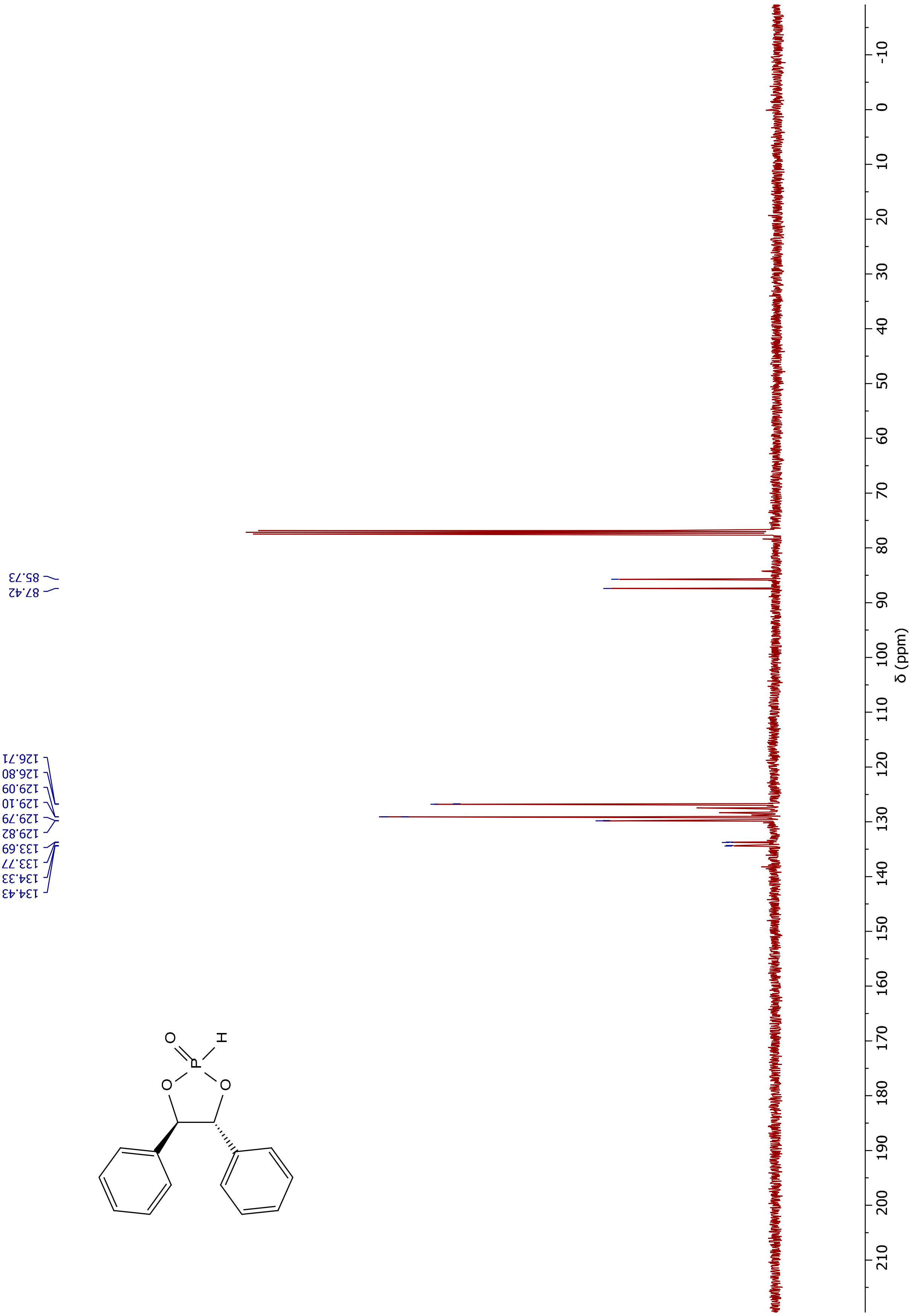

^{1}H-NMR spectra of 1 (open form B, not all peaks visible)

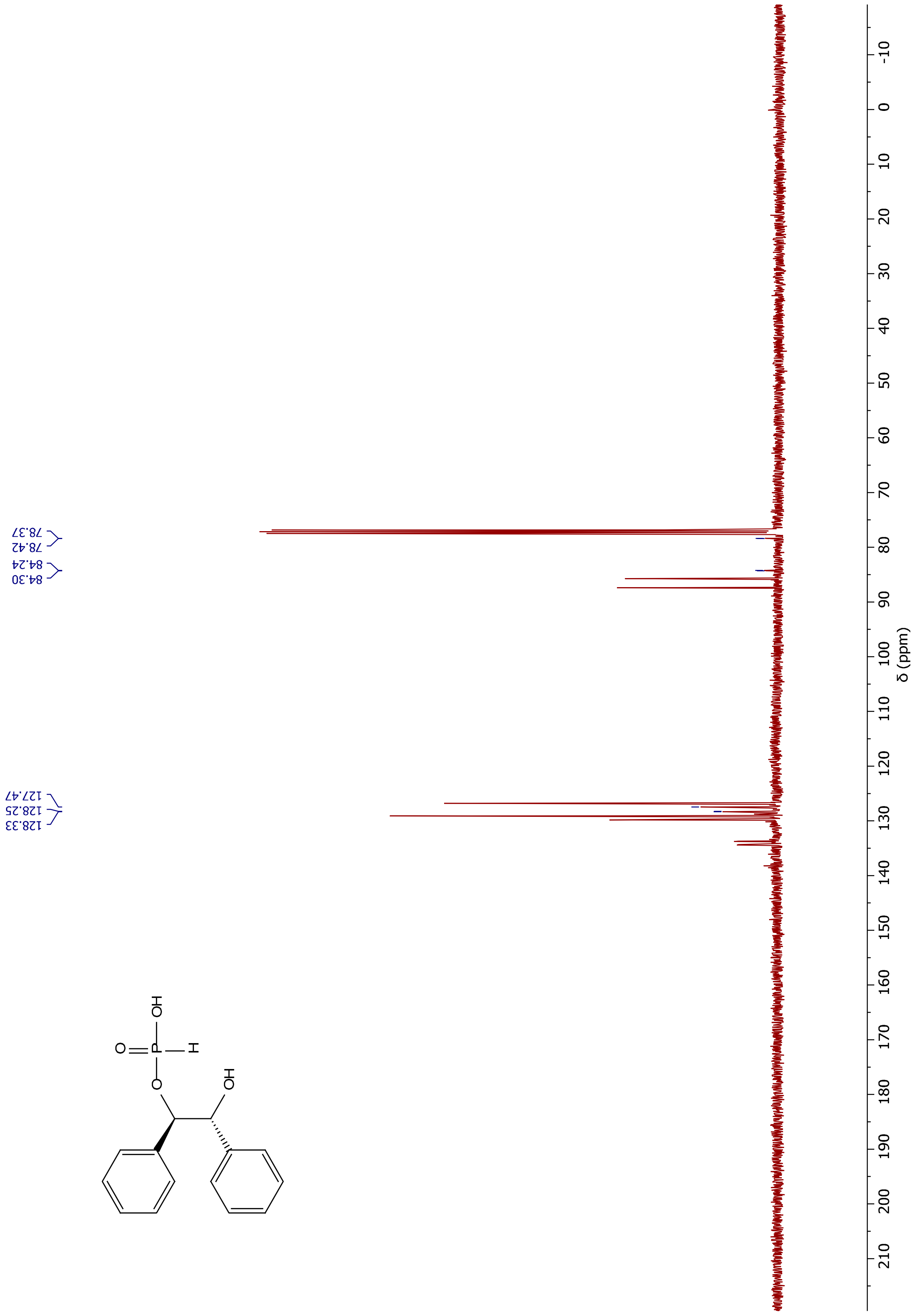

$^{31}P\{^1H\}$-NMR spectra of 1

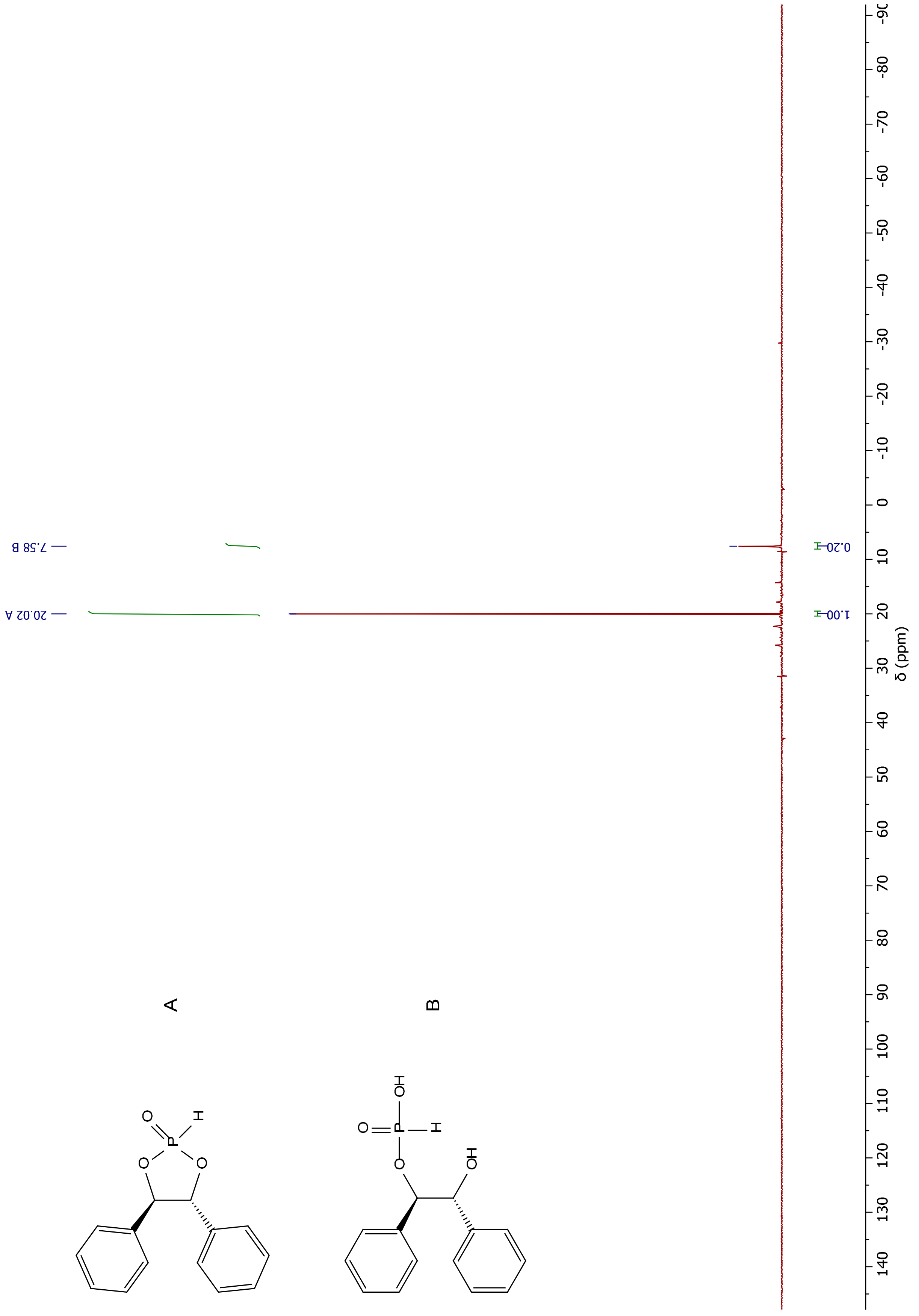

^{1}H-NMR spectra of 2

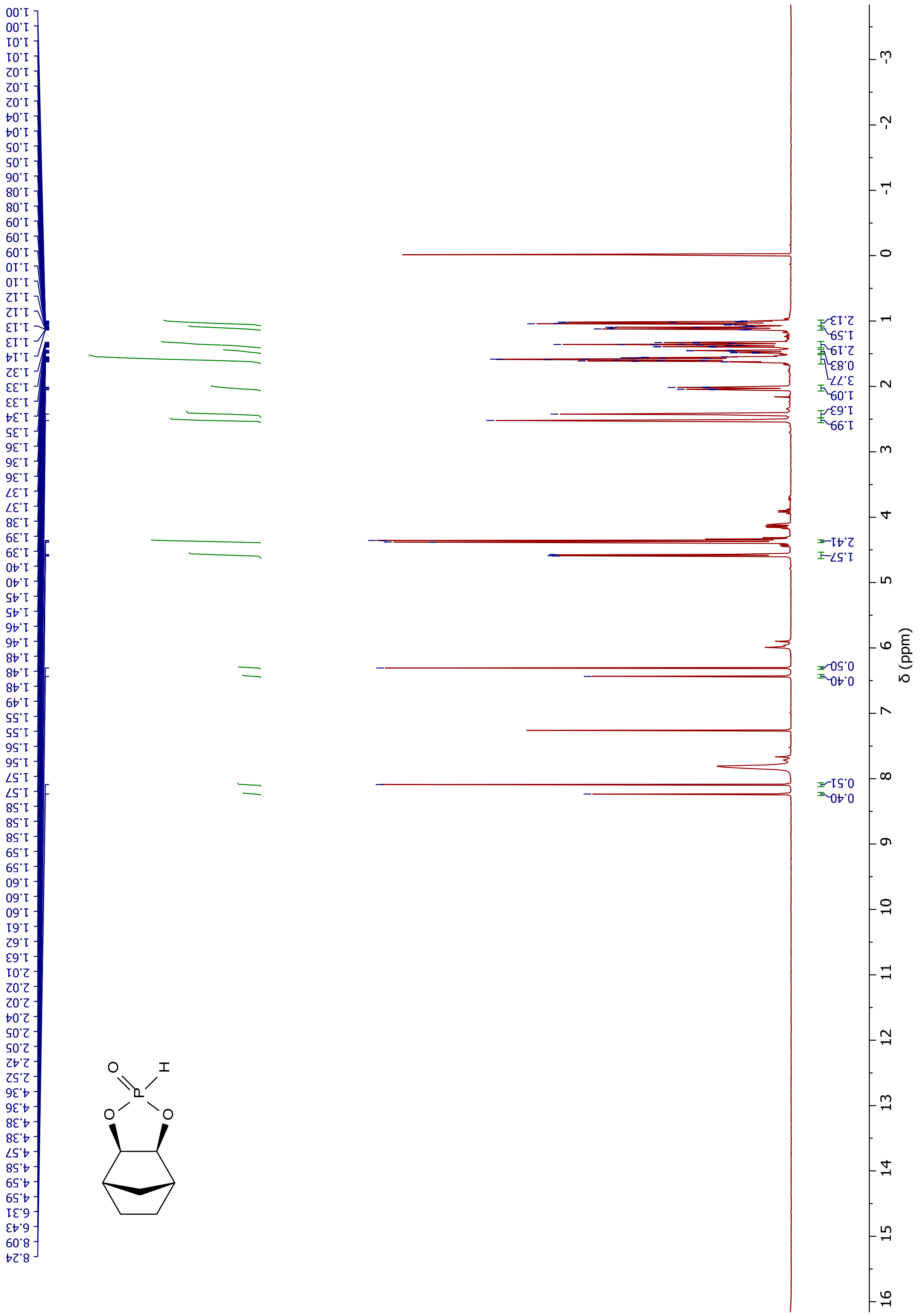

^{1}H-NMR spectra of 2 (detail)

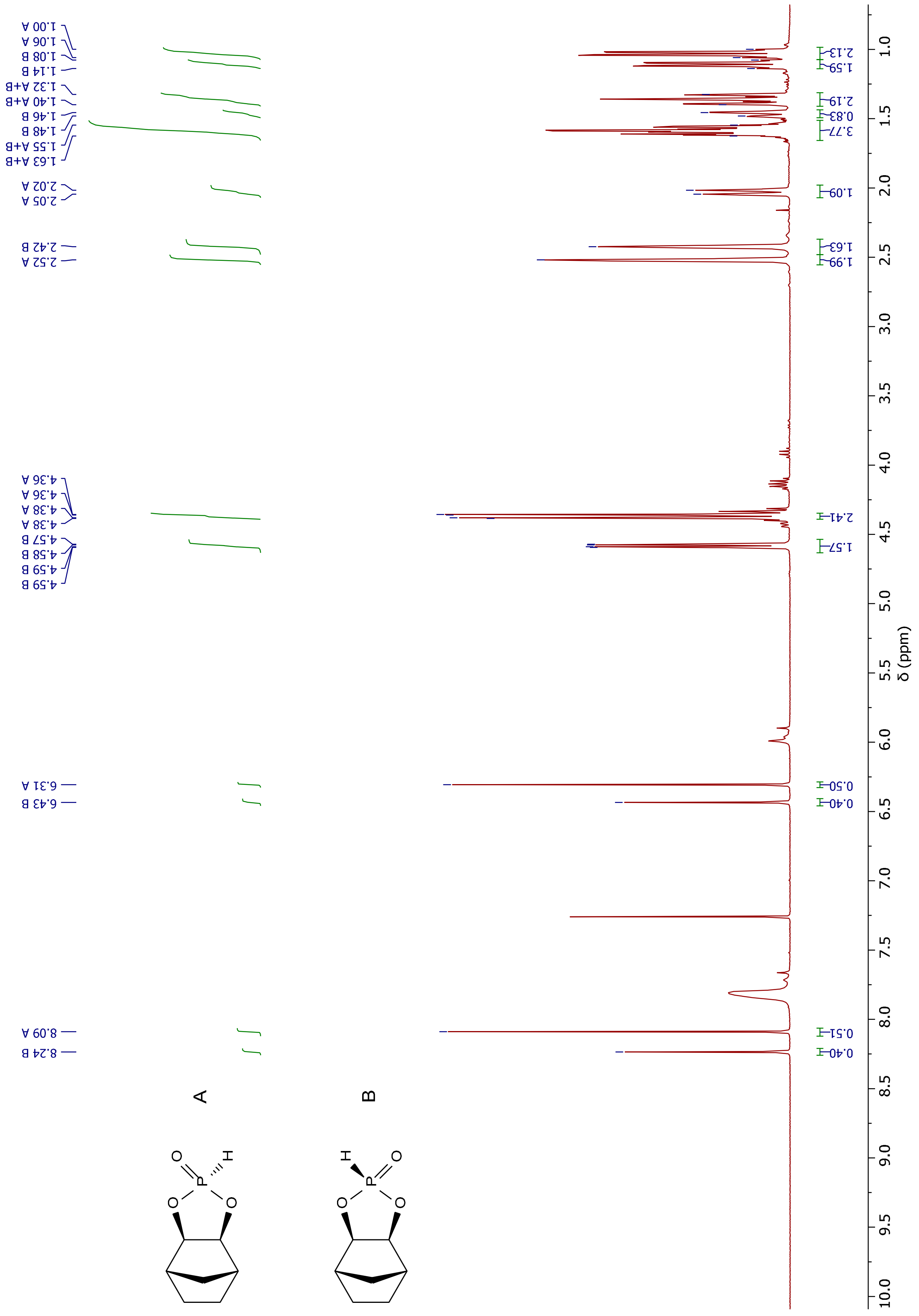

$^{13}C\{^{1}H\}$-NMR spectra of 2

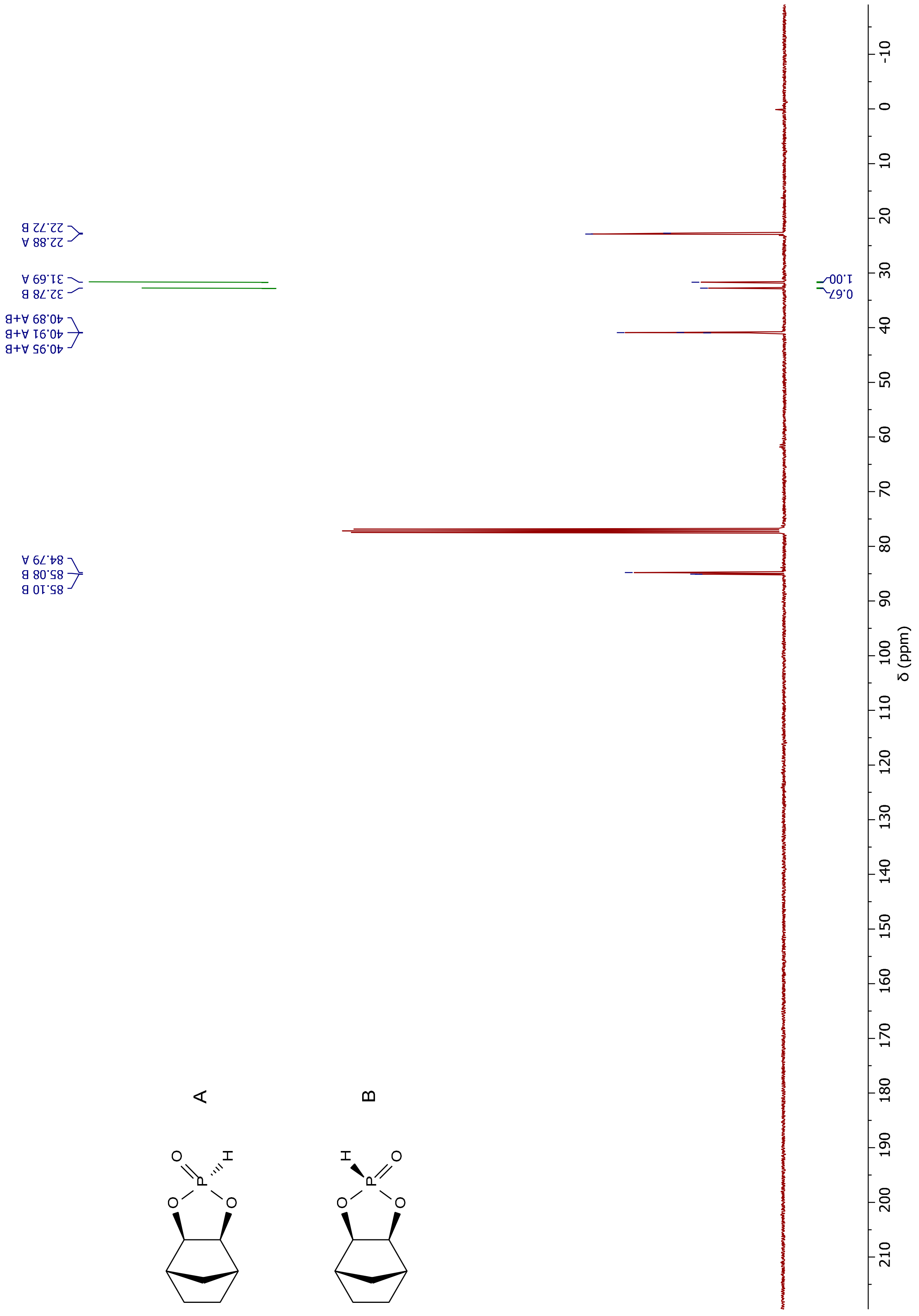

$^{31}P\{^{1}H\}$-NMR spectra of 2

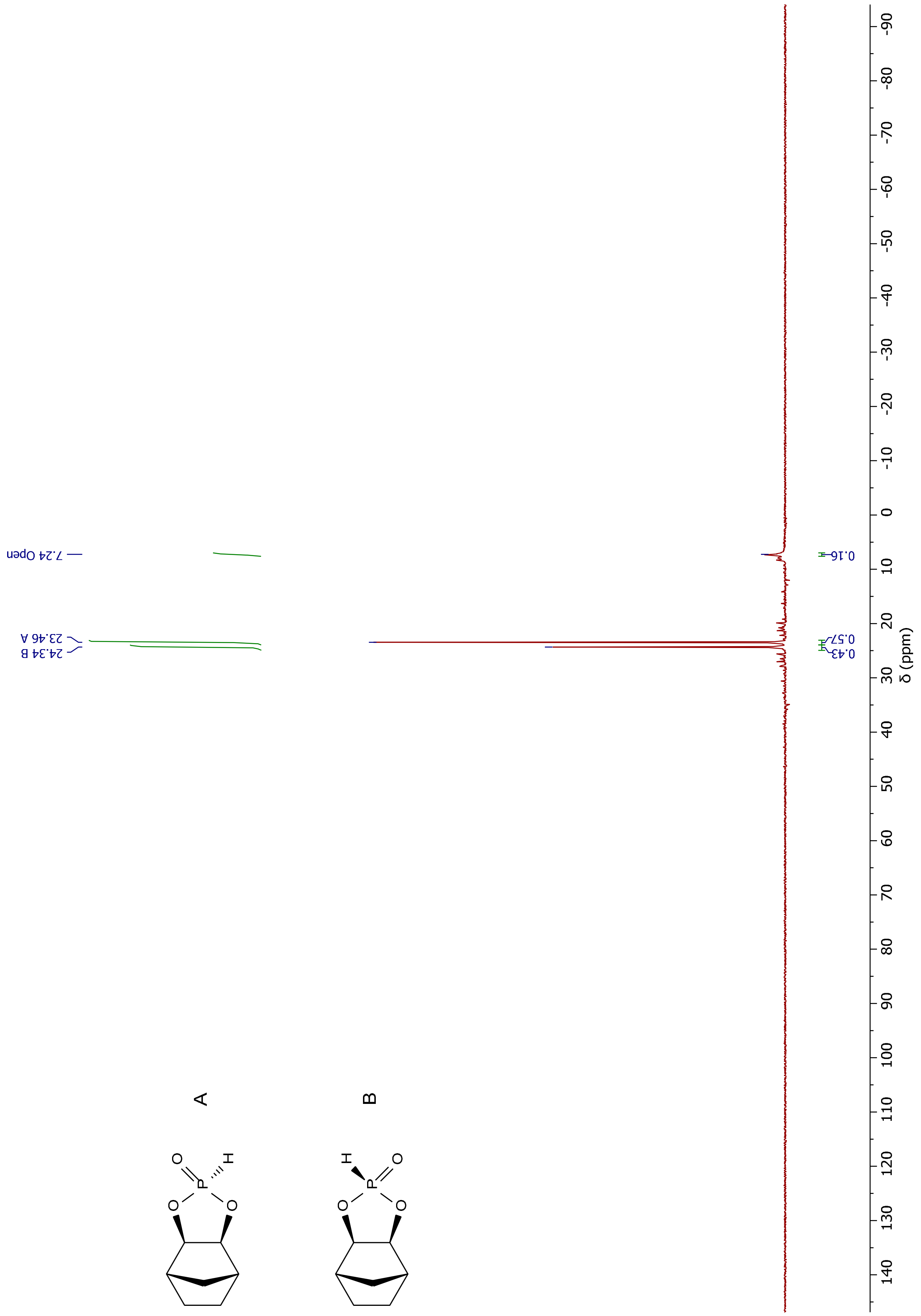

^{1}H-NMR spectra of 3

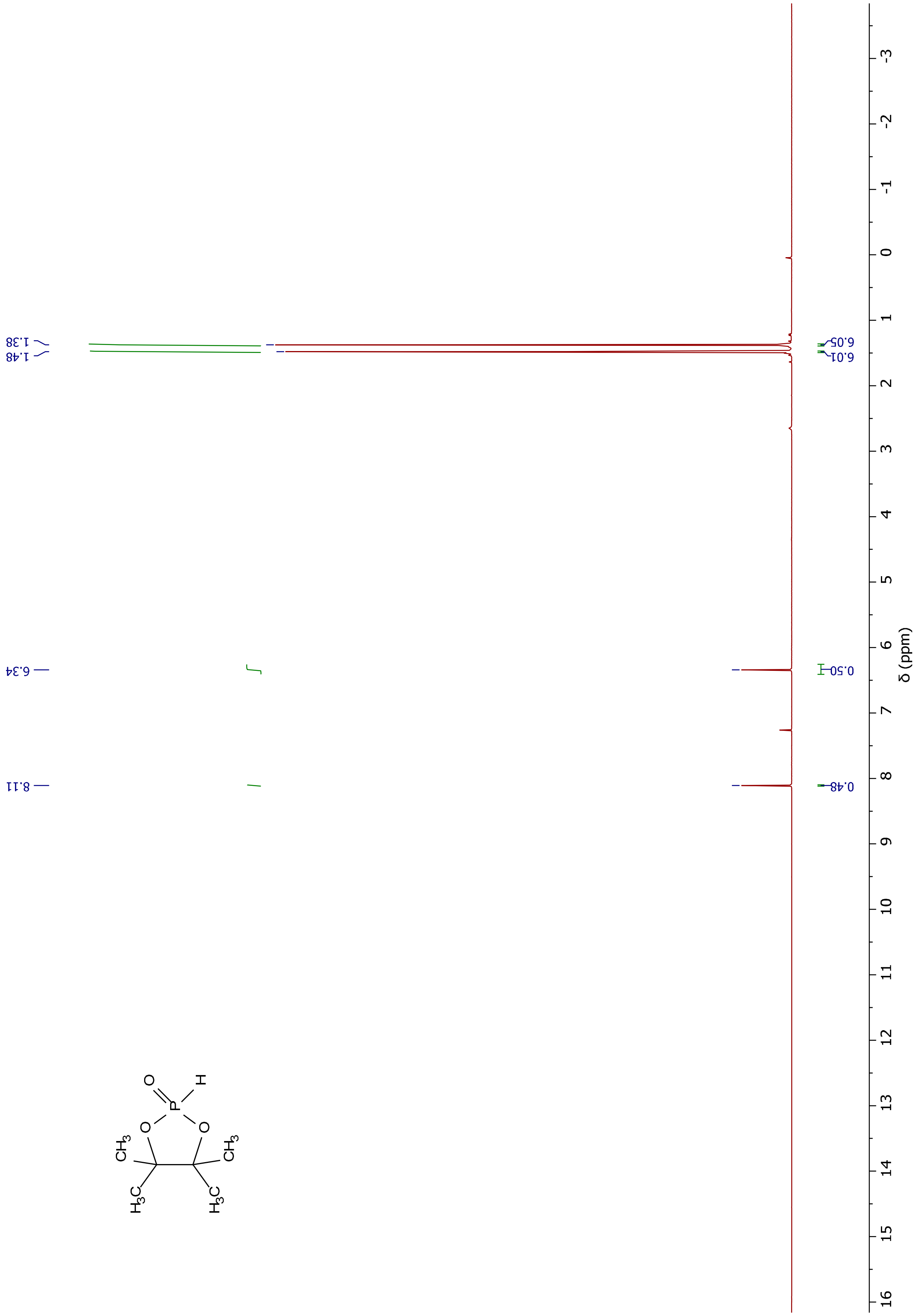

$^{13}C\{^{1}H\}$-NMR spectra of 3

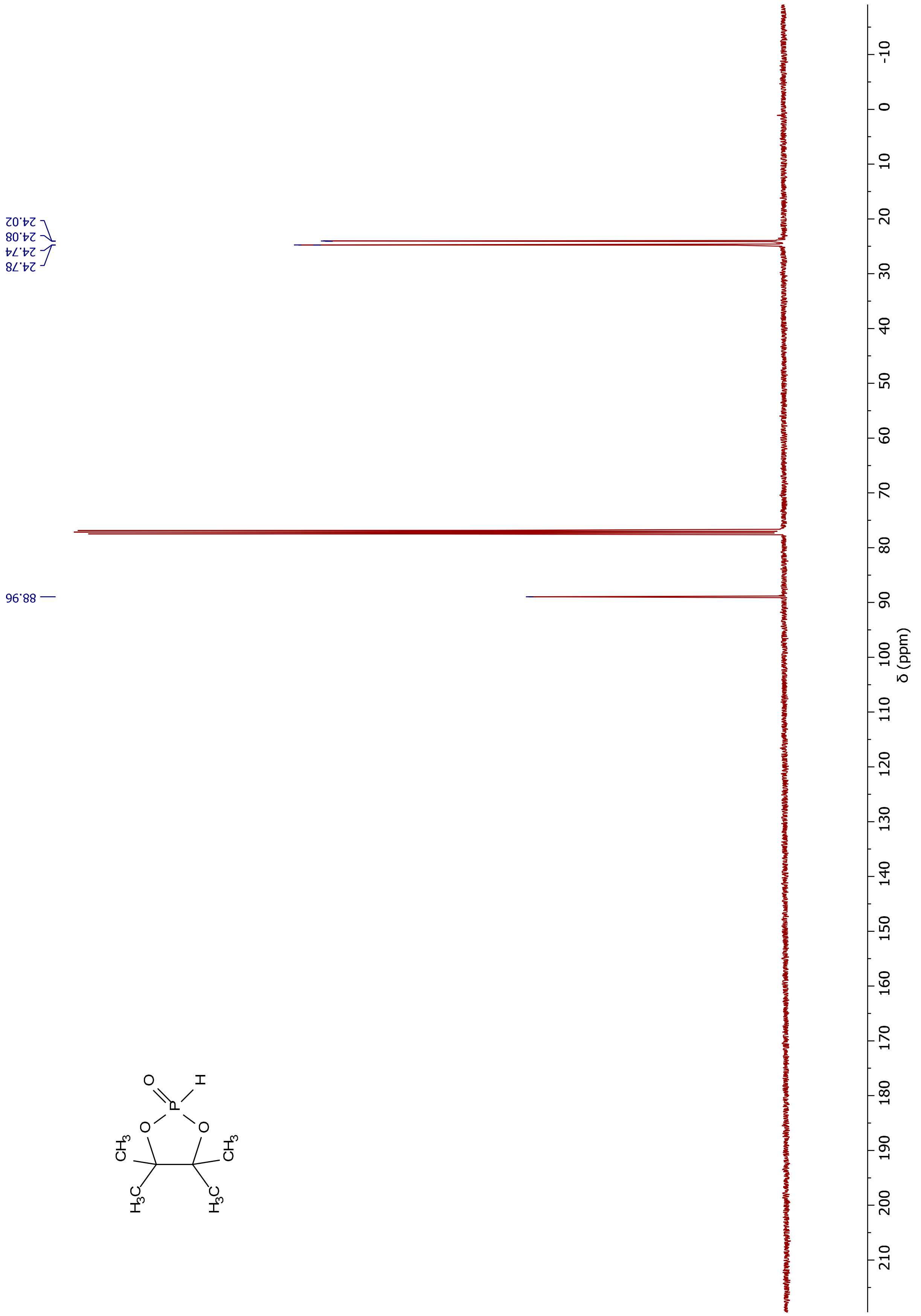

$^{31}P\{^1H\}$-NMR spectra of 3

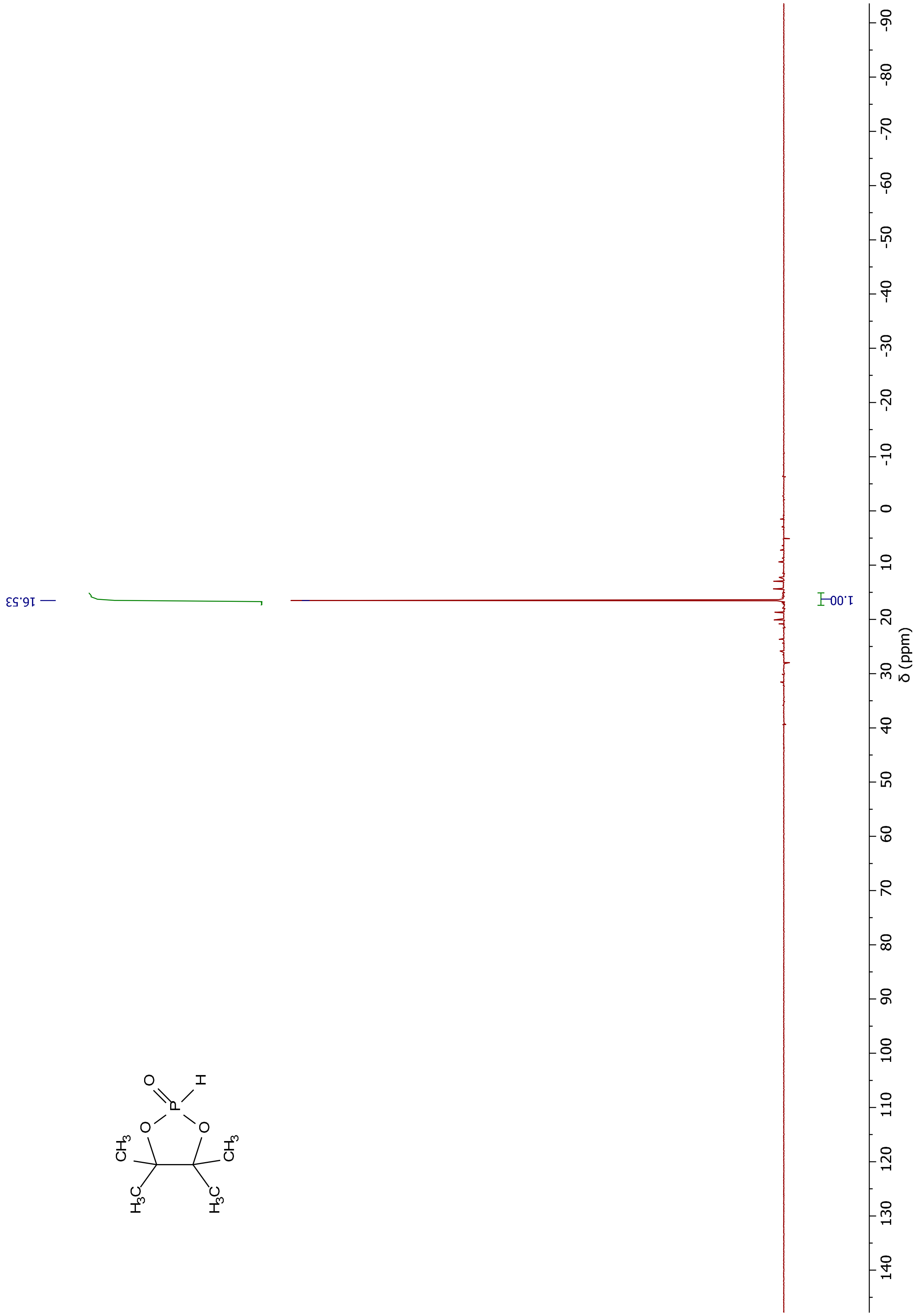

^{1}H-NMR spectra of 4

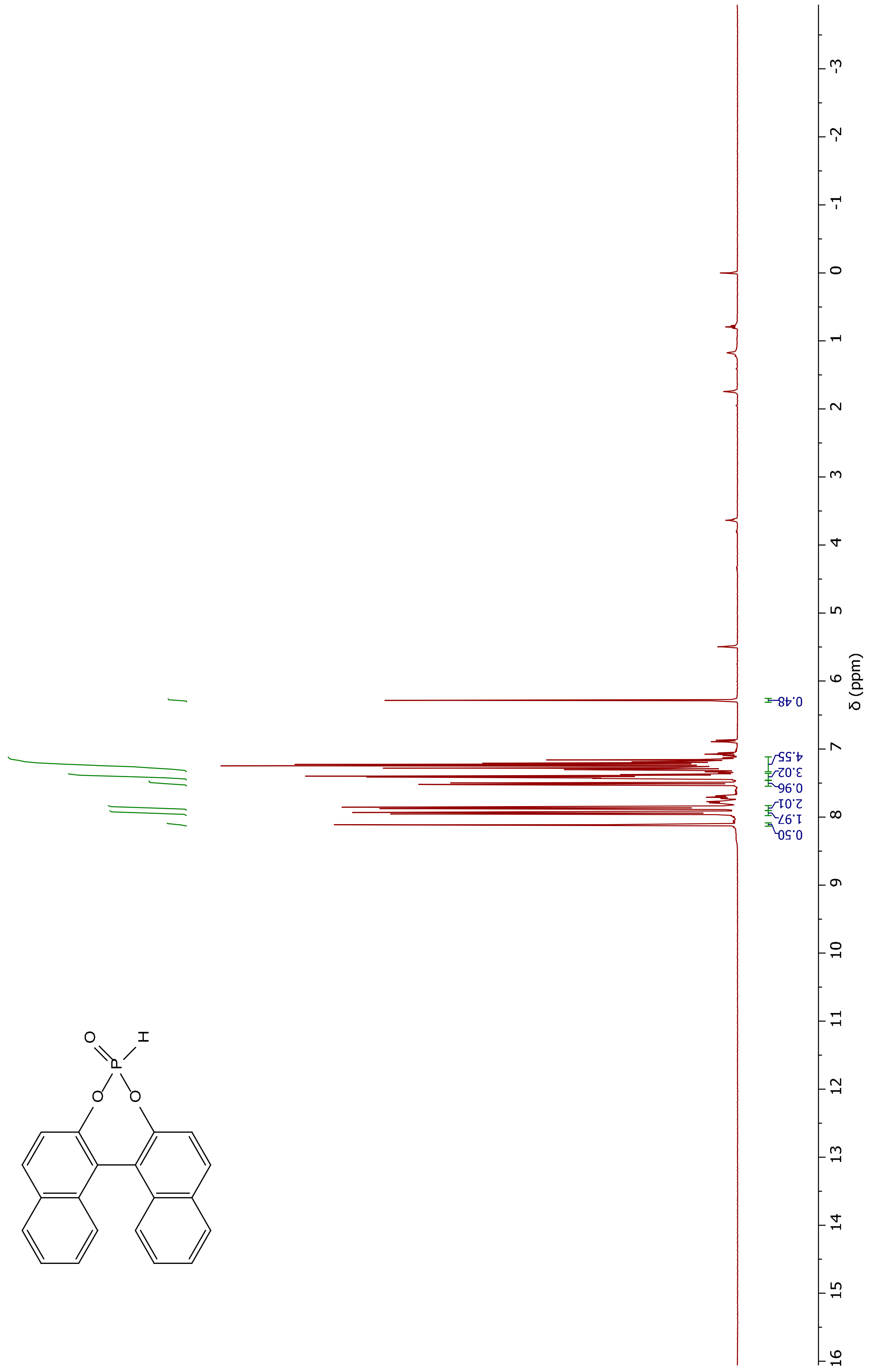

$^{13}C\{^{1}H\}$-NMR spectra of 4

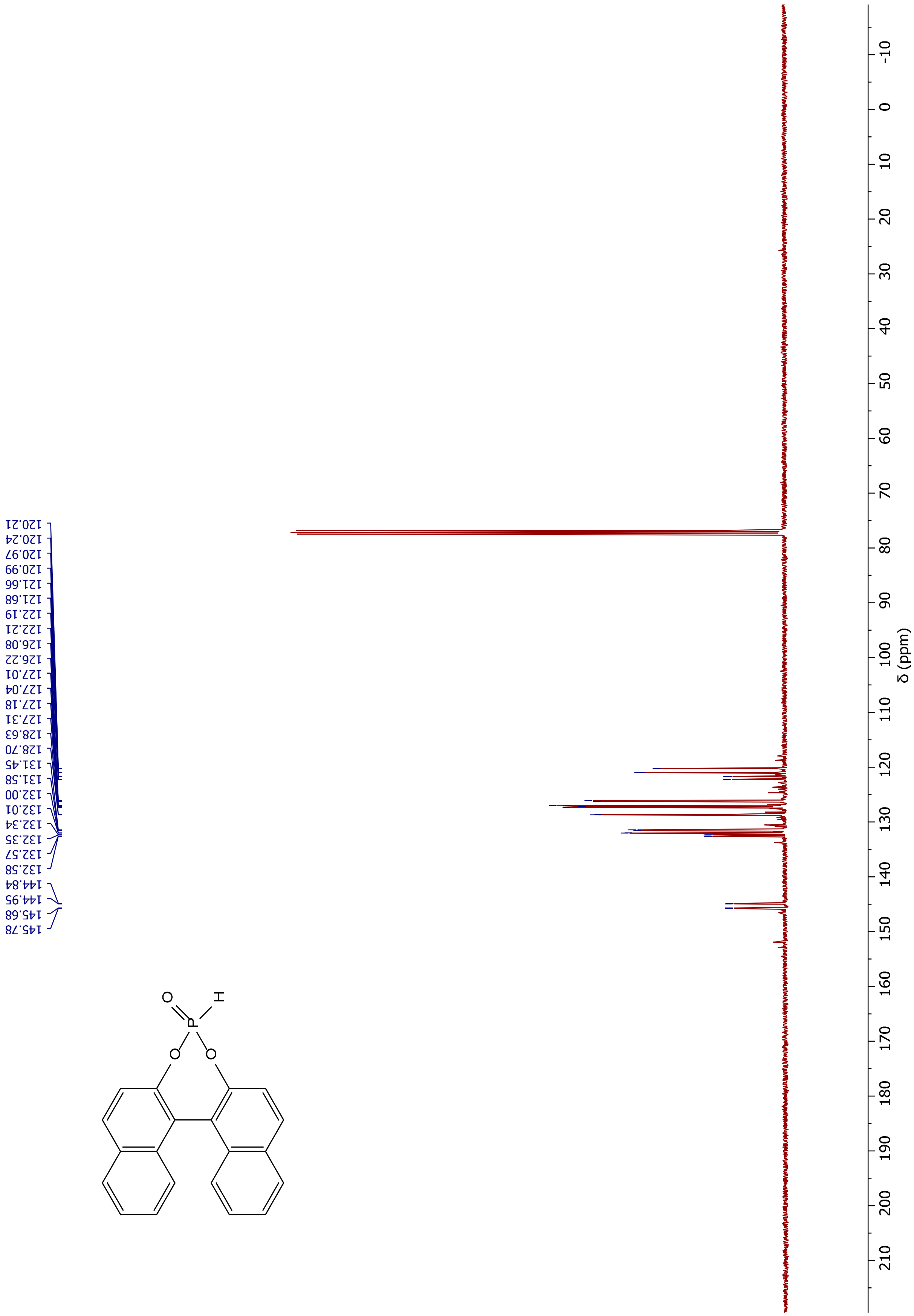

$^{31}P\{^{1}H\}$-NMR spectra of 4

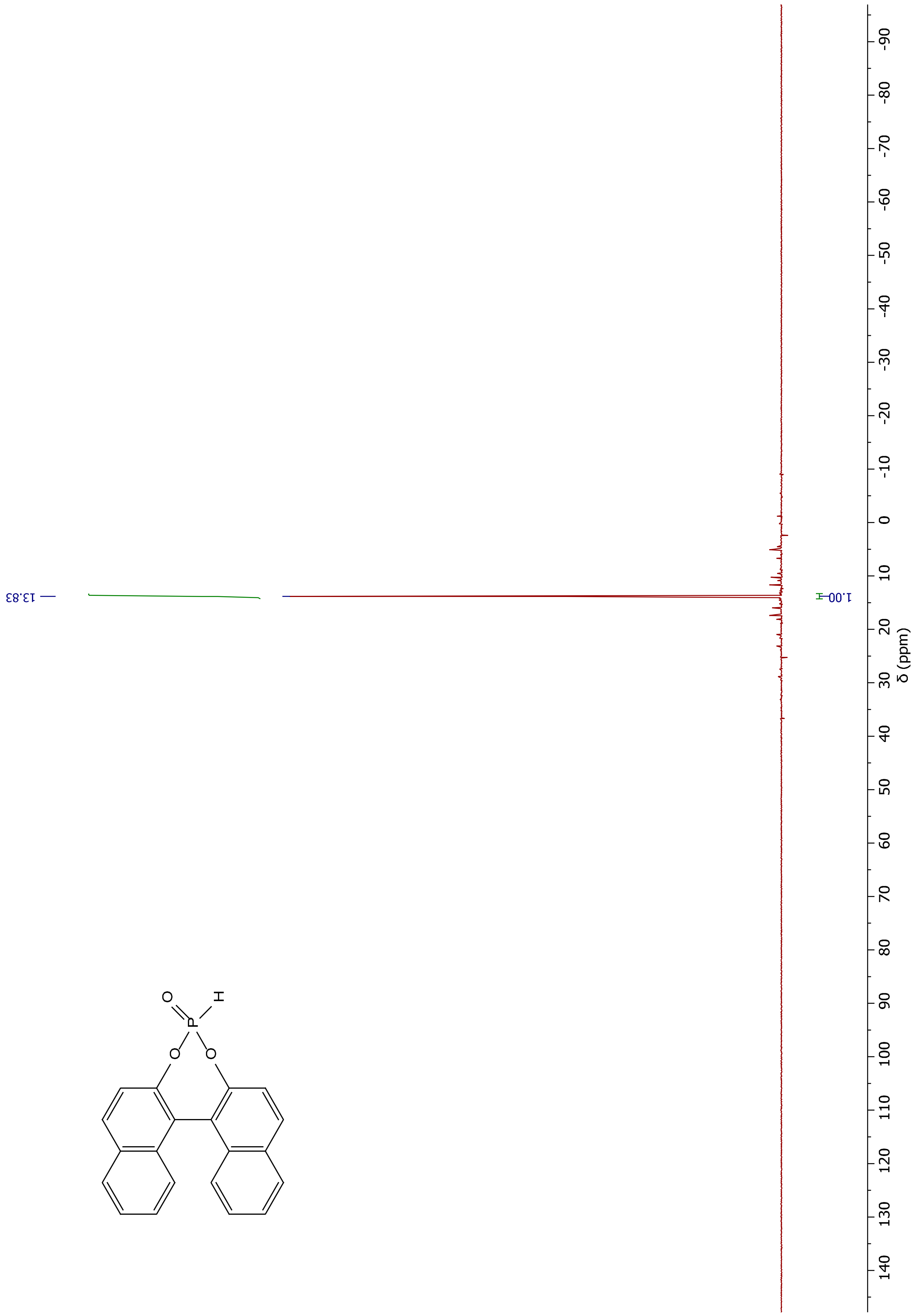

^{1}H-NMR spectra of 5

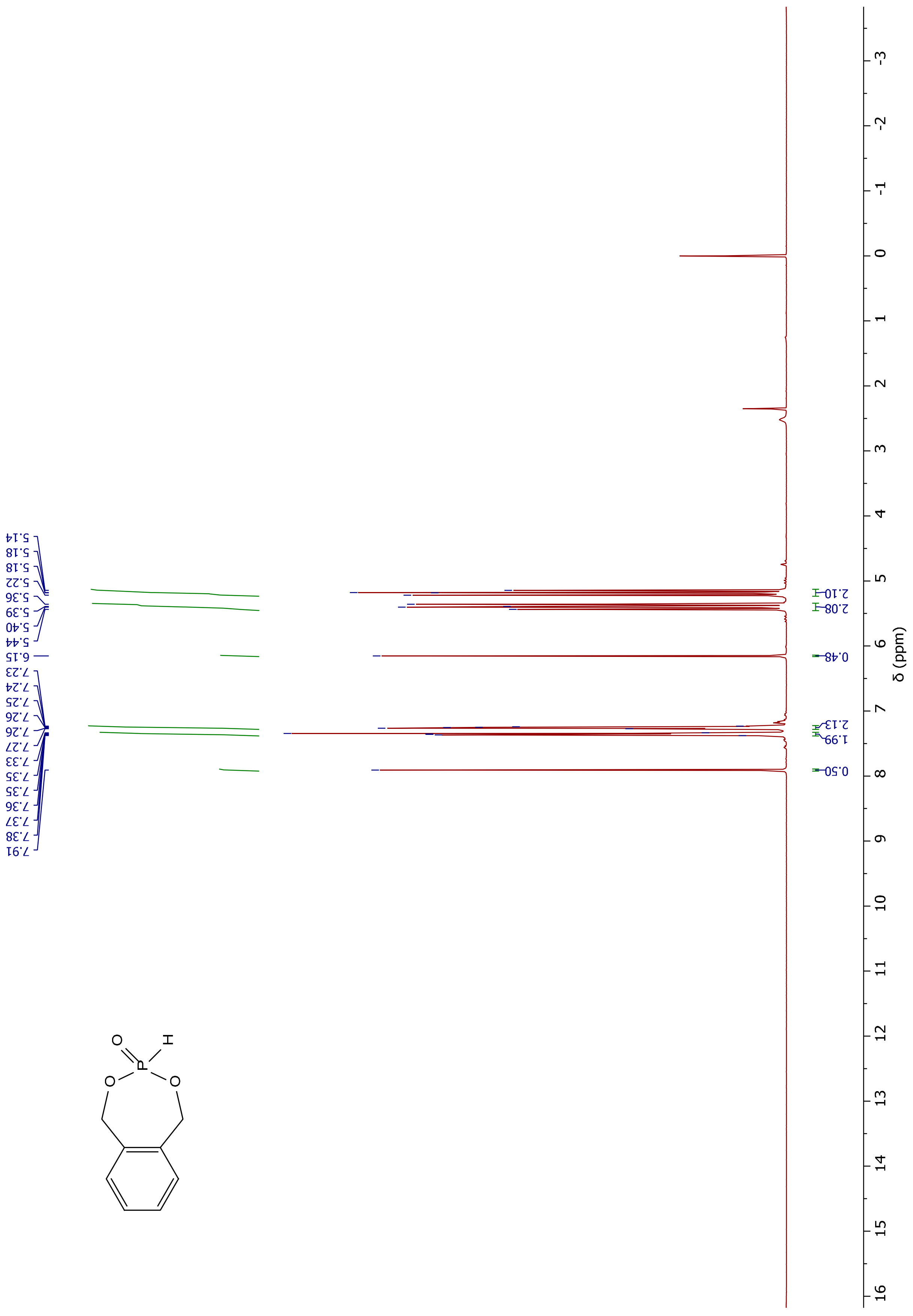
5.14
5.18
5.18
5.22
5.36
5.39
5.40
5.44
6.15
7.23
7.24
7.25
7.26
7.26
7.27
7.33
7.35
7.35
7.36
7.37
7.38
7.91
2.10
2.08
0.48
2.13
1.99
0.50
δ (ppm)
O
P
H
O
O

$^{13}C\{^1H\}$-NMR spectra of 5

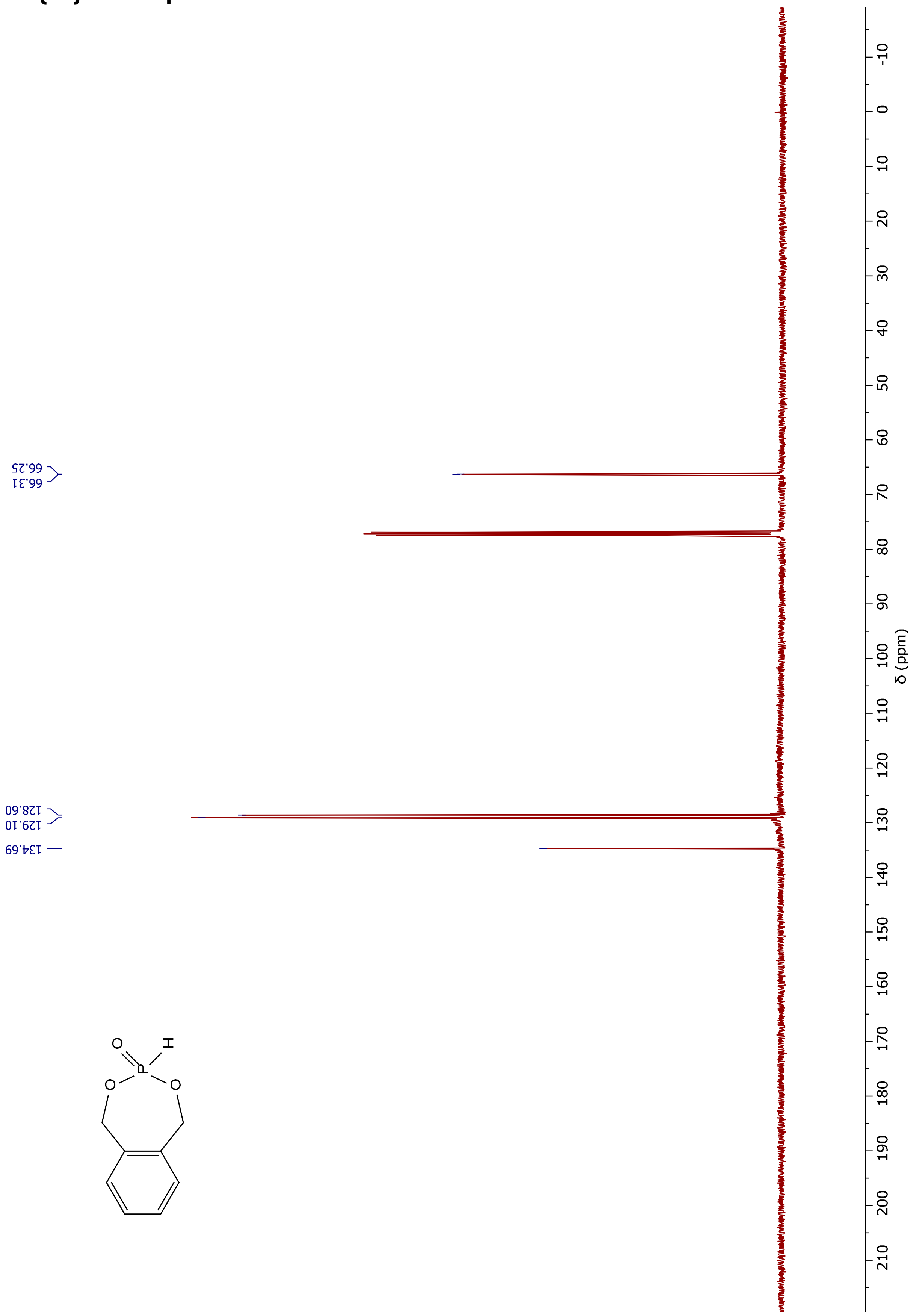

$^{31}P\{^{1}H\}$-NMR spectra of 5

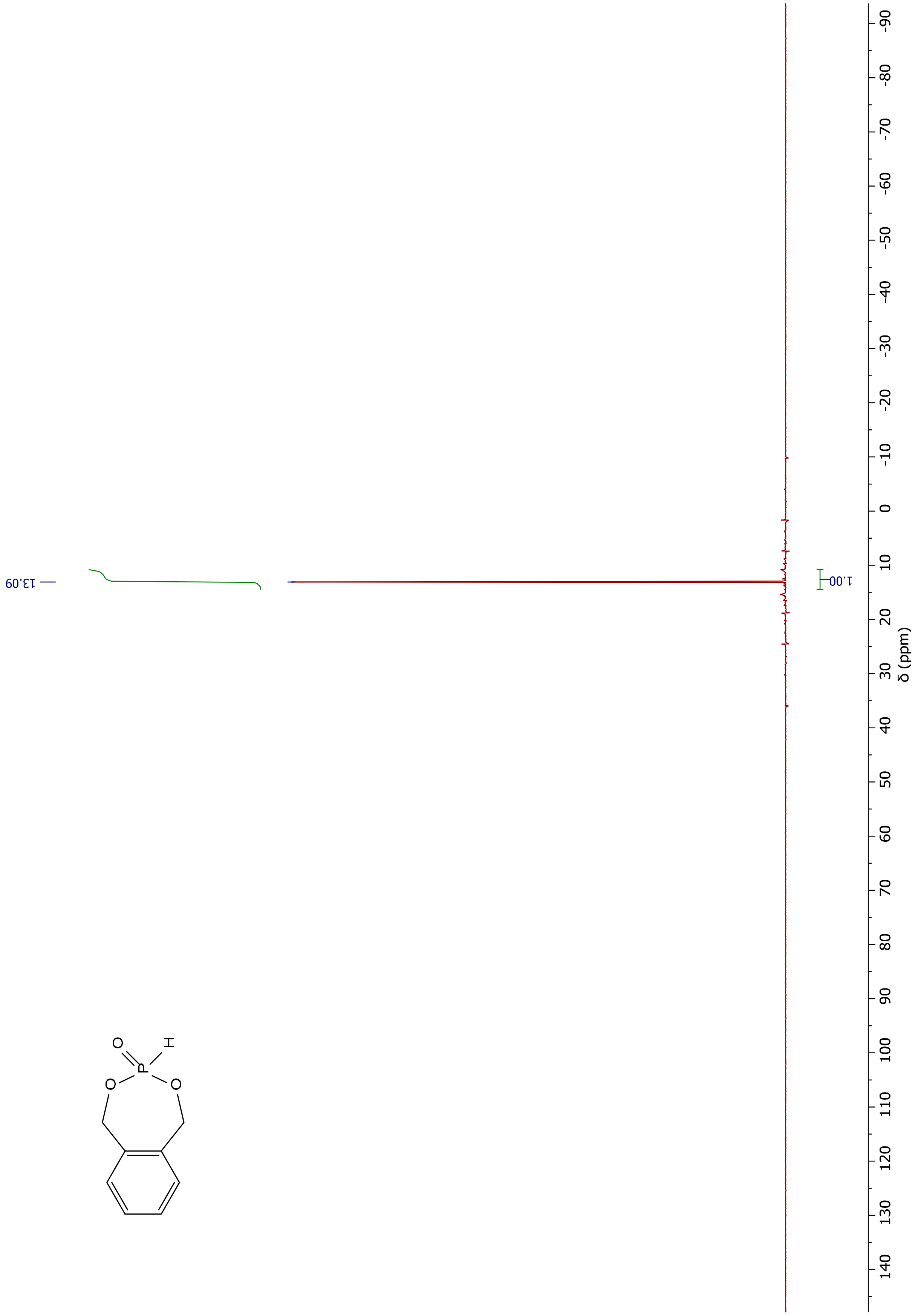

^{1}H-NMR spectra of 8

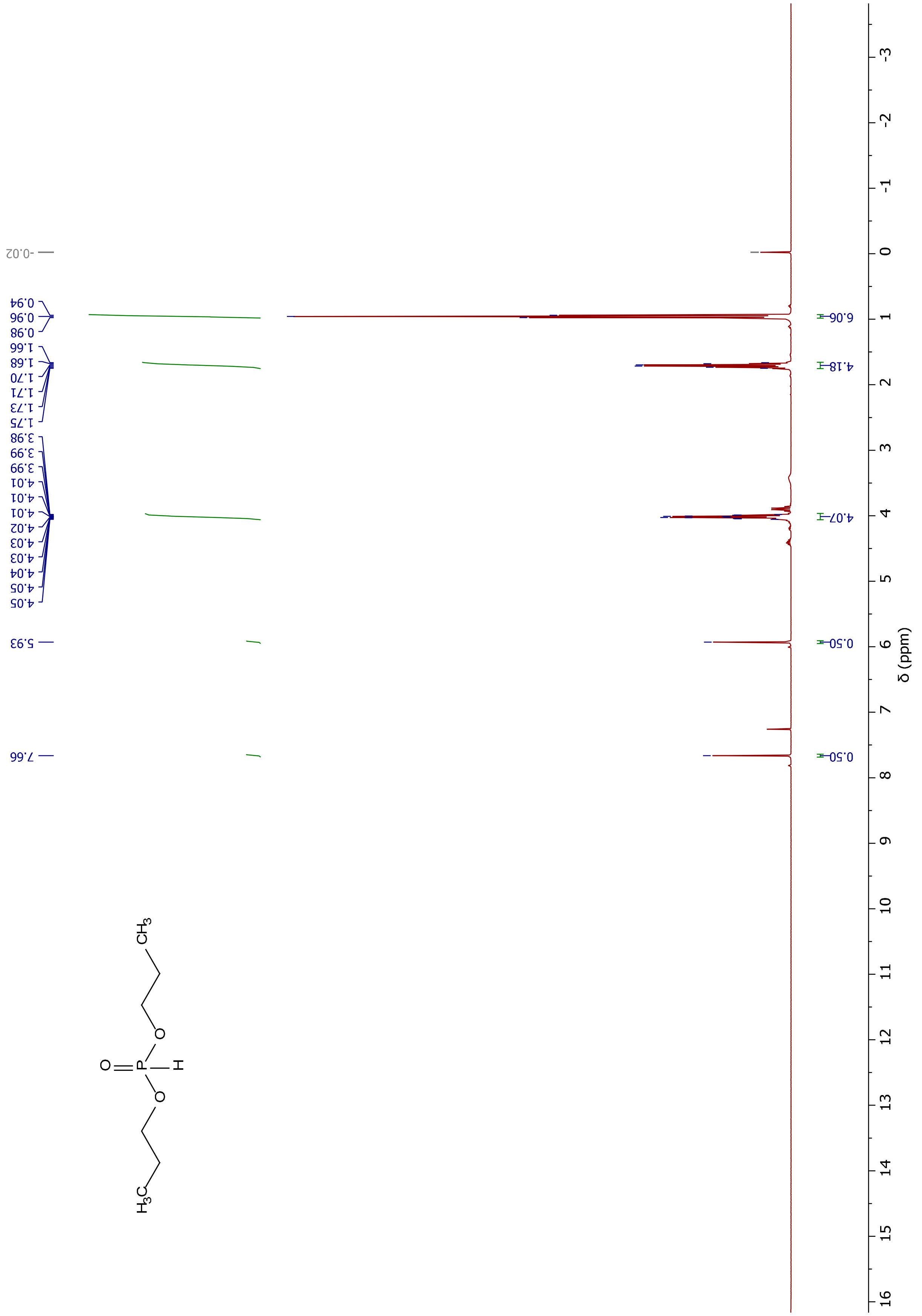

$^{13}C\{^{1}H\}$-NMR spectra of 8

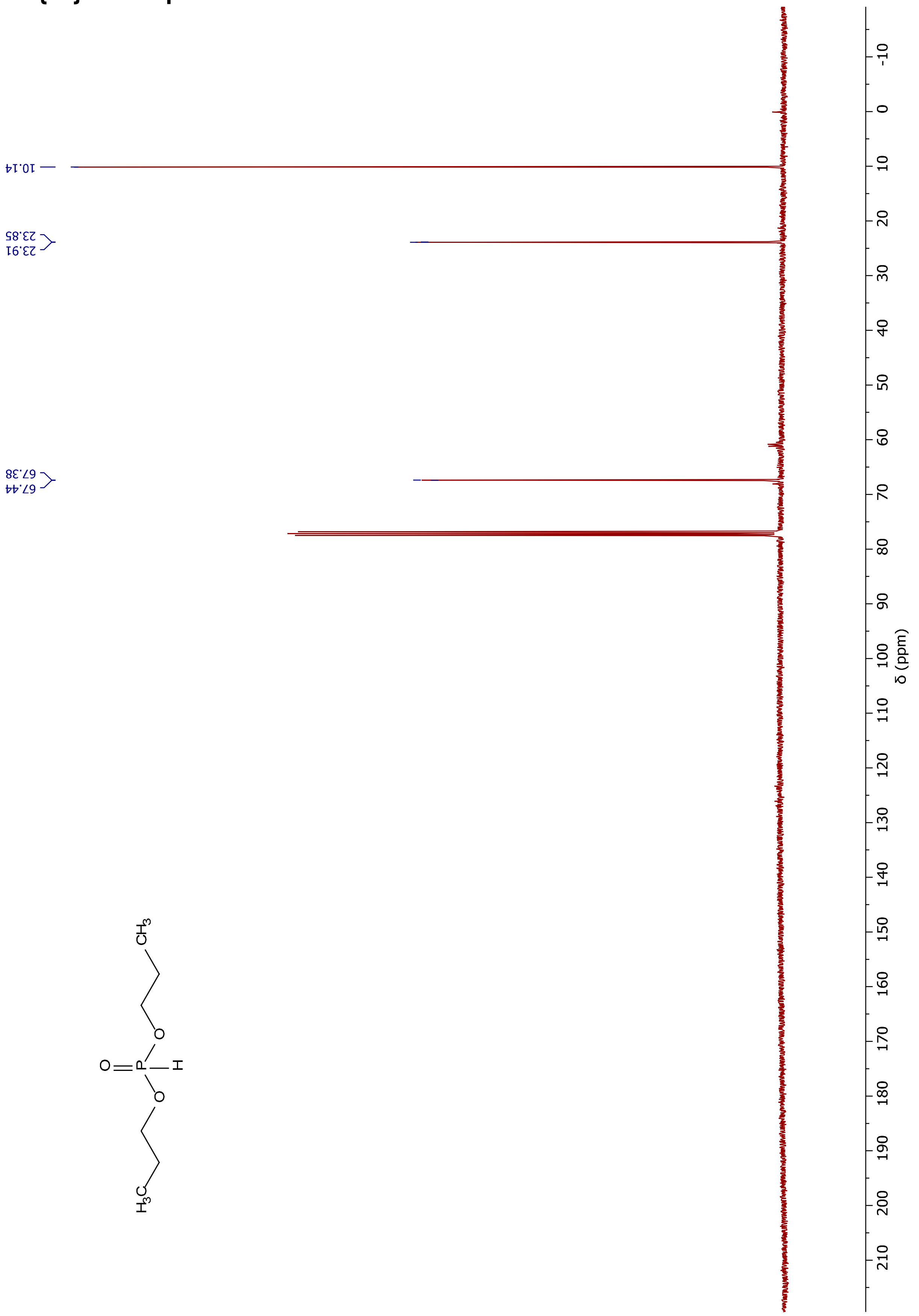

$^{31}P\{^1H\}$-NMR spectra of 8

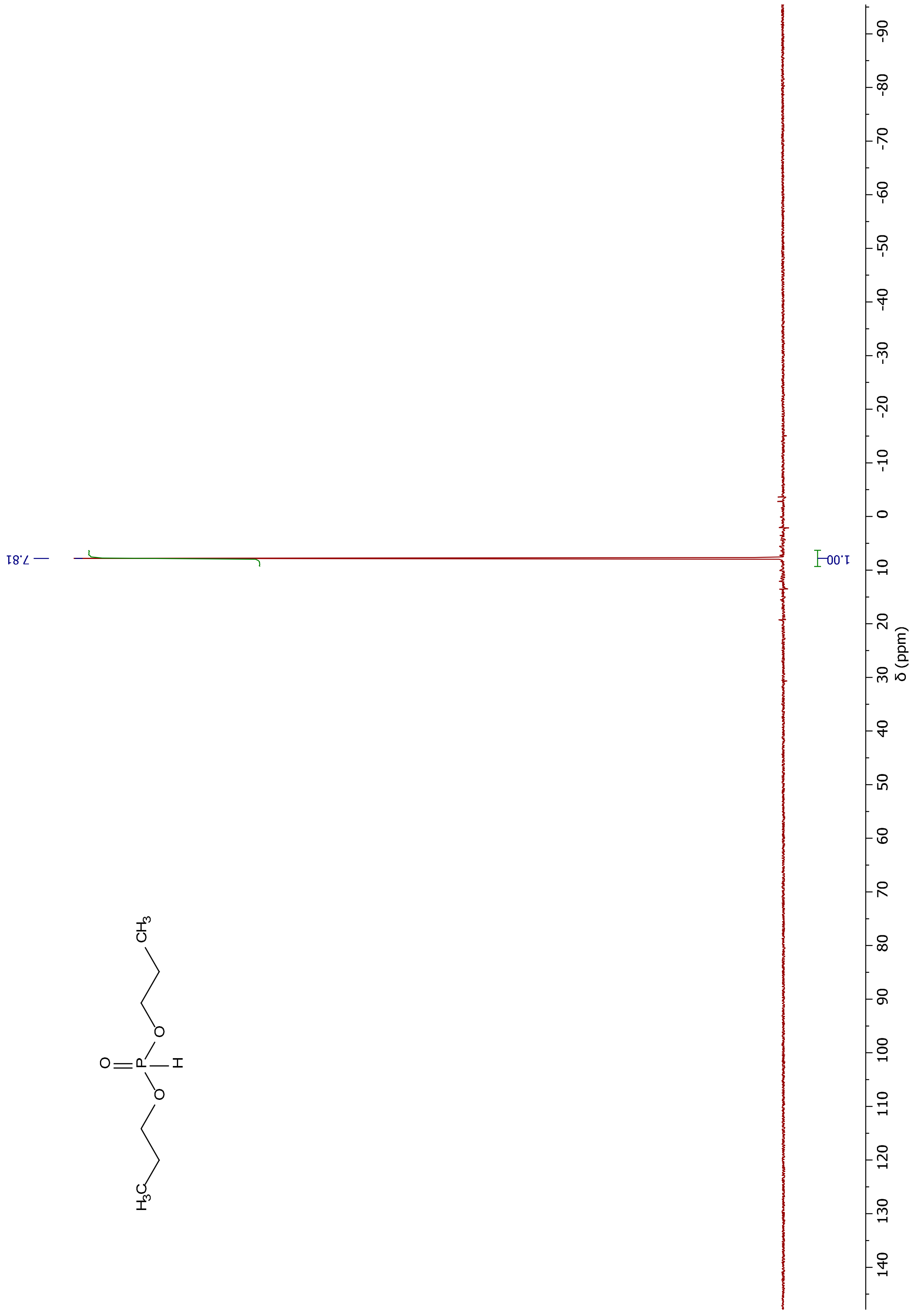

^{1}H-NMR spectra of 9

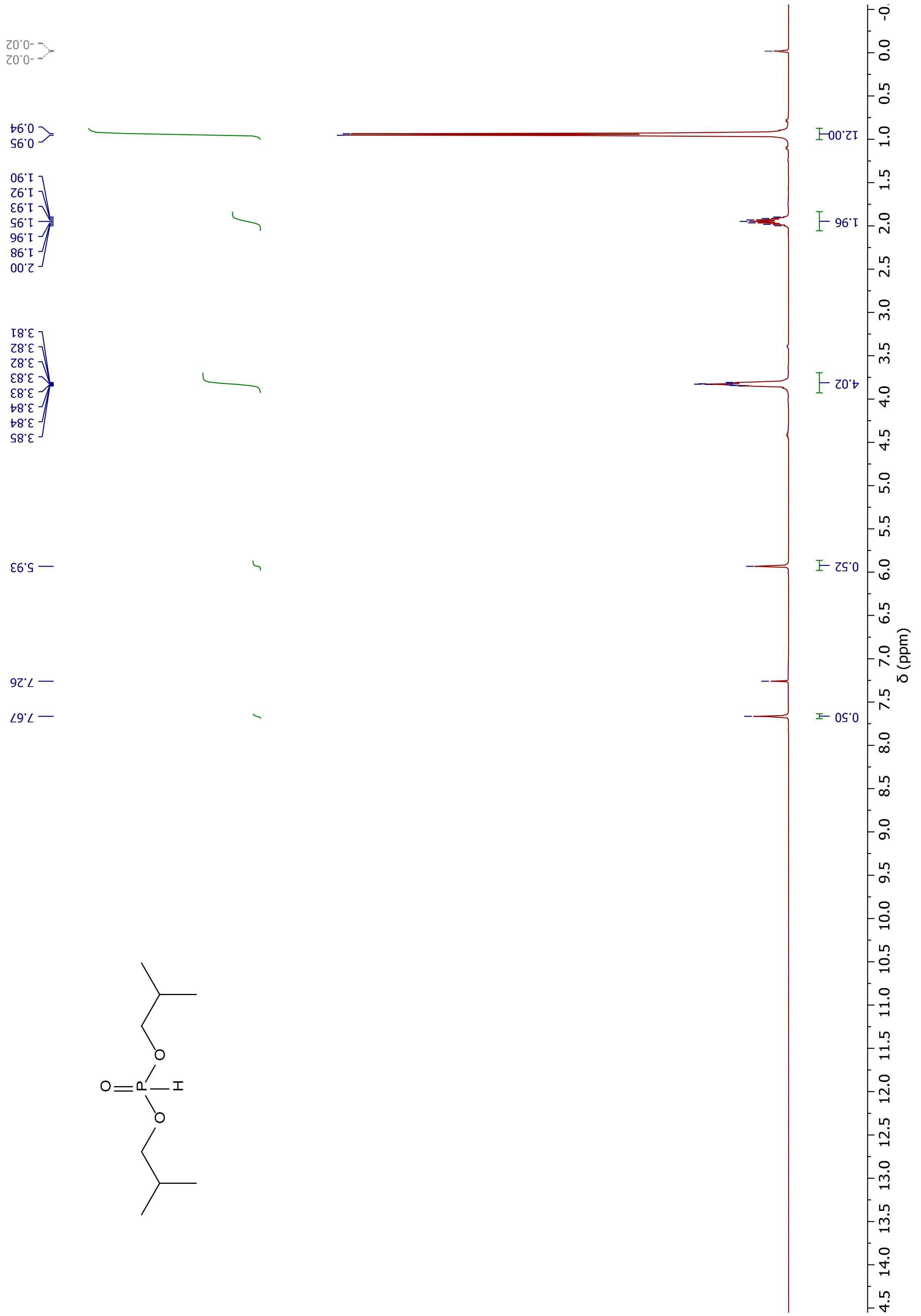

$^{13}C\{^{1}H\}$-NMR spectra of 9

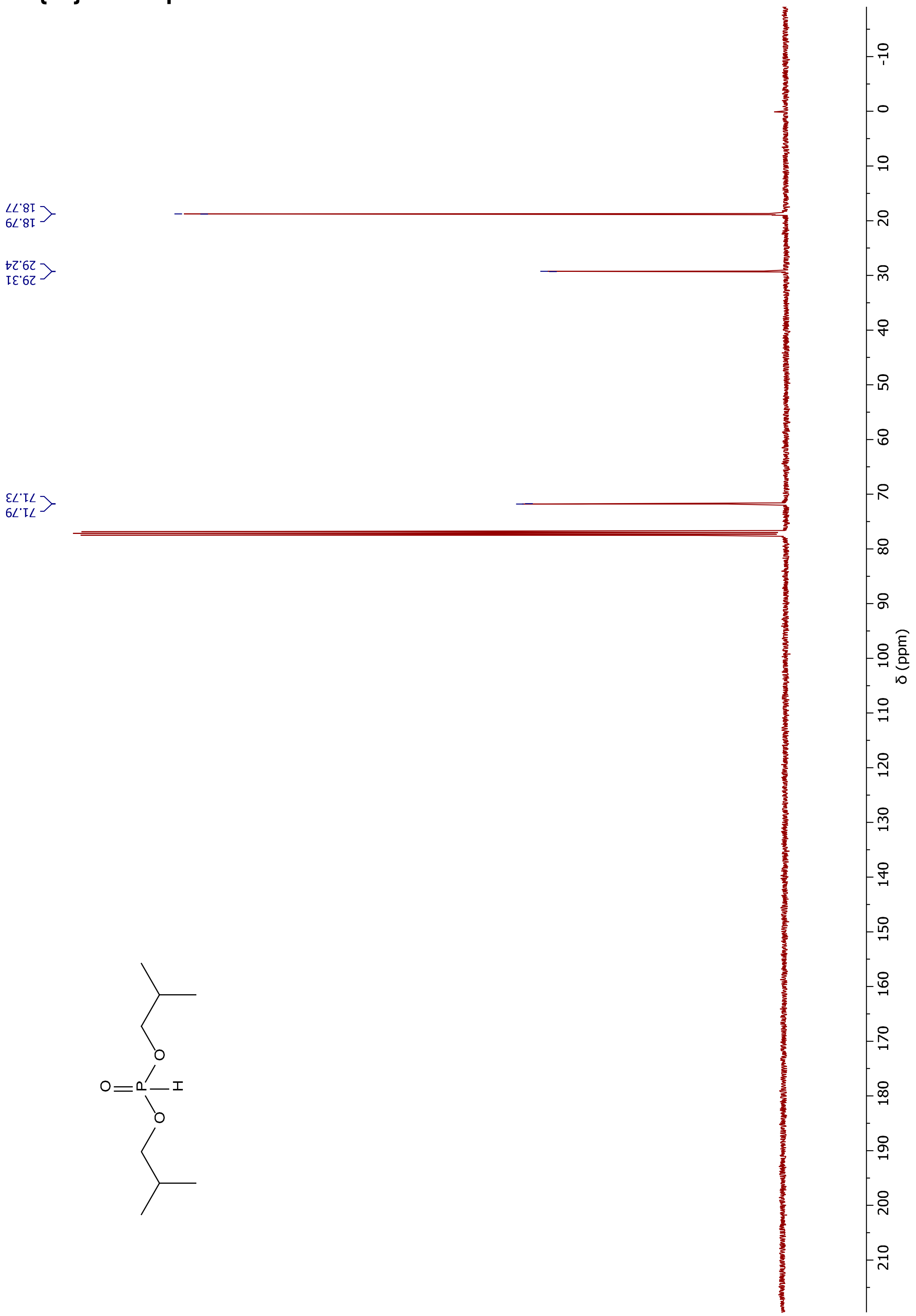

$^{31}P\{^{1}H\}$-NMR spectra of 9

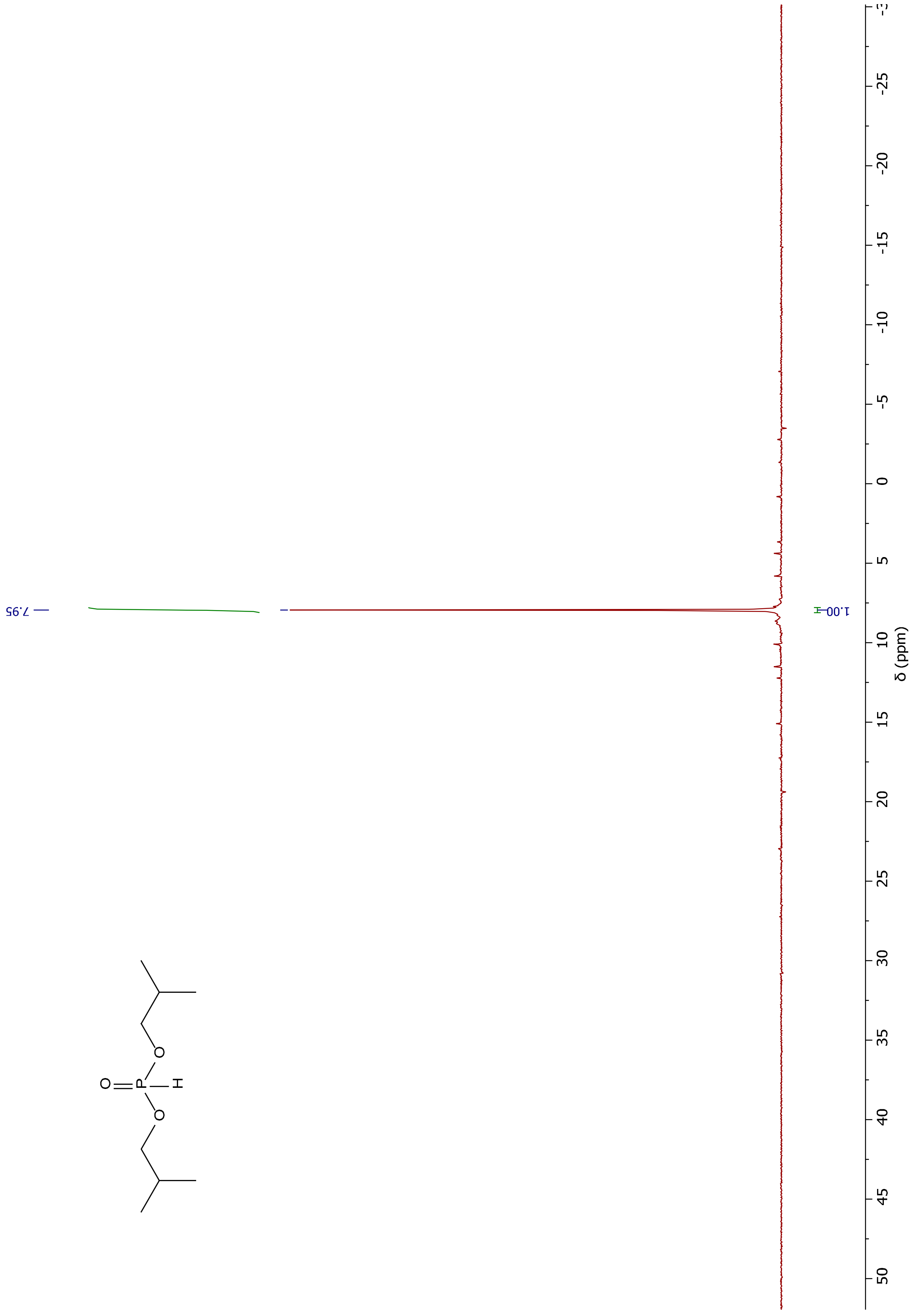

^{1}H-NMR spectra of 10

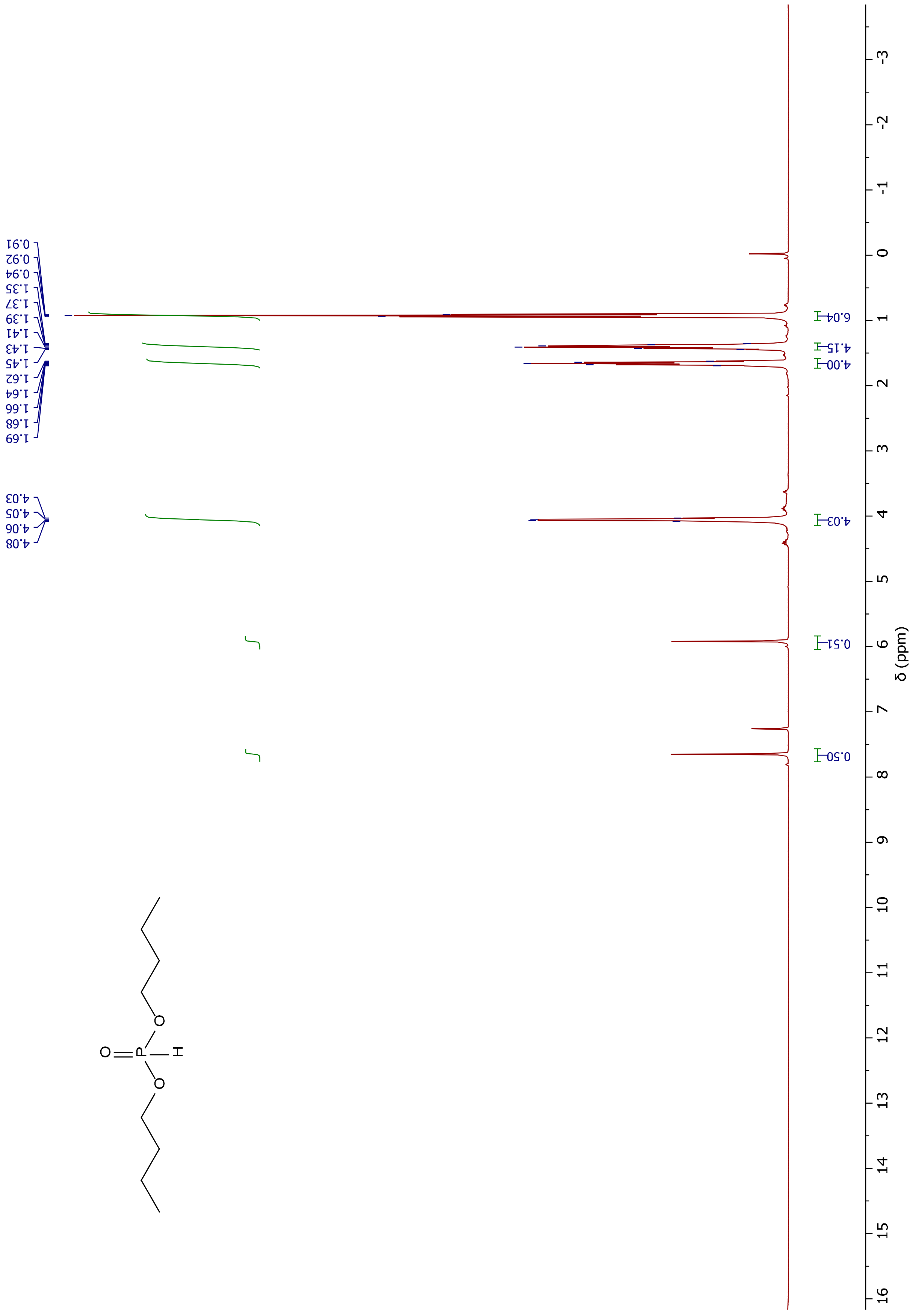

$^{13}C\{^{1}H\}$-NMR spectra of 10

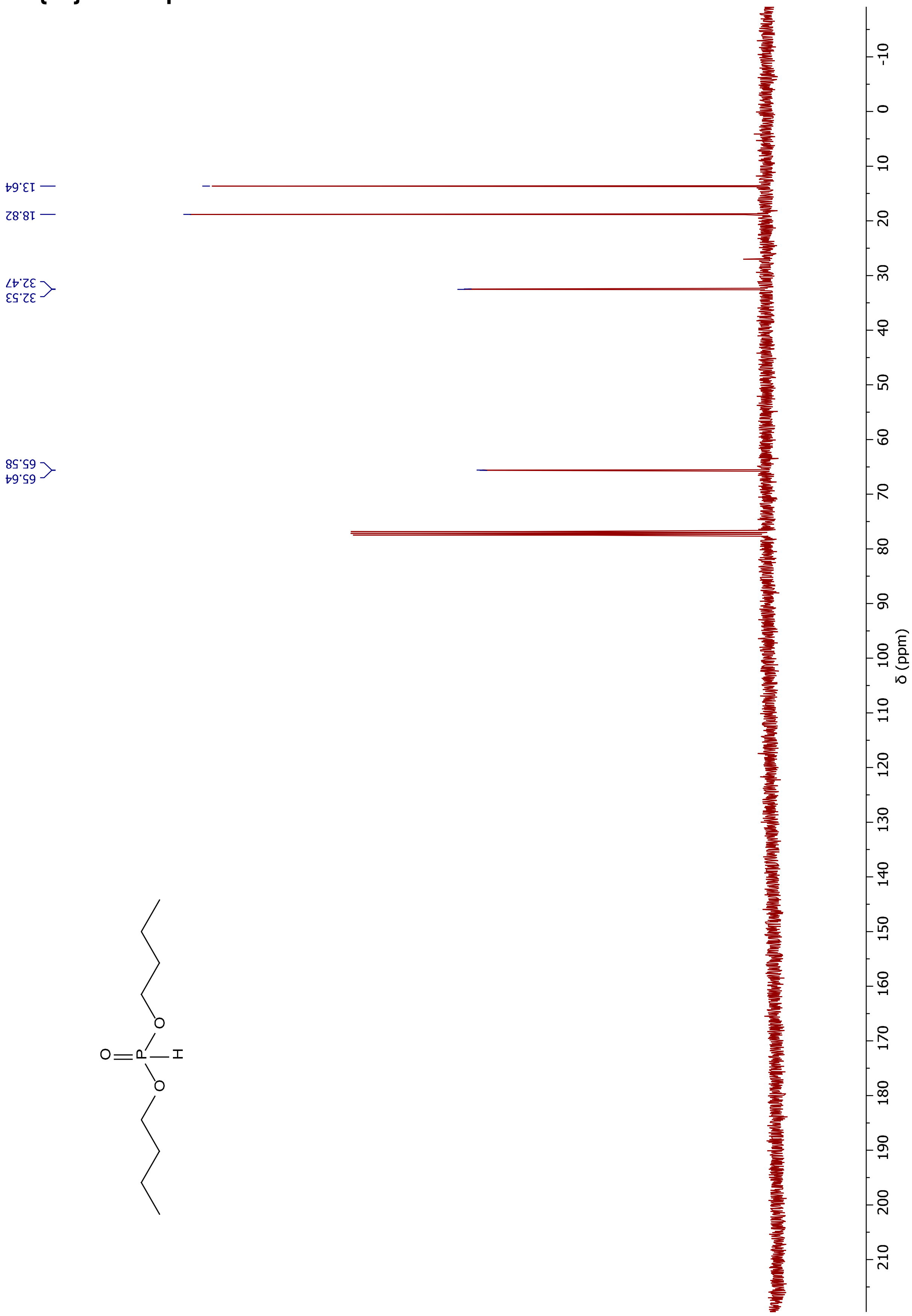

$^{31}P\{^{1}H\}$-NMR spectra of 10

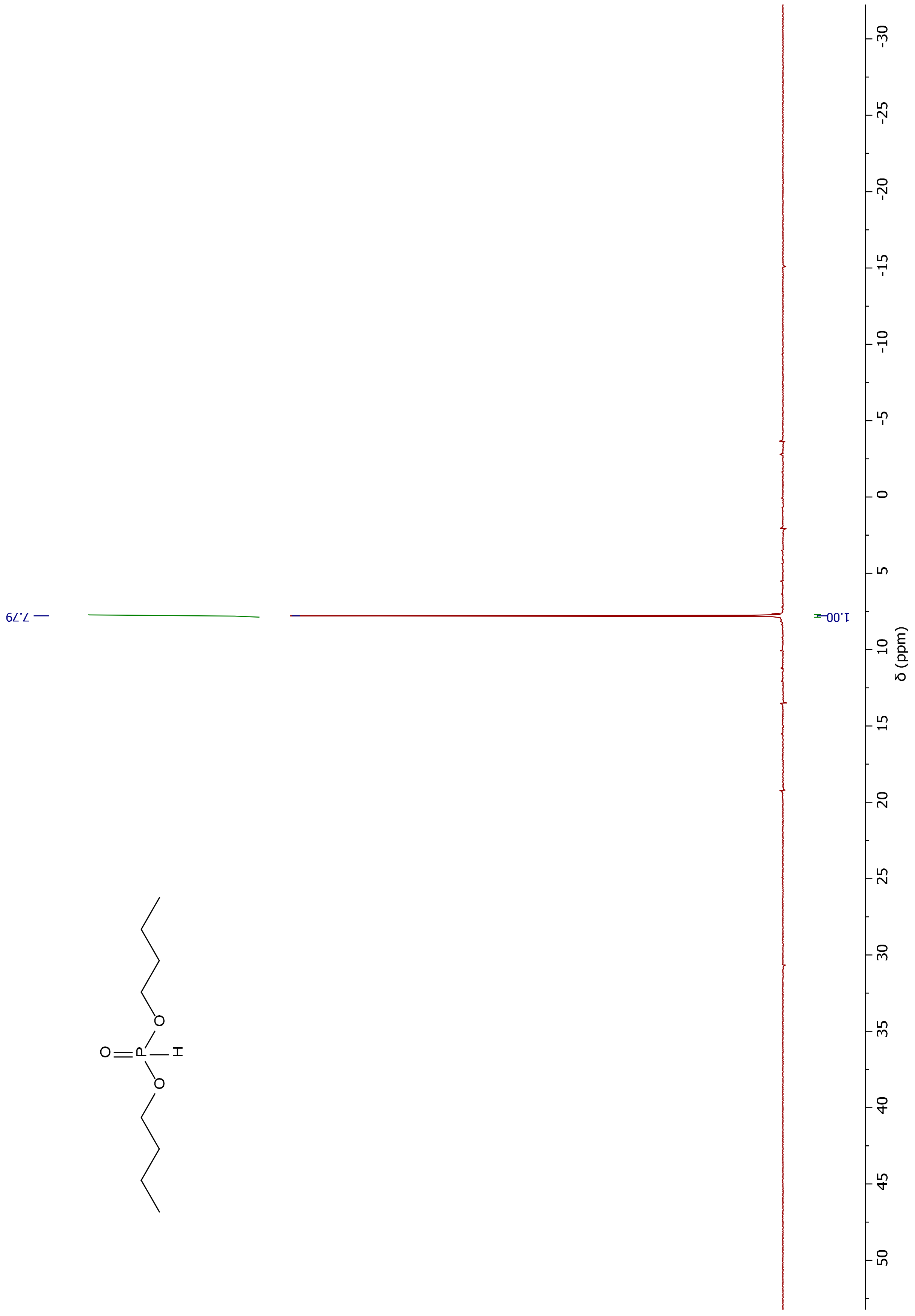

^{1}H-NMR spectra of 11

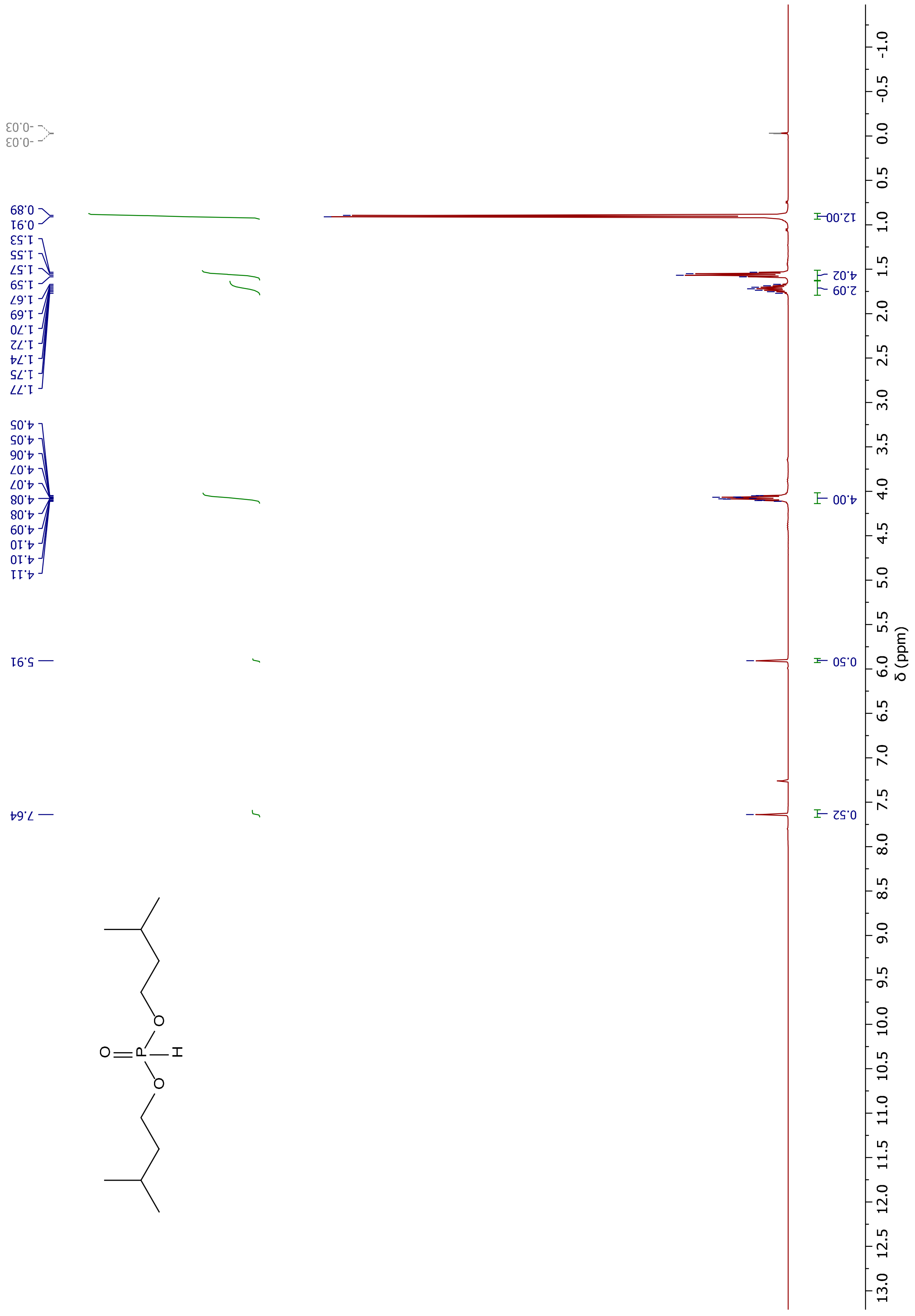

$^{13}C\{^1H\}$-NMR spectra of 11

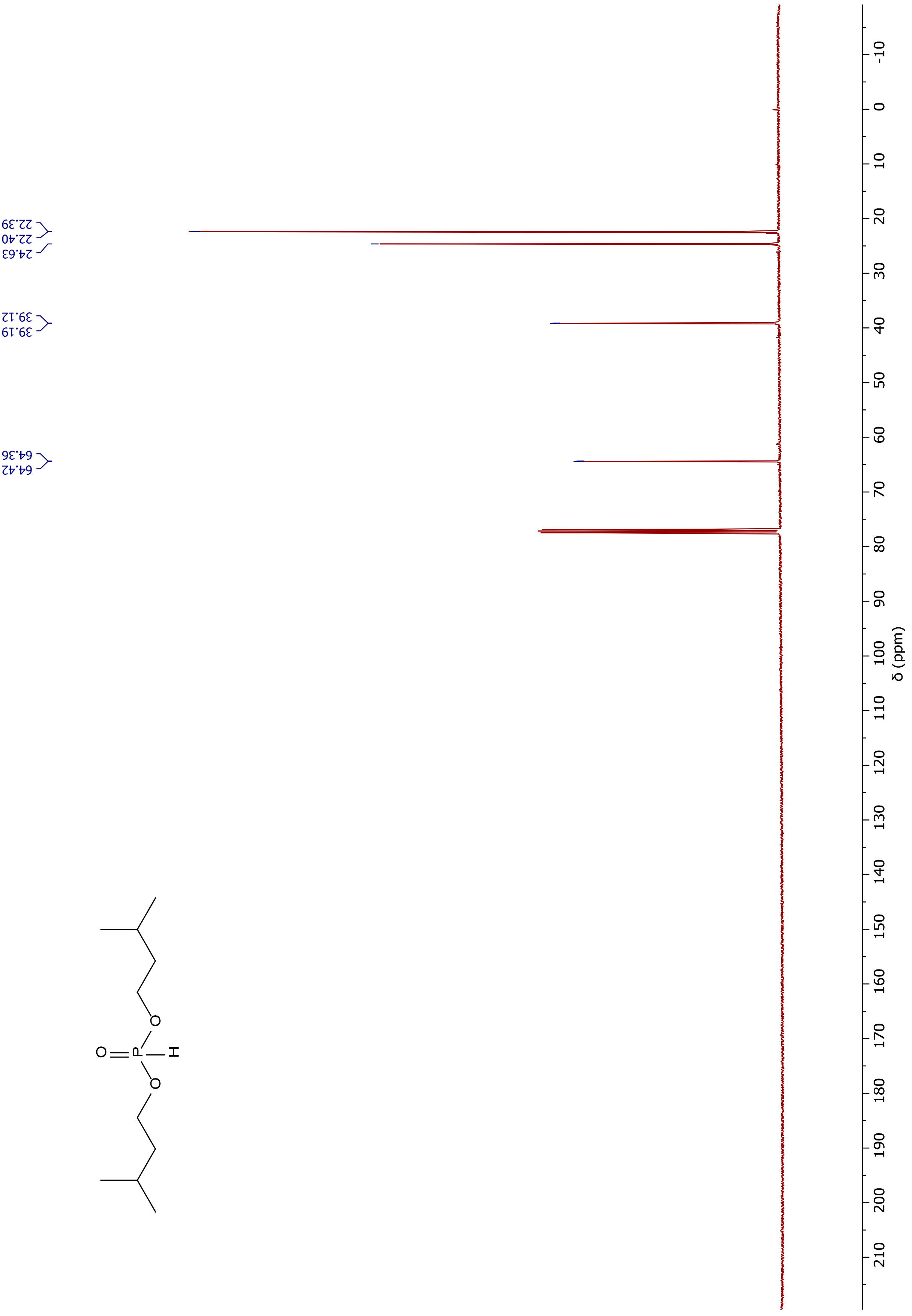

$^{31}P\{^{1}H\}$-NMR spectra of 11

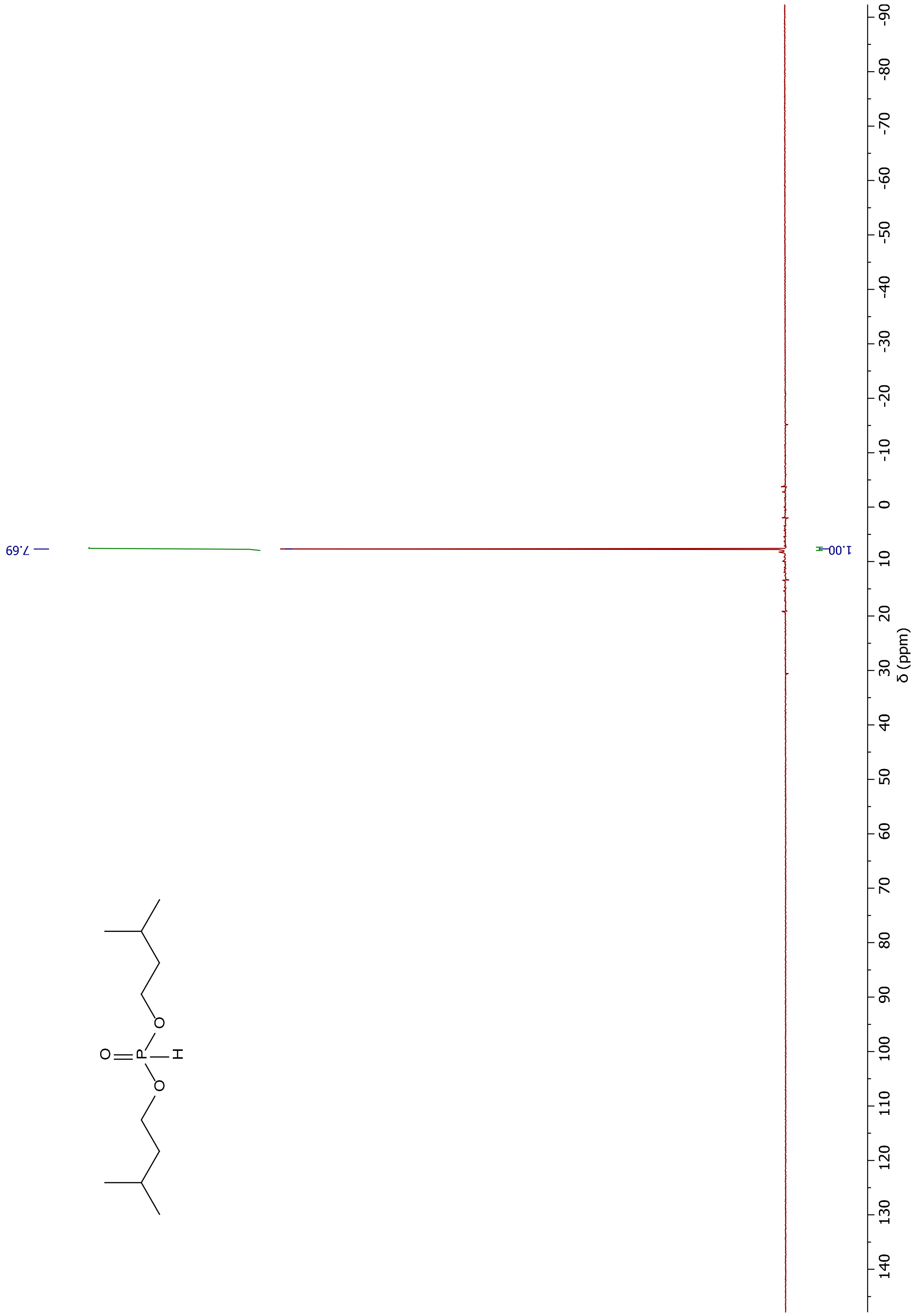

^{1}H-NMR spectra of 12

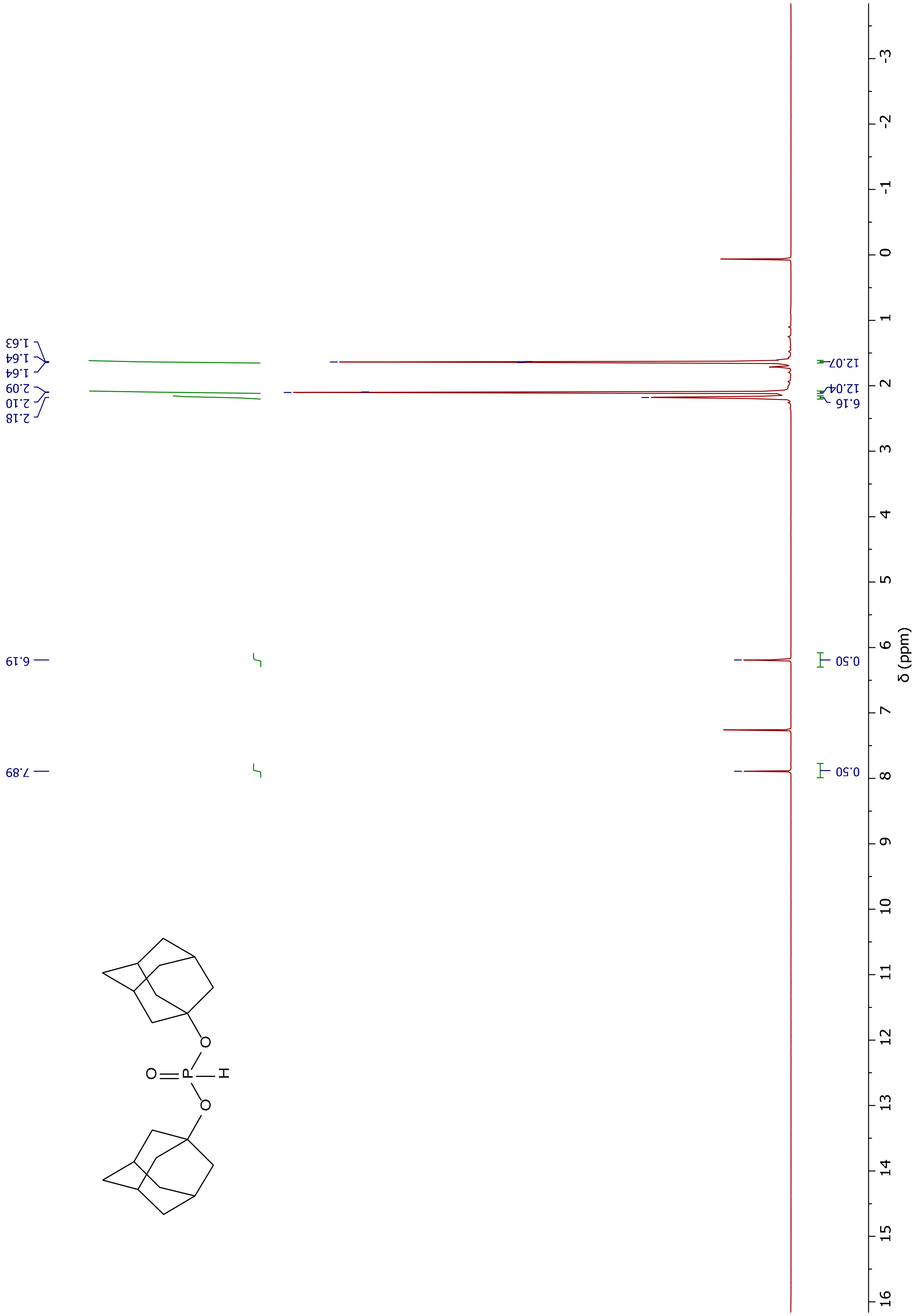

$^{13}C\{^1H\}$-NMR spectra of 12

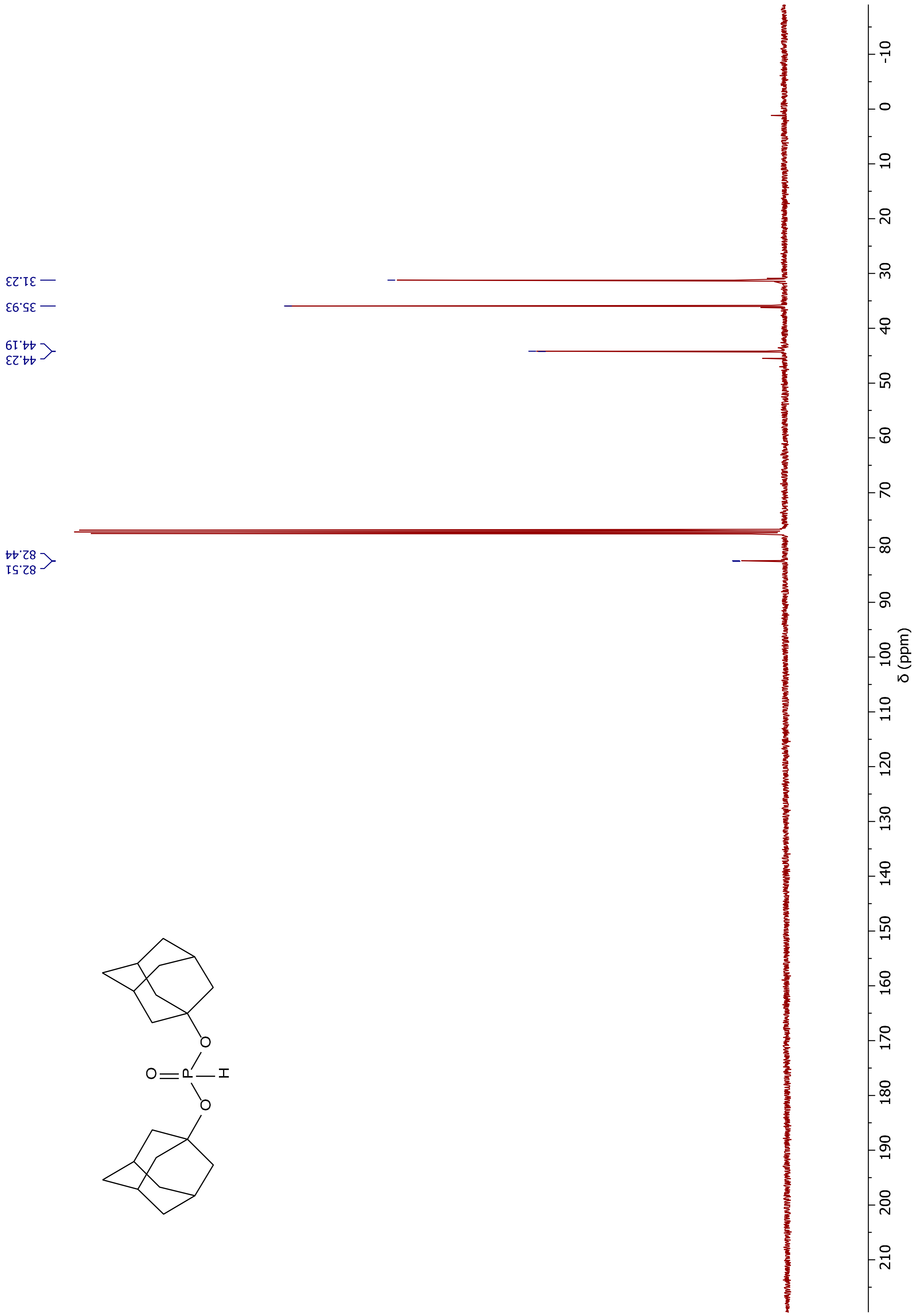

$^{31}P\{^{1}H\}$-NMR spectra of 12

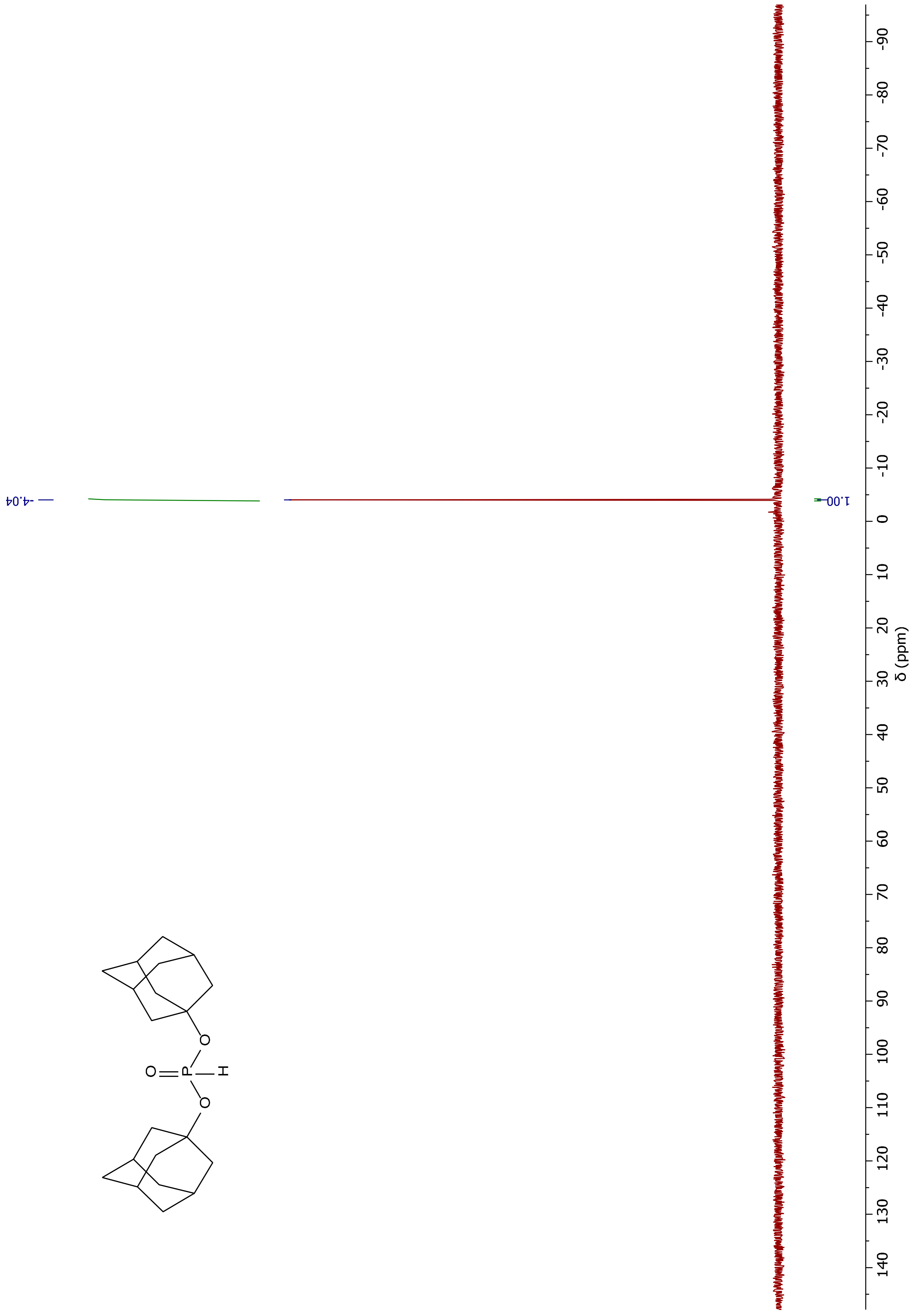

^{1}H-NMR spectra of 13

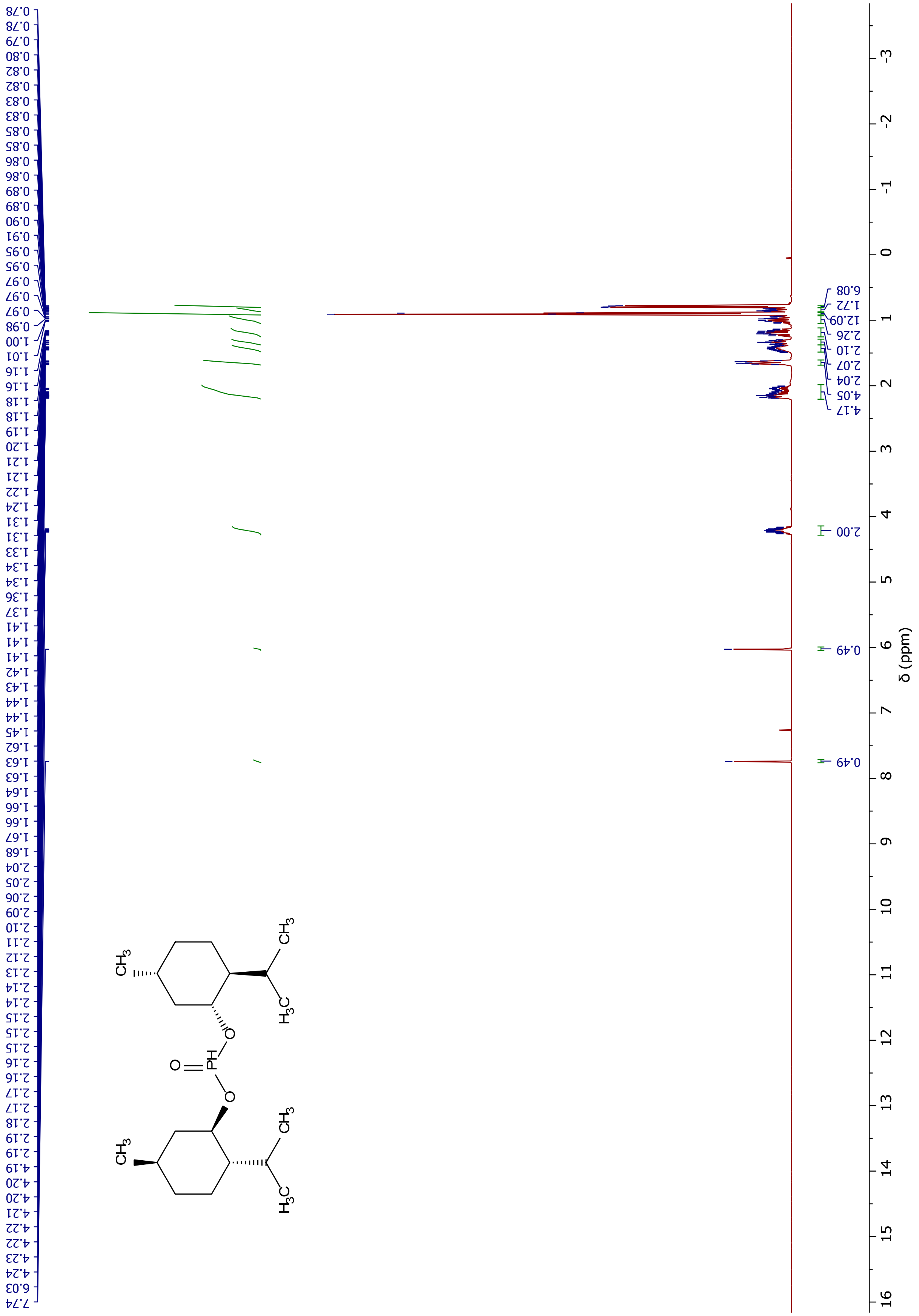

$^{13}C\{^{1}H\}$-NMR spectra of 13

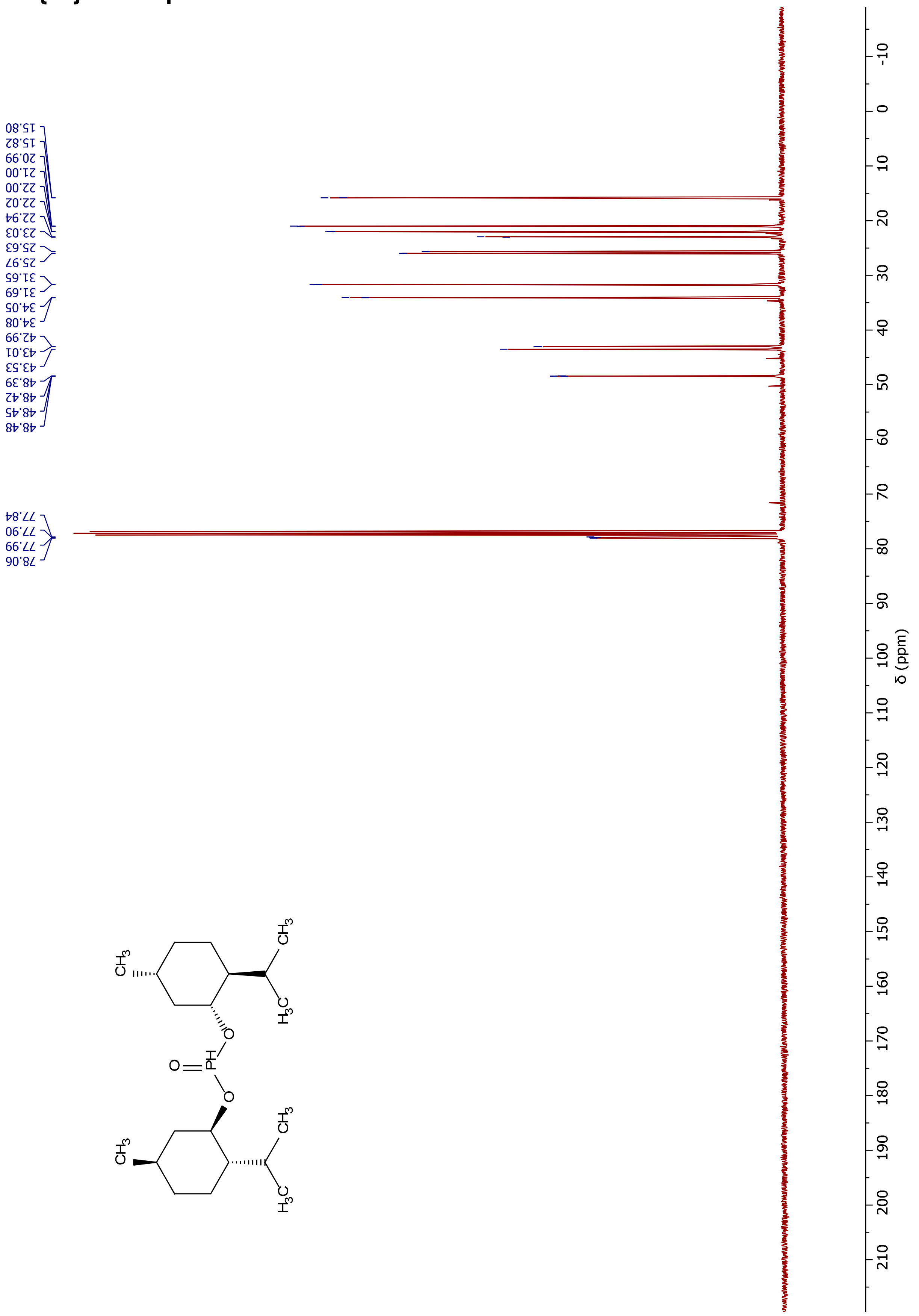

$^{31}P\{^{1}H\}$-NMR spectra of 13

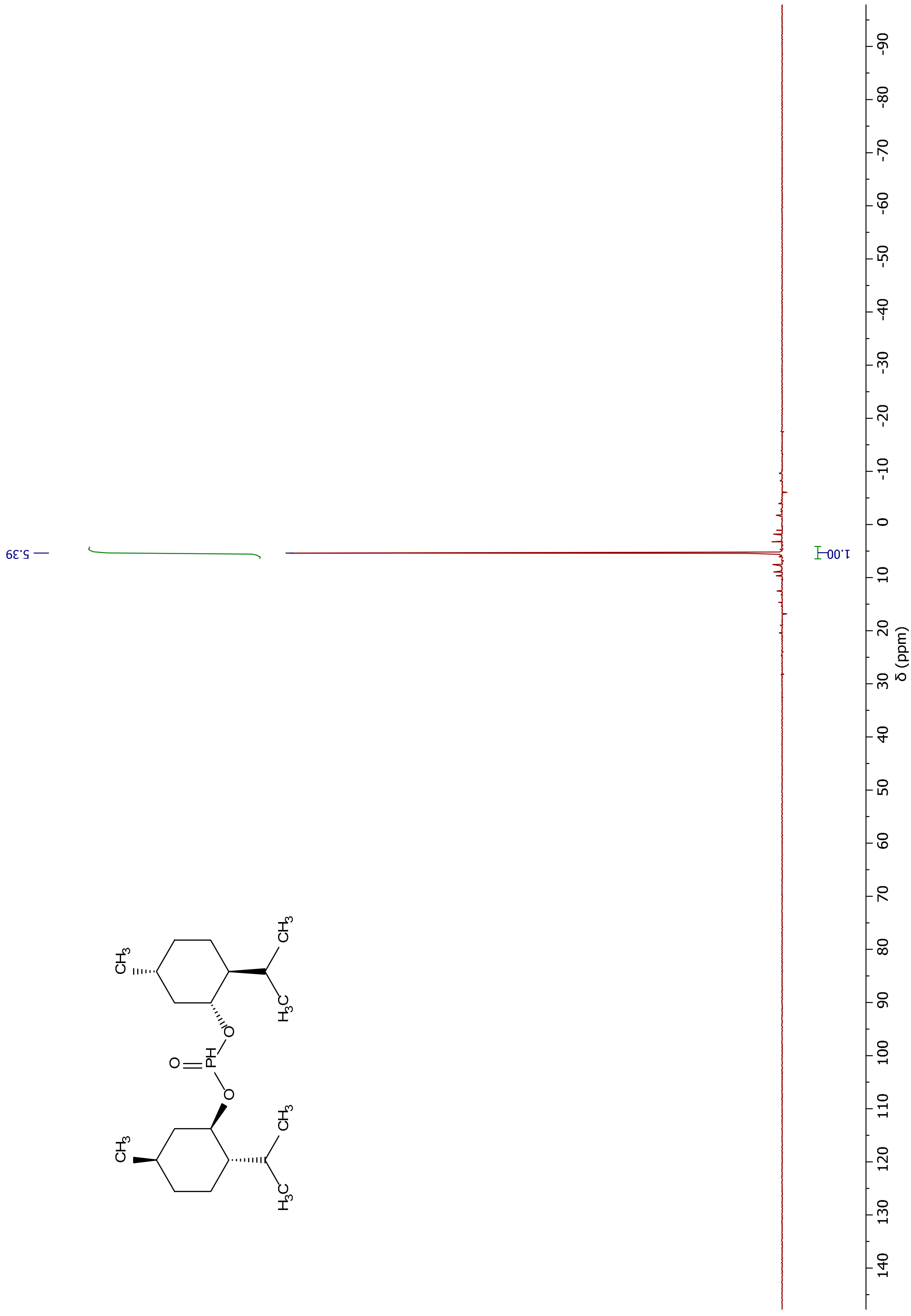

^{1}H-NMR spectra of 14

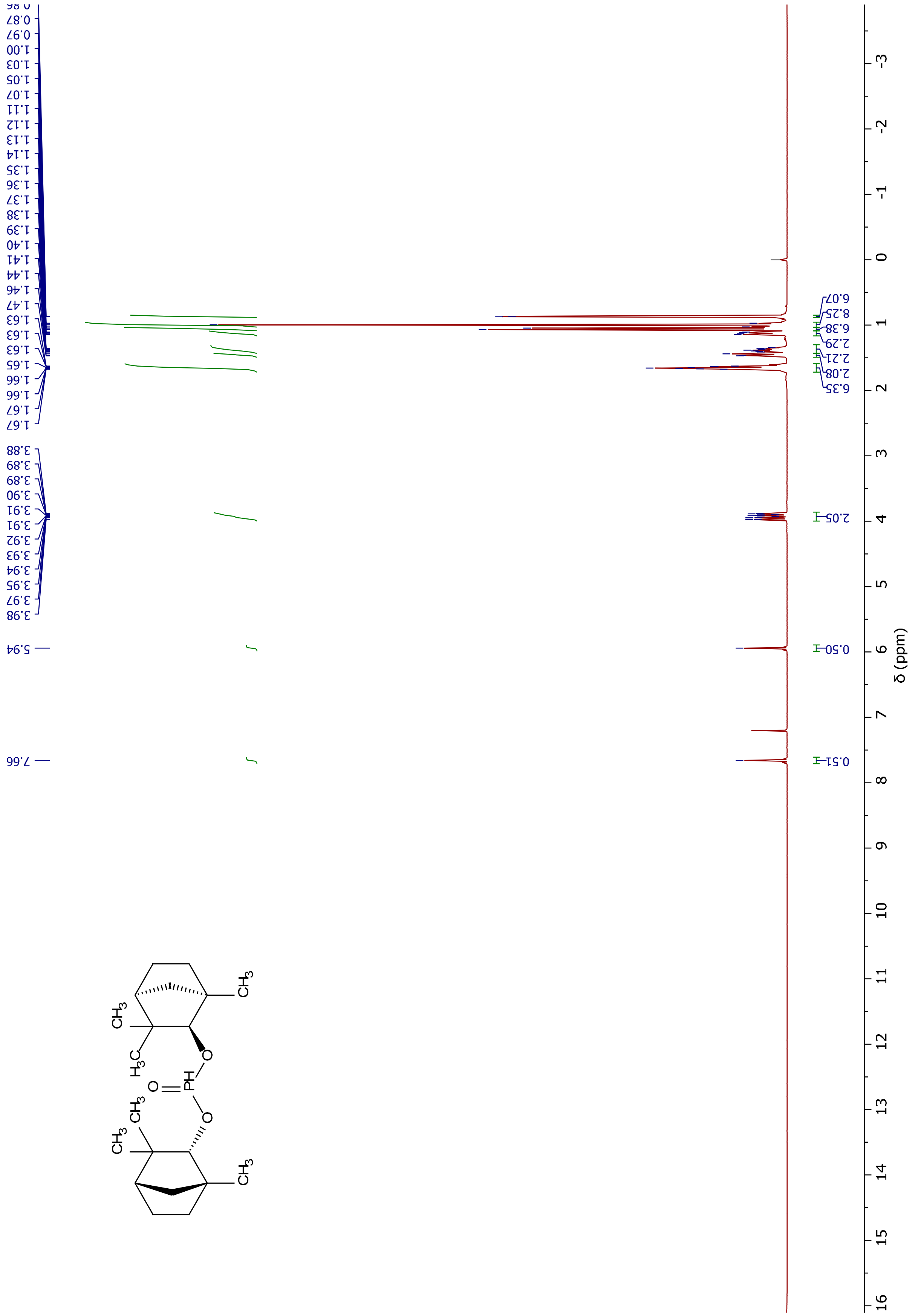

$^{13}C\{^{1}H\}$-NMR spectra of 14

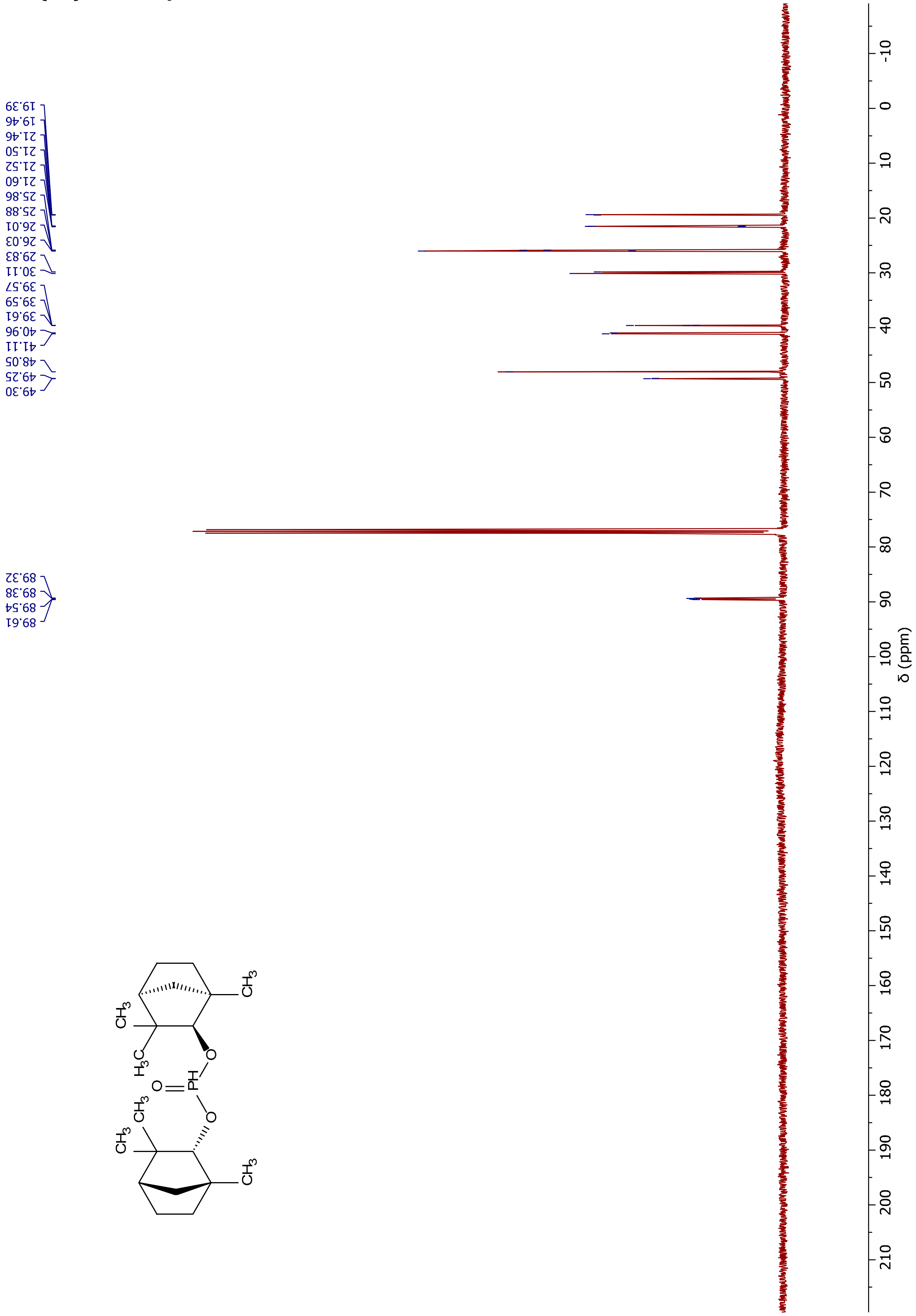

$^{31}P\{^{1}H\}$-NMR spectra of 14

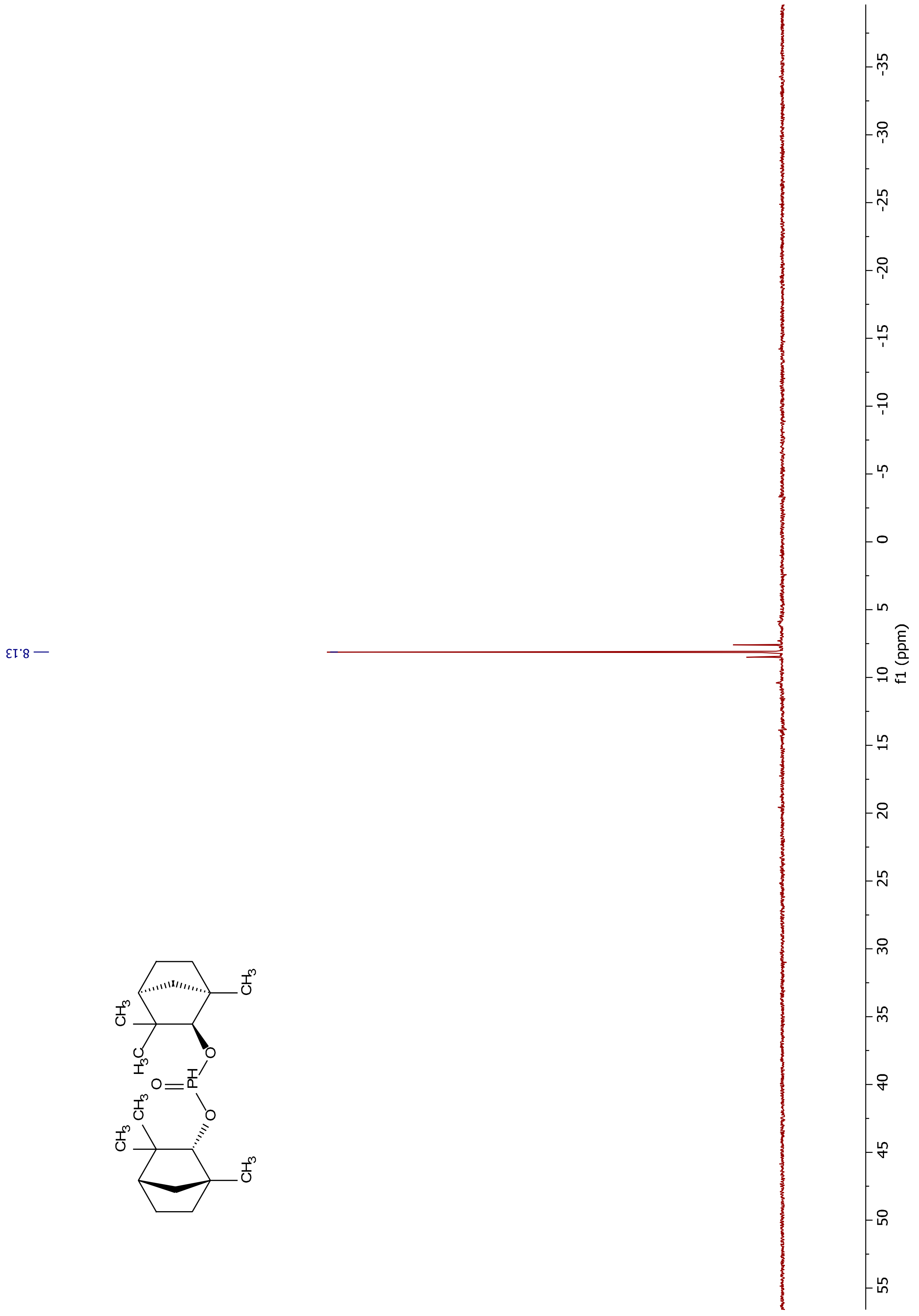

^{1}H-NMR spectra of 15

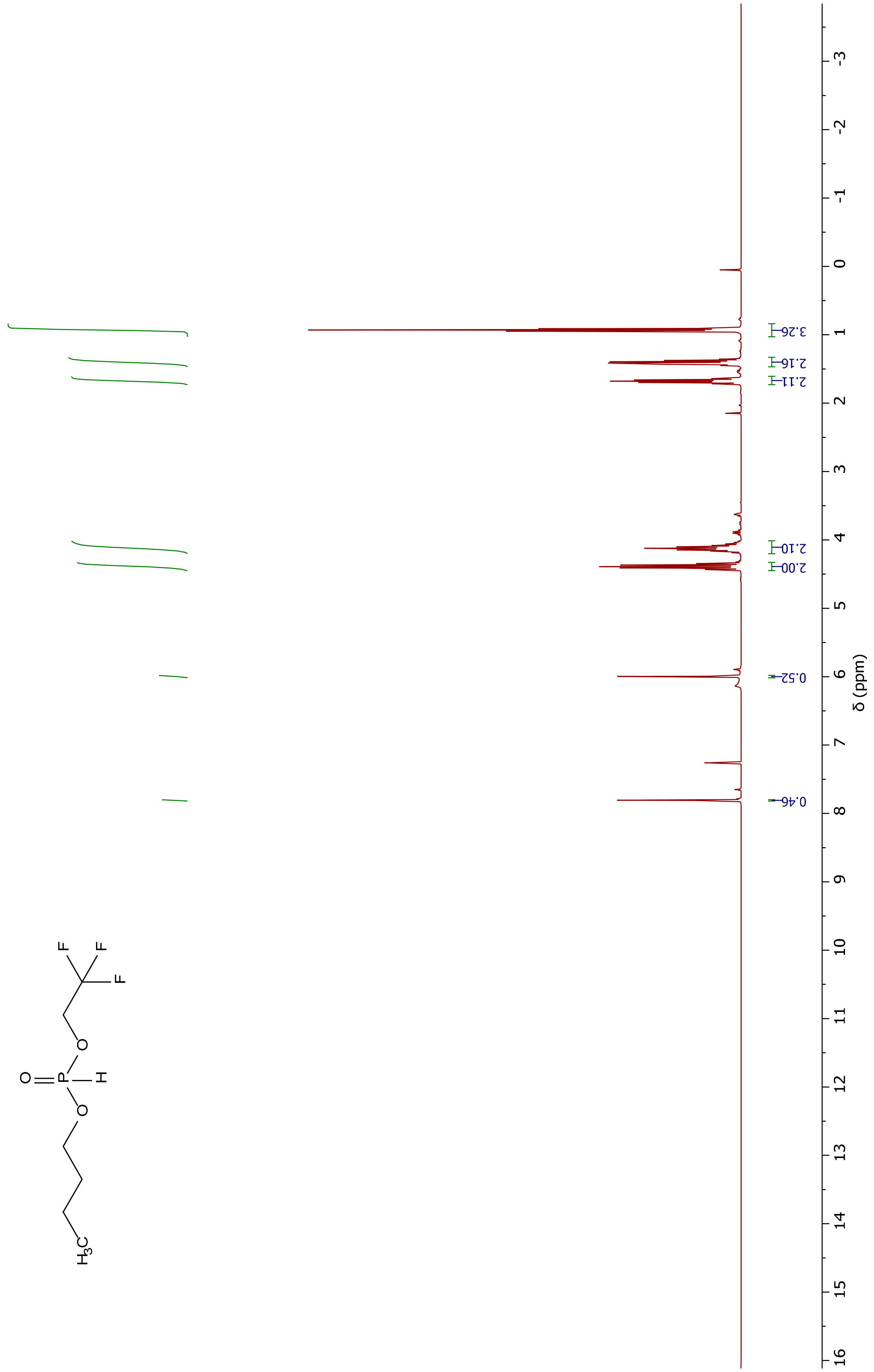

$^{13}C\{^1H\}$-NMR spectra of 15

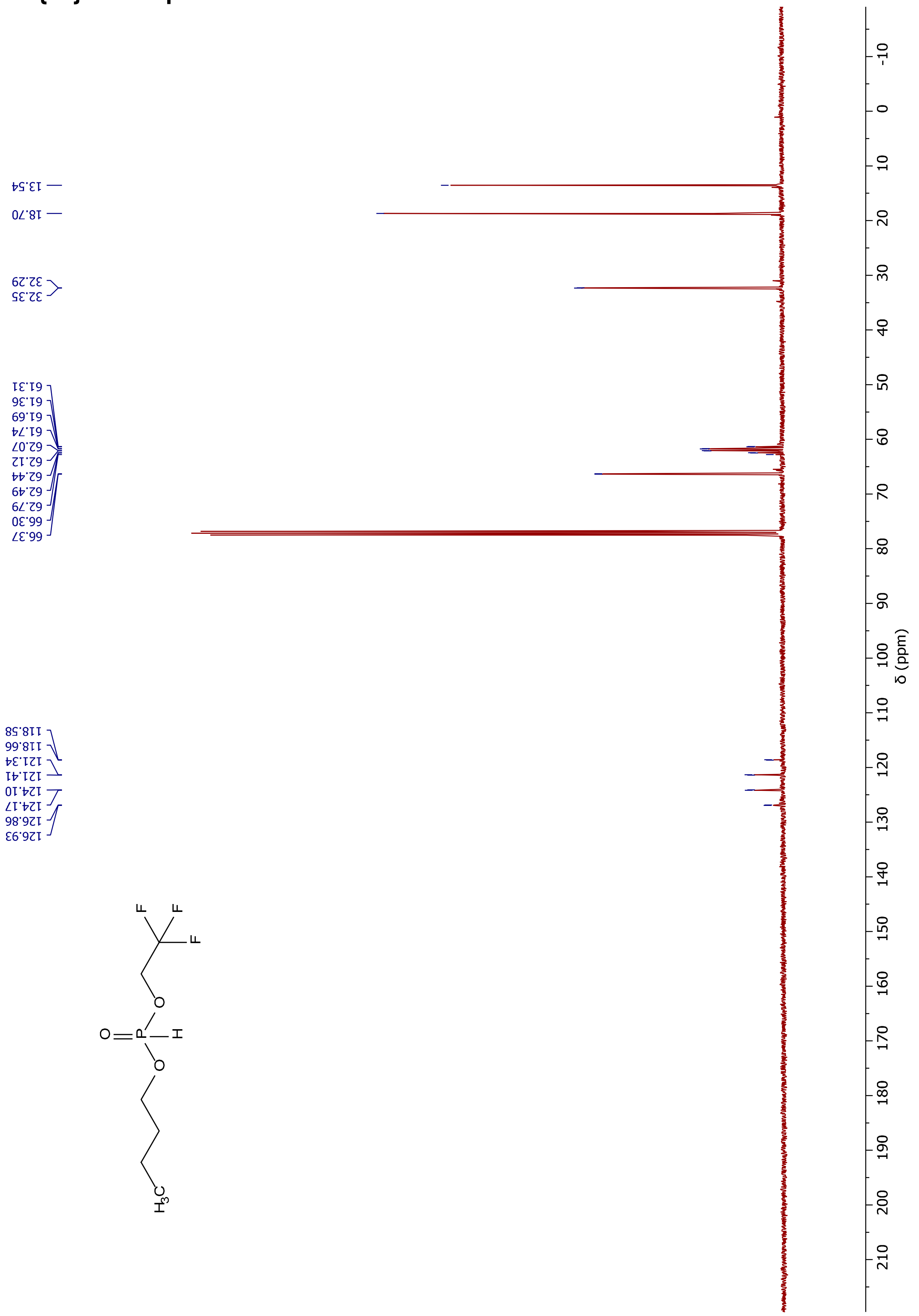

$^{31}P\{^{1}H\}$-NMR spectra of 15

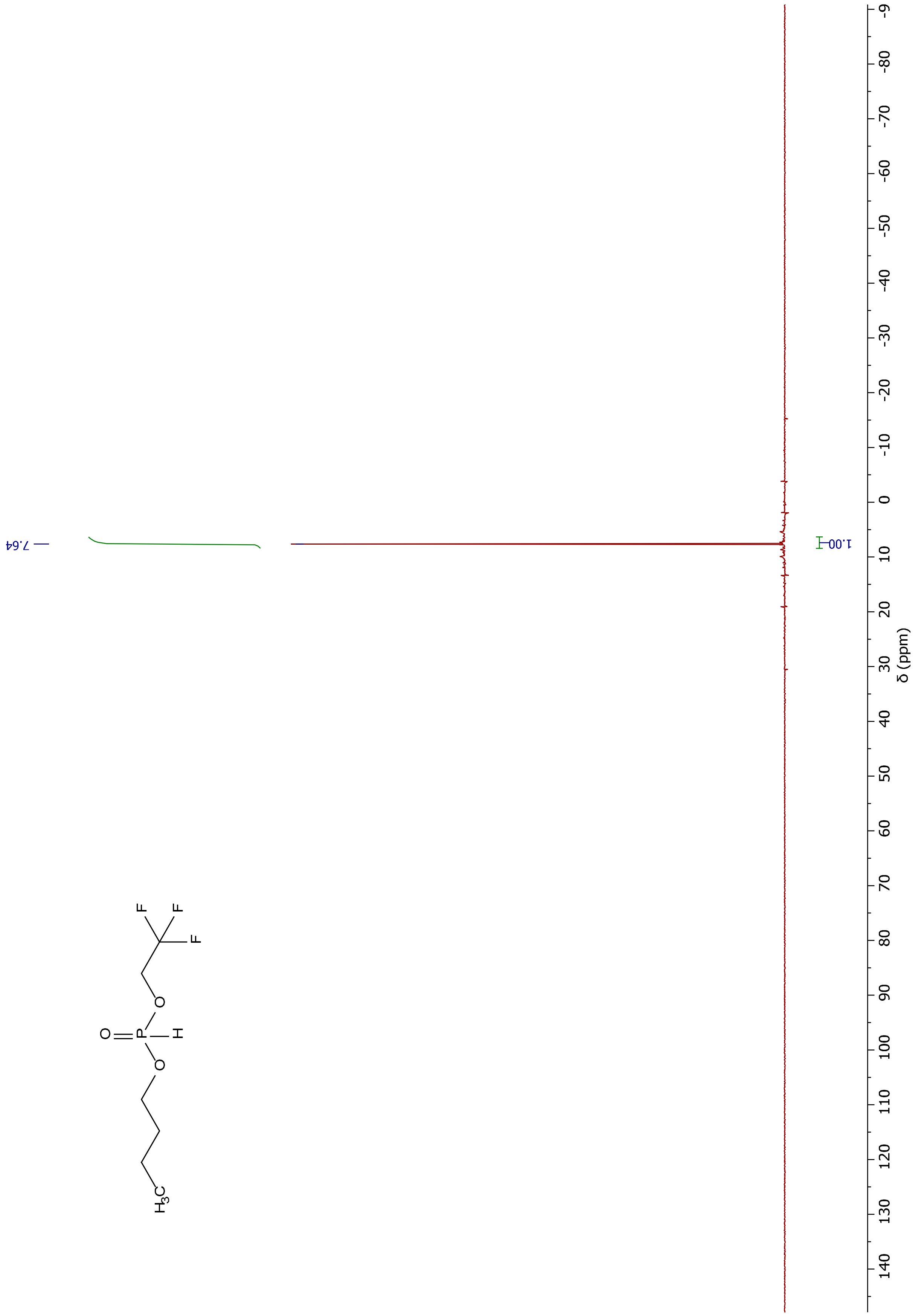

^{1}H-NMR spectra of 16

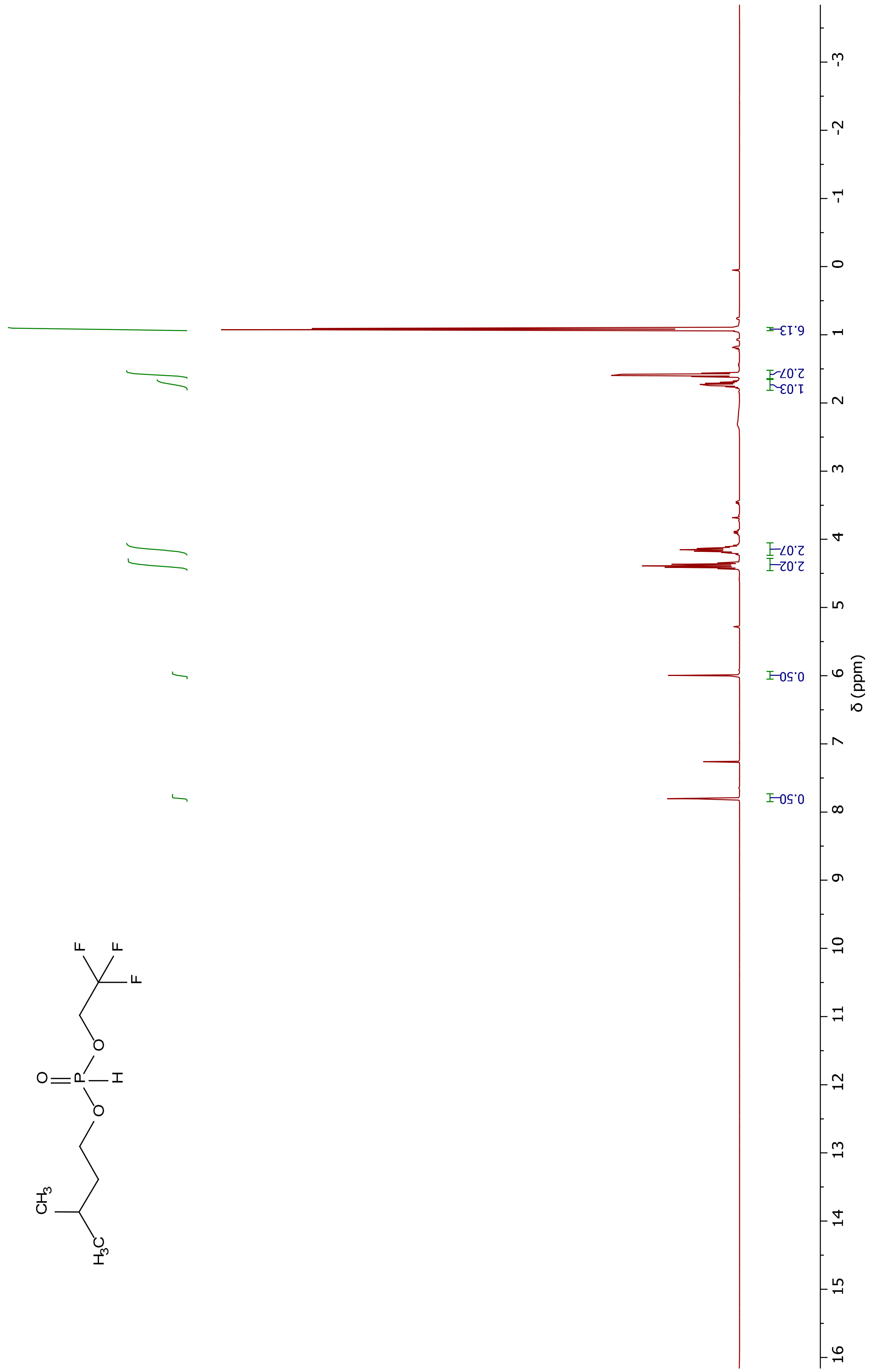

$^{13}C\{^{1}H\}$-NMR spectra of 16

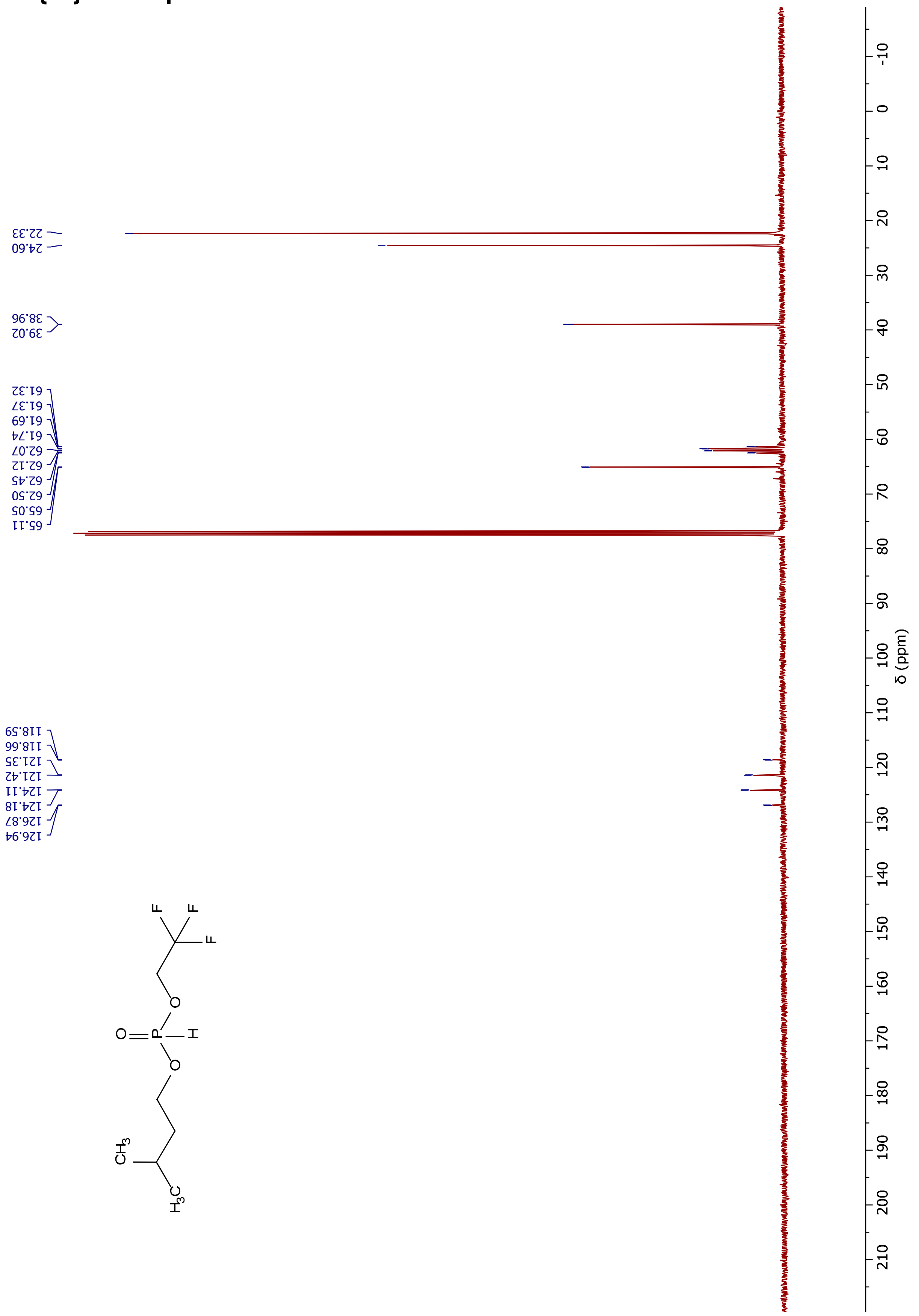

$^{31}P\{^{1}H\}$-NMR spectra of 16

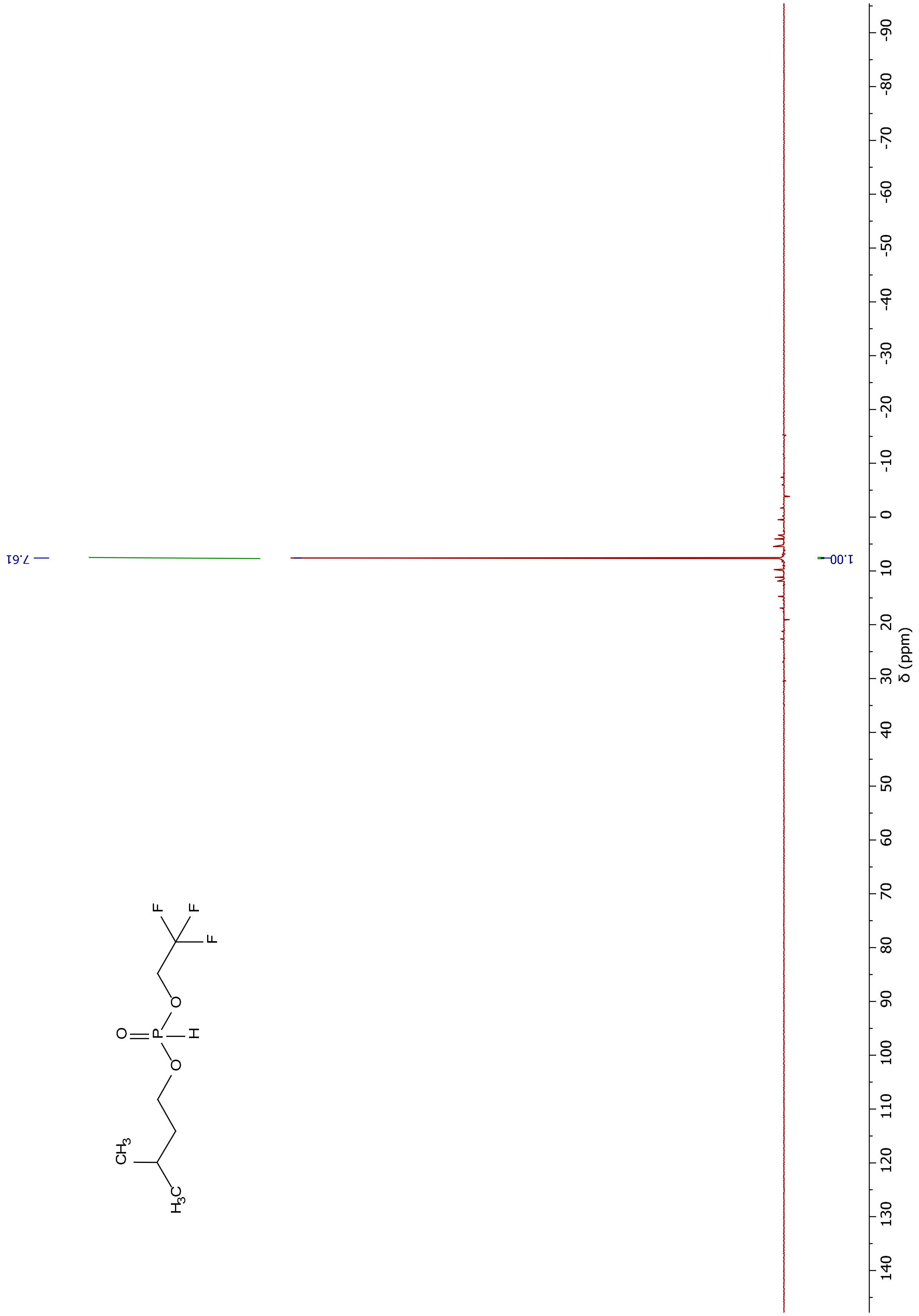

^{1}H-NMR spectra of 17

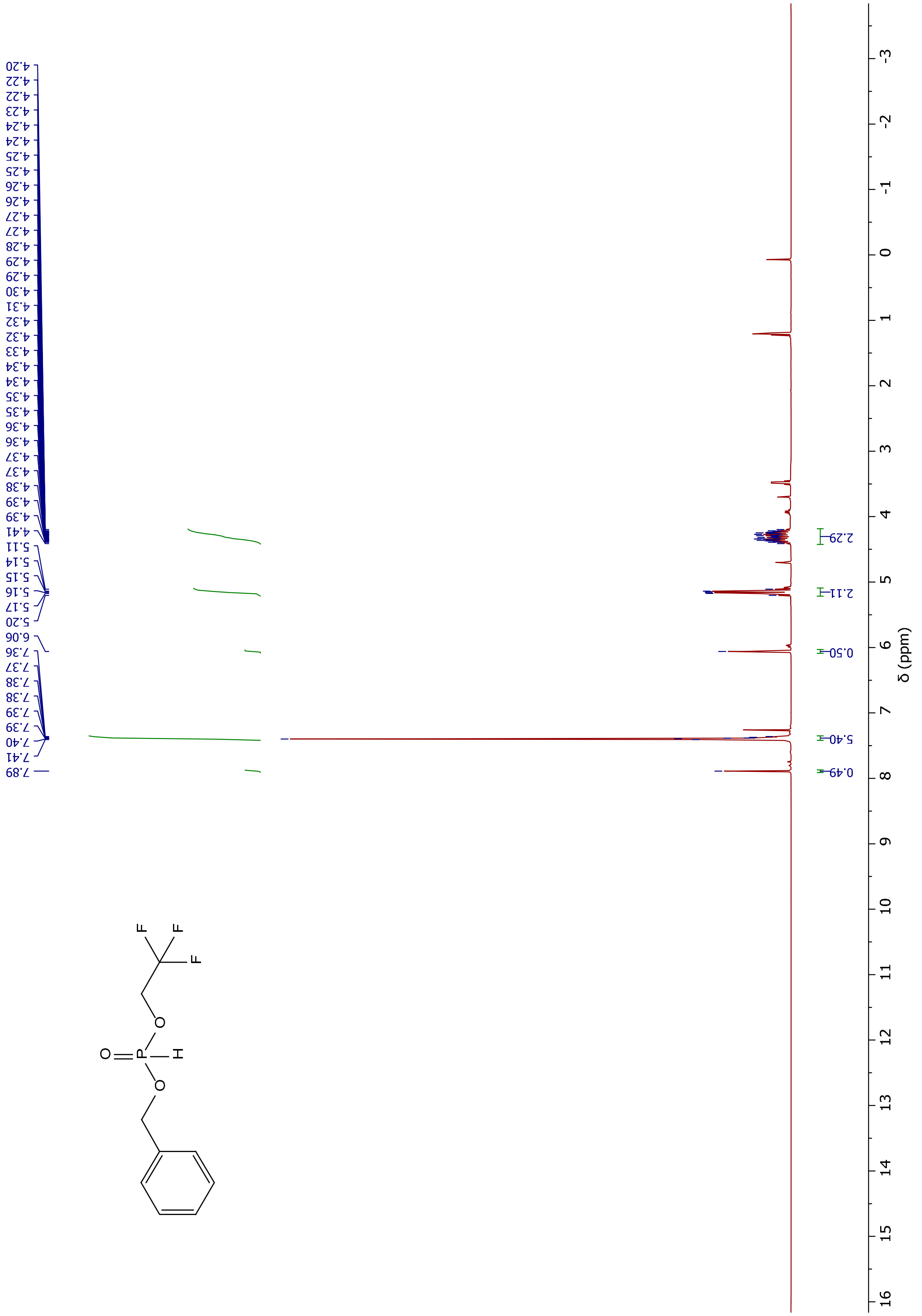

$^{13}C\{^{1}H\}$-NMR spectra of 17

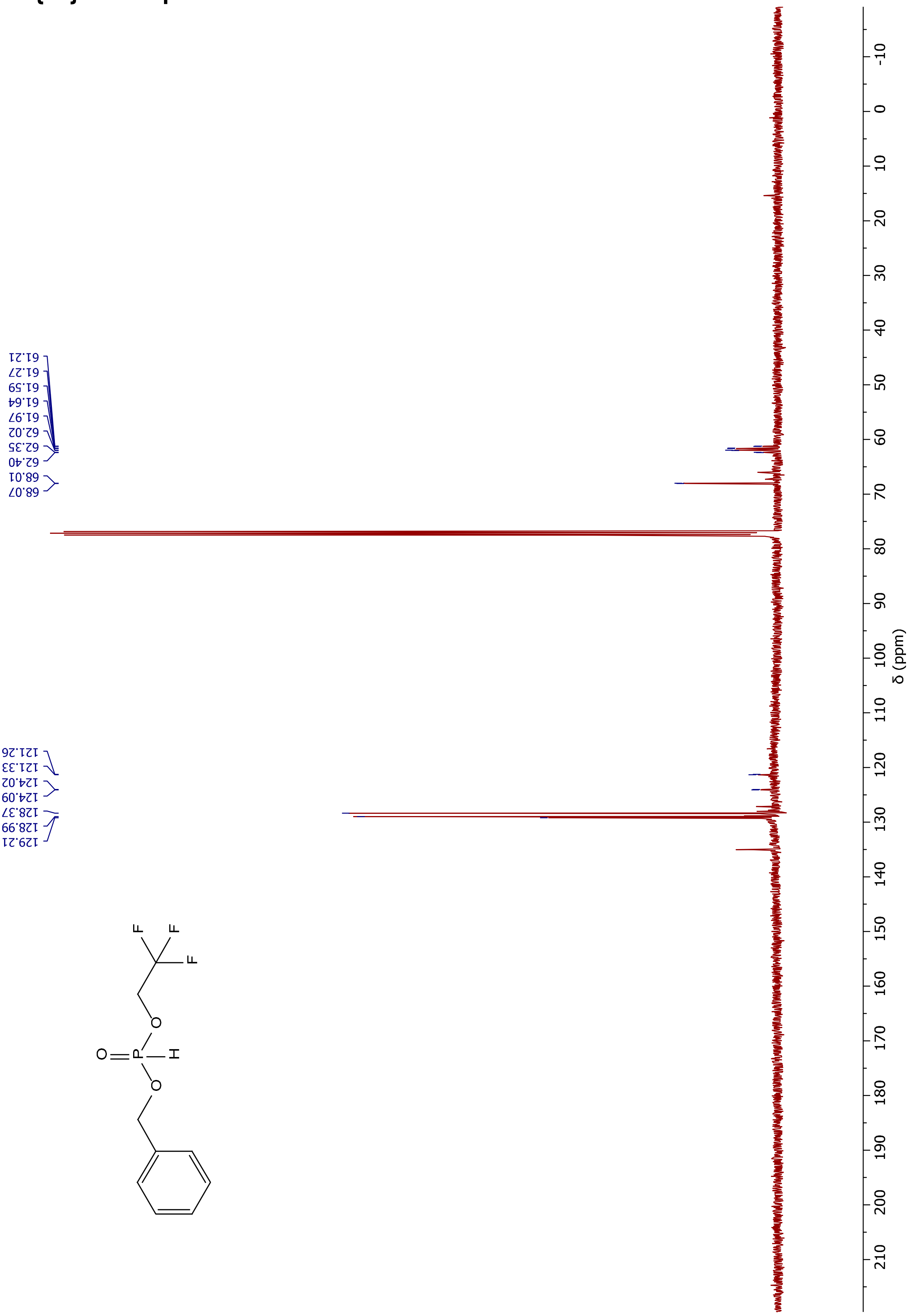

$^{31}P\{^1H\}$-NMR spectra of 17

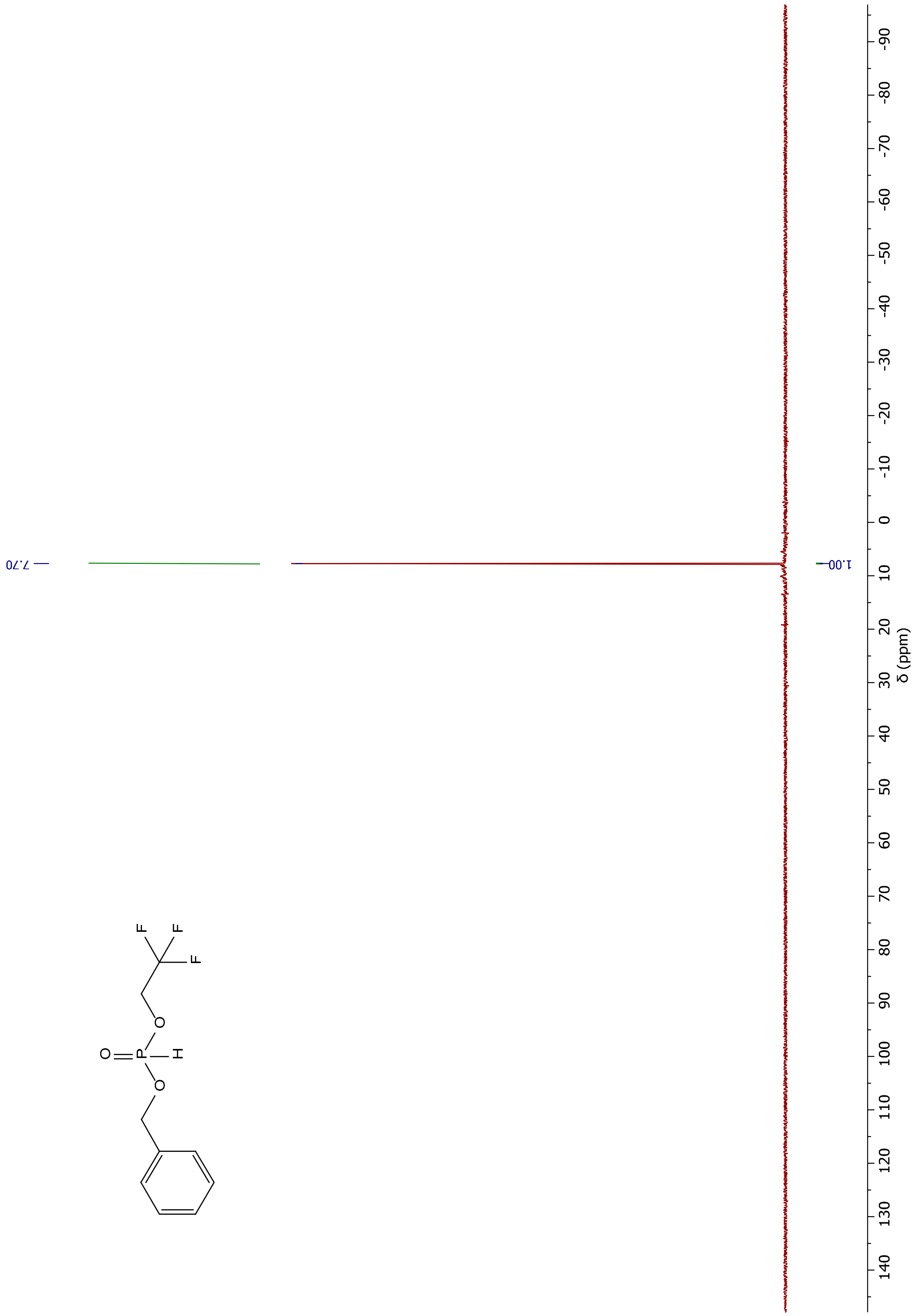

^{1}H-NMR spectra of 18

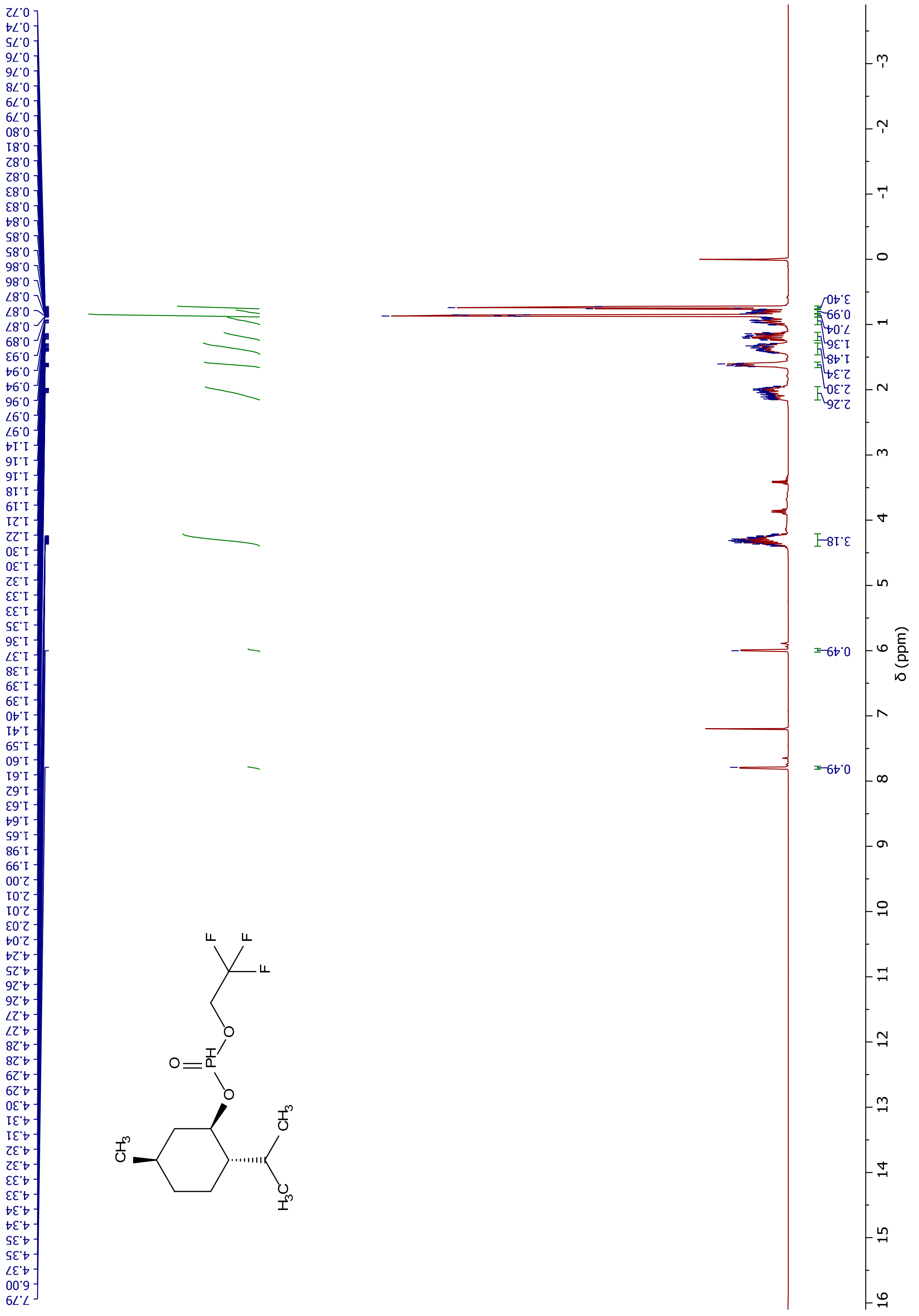

$^{13}C\{^{1}H\}$-NMR spectra of 18

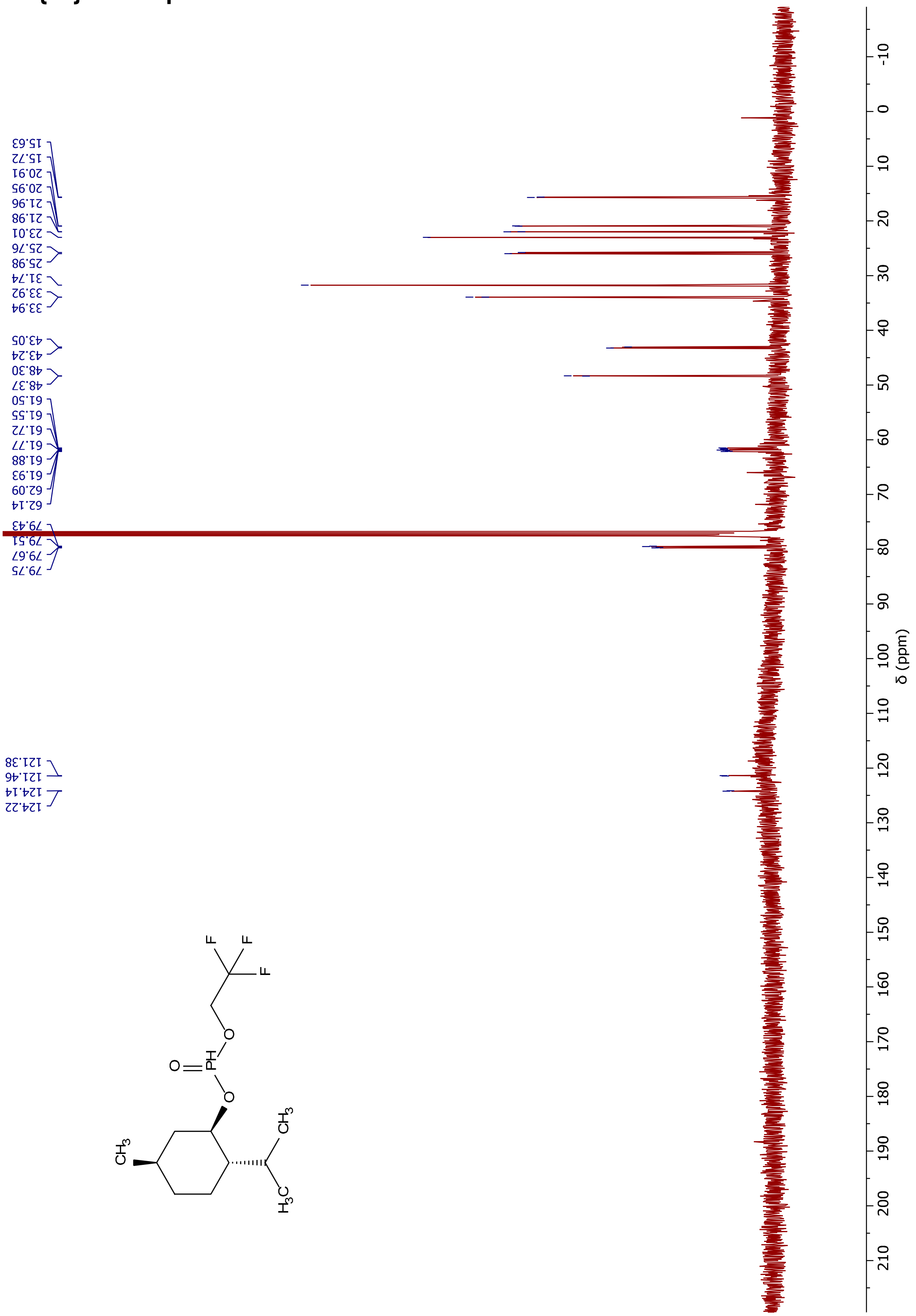

$^{31}P\{^{1}H\}$-NMR spectra of 18

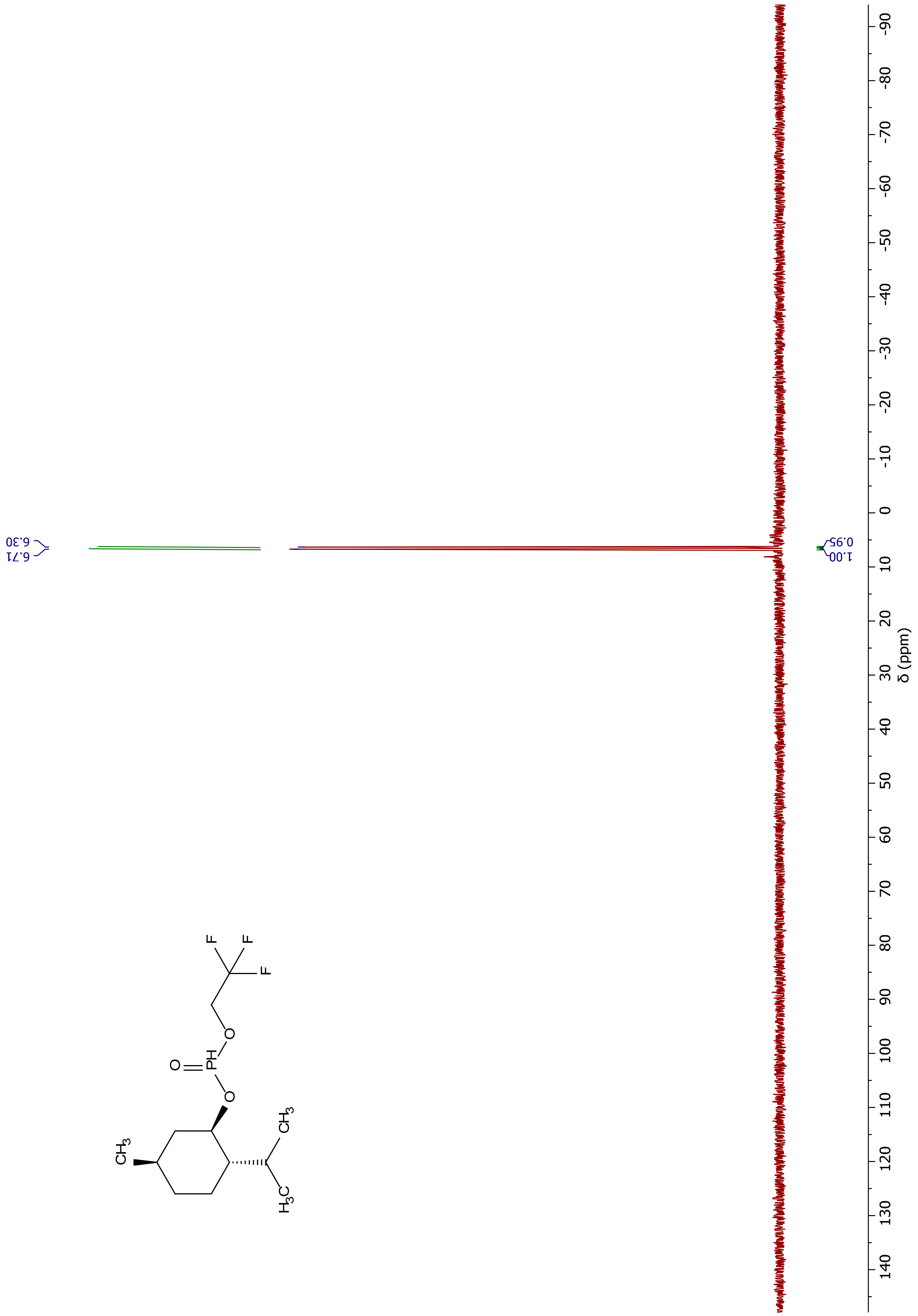

^{1}H-NMR spectra of 19

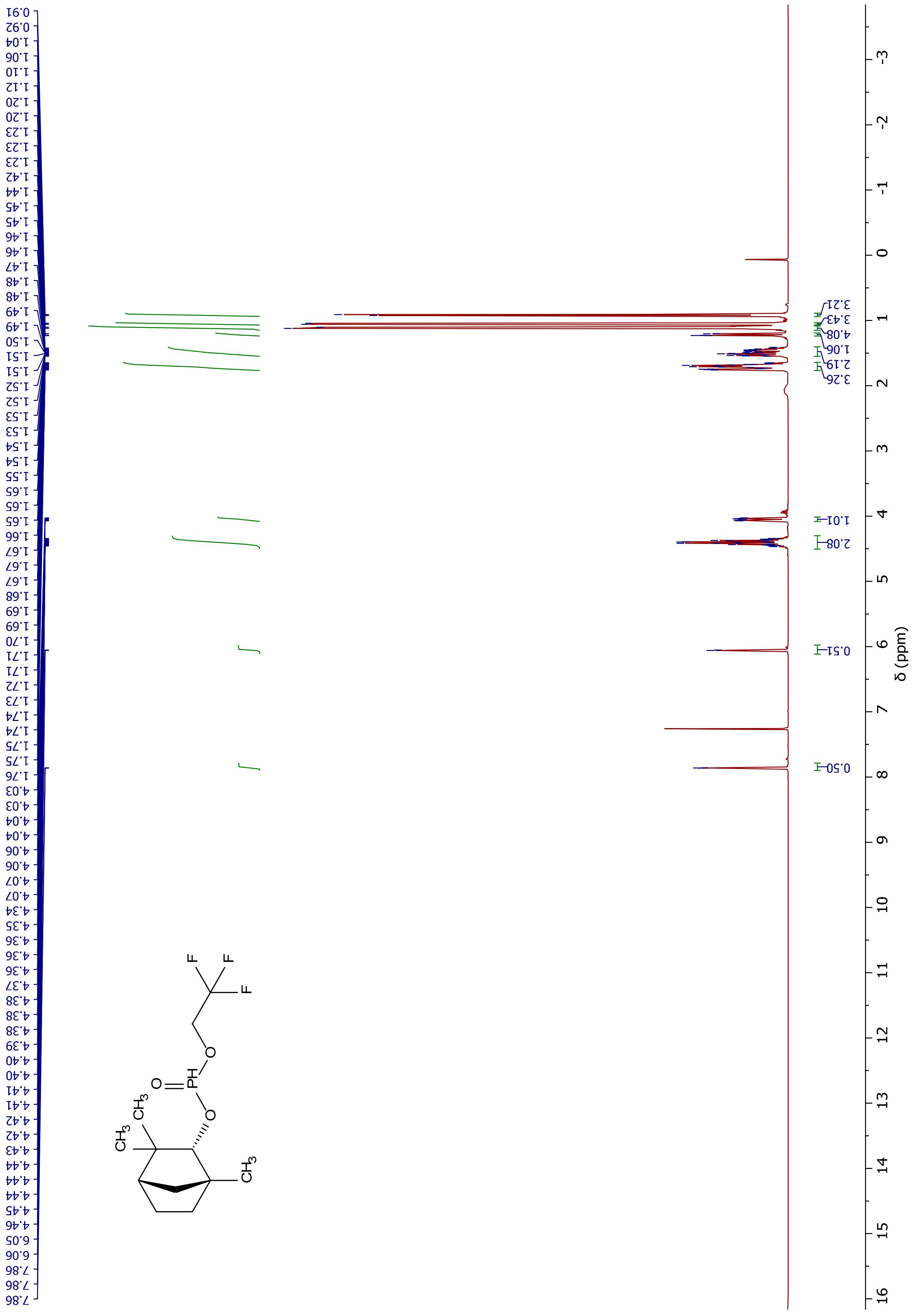

^{13}C{^{1}H}-NMR spectra of 19

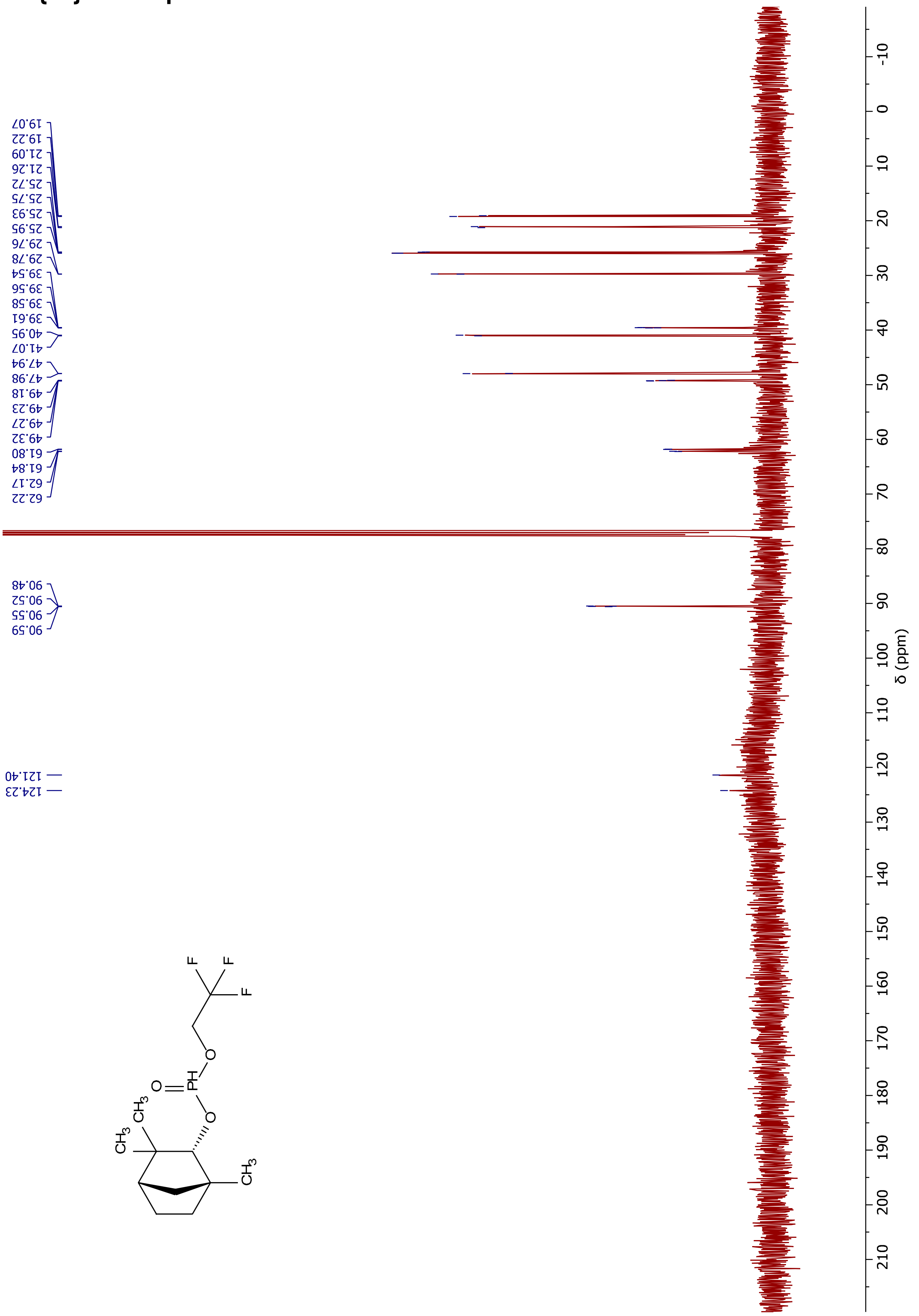

^{31}P{^{1}H}-NMR spectra of 19

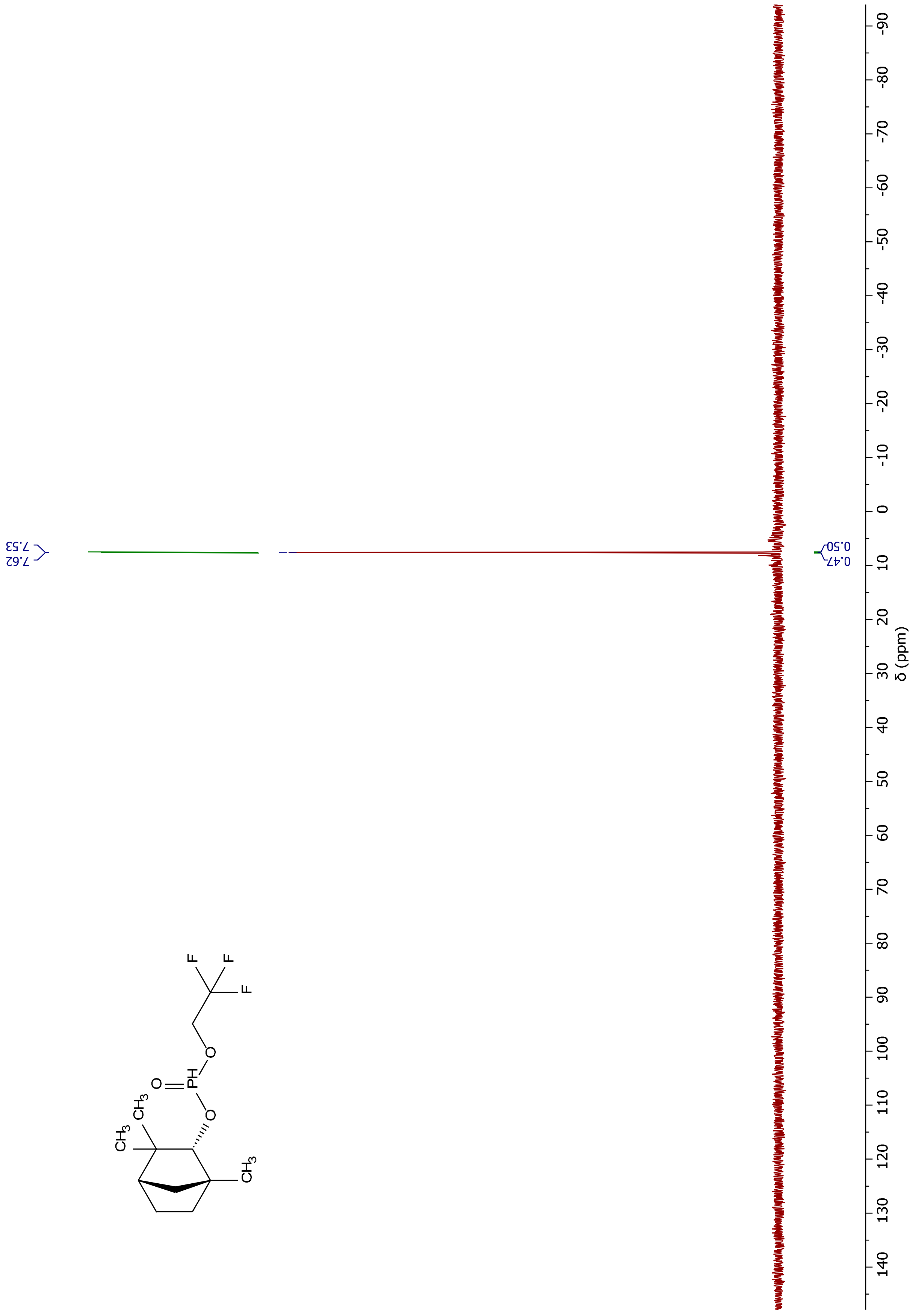

^{31}P-NMR spectra of 19

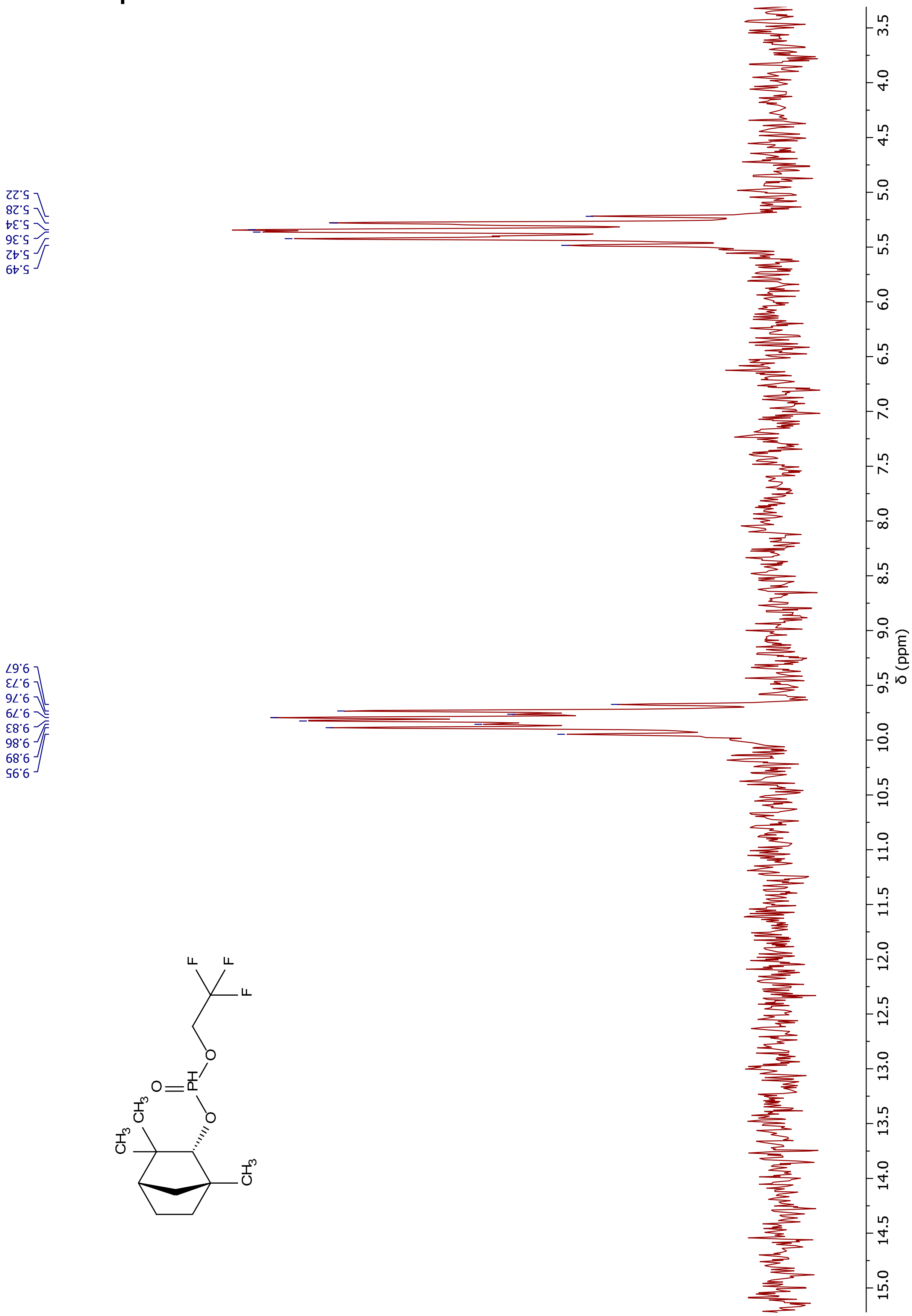

^{1}H-NMR spectra of 20

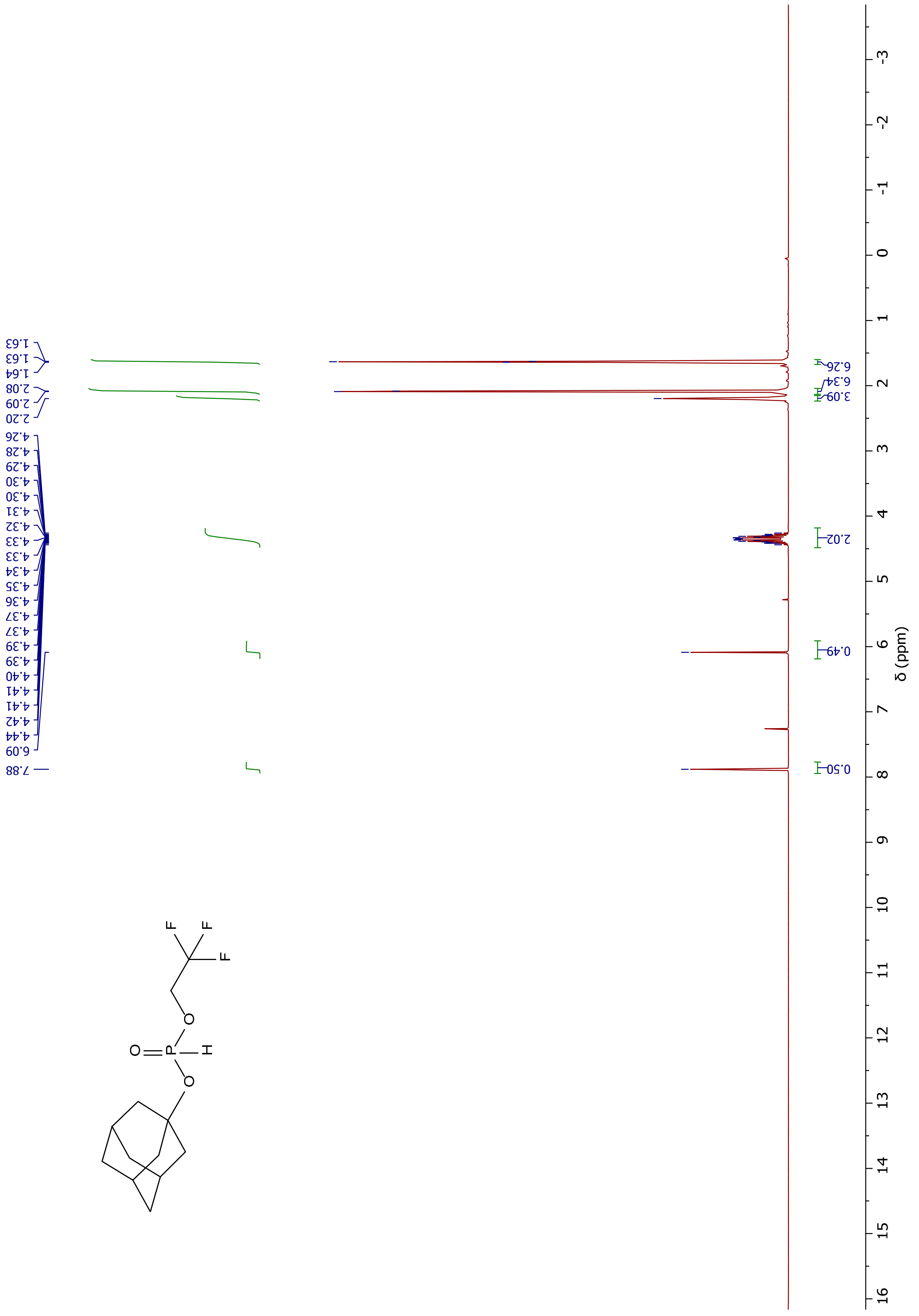

^{13}C{^{1}H}-NMR spectra of 20

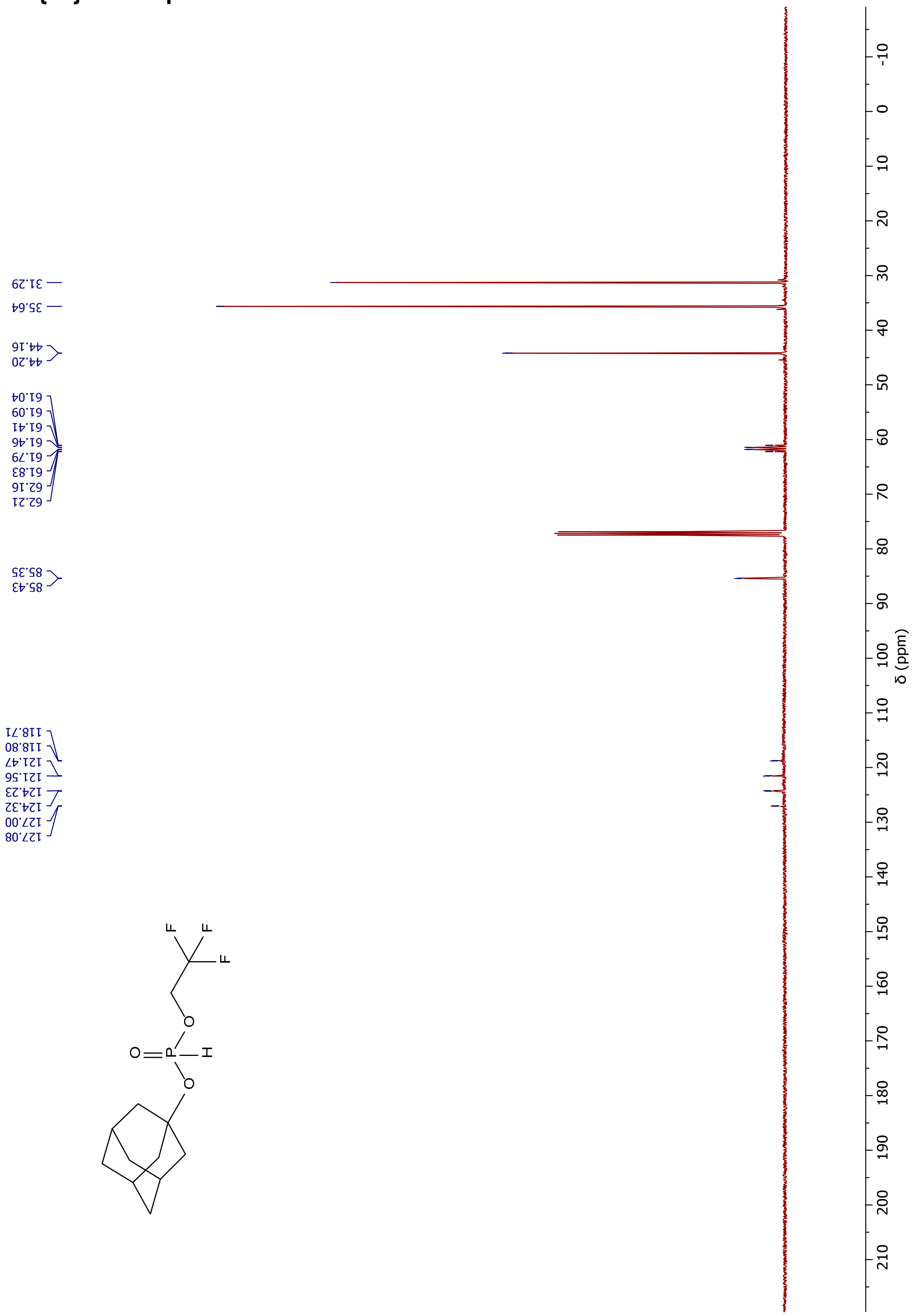

$^{31}P\{^{1}H\}$-NMR spectra of 20

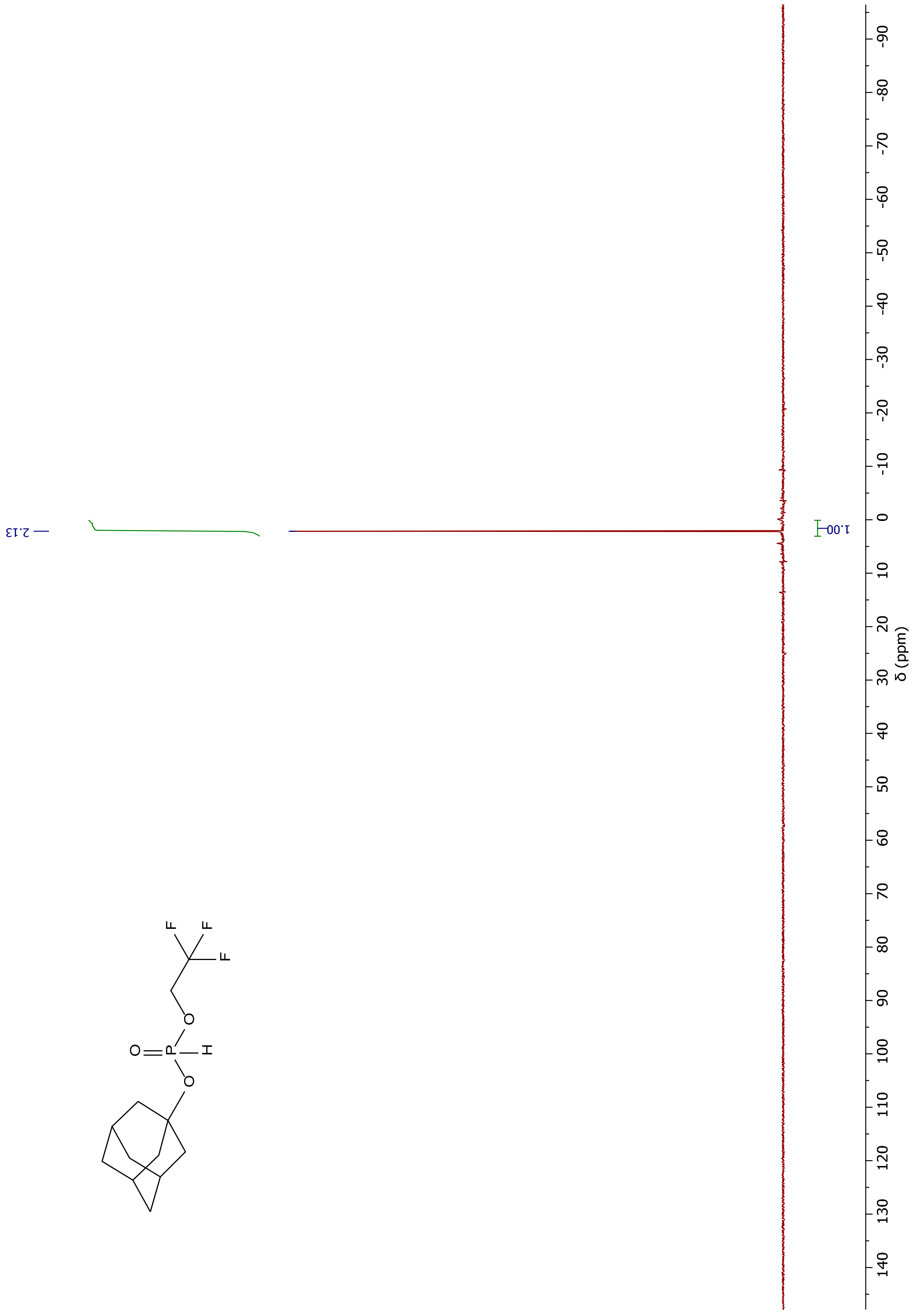

SCXRD Analysis of (4R,5R)-2-Hydro-2-oxo-4,5-diphenyl-1,3,2-dioxaphospholane

Diffraction data for **1** were collected at low temperatures (100K) using ϕ- and ω-scans on a BRUKER D8 Venture system equipped with dual IµS microfocus sources, a PHOTON100 detector and an OXFORD CRYOSYSTEMS 700 low temperature system. Mo-K_{α} radiation with wavelength 0.71073 Å and a collimating Quazar multilayer mirror were used. Semi-empirical absorption correction from equivalents was applied using SADABS-2016/2 [1] and the structure was solved by direct methods using SHELXT2014/5 [2] orthorhombic space group $P2_12_12_1$. Refinement was performed against F^2 on all data by full-matrix least squares using SHELXL2018/3 [3]. All non-hydrogen atoms were refined anisotropically, the P-H hydrogen atom was located in the Fourier, and C-H hydrogen atoms were positioned at geometrically calculated positions and refined using a riding model. The isotropic displacement parameters of all hydrogen atoms were fixed to 1.2x the U_{eq} value of the atoms they are linked to. The asymmetric unit contains one molecule of **1**. Anomalous dispersion was used to determine the absolute structure. While the Parsons parameter of 0.152(71) [4] could also indicate racemic twinning, statistical analysis of Bijvoet pairs [5] as implemented in PLATON [6] confirmed the correct assignment of the absolute structure. The crystallographic data has been deposited with the Cambridge Crystallographic Data Centre as CCDC No. 2036186 and can be obtained free of charge.[https://www.ccdc.cam.ac.uk/structures/]

Table S1. Crystal data and structure refinement for **1**.

CCDC No	2036186	
Empirical formula	$C_{14} H_{13} O_3 P$	
Formula weight	260.21	
Temperature	100(2) K	
Wavelength	0.71073 Å	
Crystal system	Orthorhombic	
Space group	$P2_12_12_1$	
Unit cell dimensions	a = 5.5890(4) Å	α= 90°.
	b = 14.4462(11) Å	β= 90°.
	c = 15.1865(11) Å	γ = 90°.
Volume	1226.16(16) Å^3	
Z	4	
Density (calculated)	1.410 Mg/m^3	
Absorption coefficient	0.221 mm^{-1}	
F(000)	544	
Crystal size	0.330 x 0.028 x 0.020 mm^3	
Theta range for data collection	1.946 to 25.678°.	
Index ranges	-6<=h<=6, -17<=k<=17, -18<=l<=18	
Reflections collected	24647	
Independent reflections	2325 [R(int) = 0.1081]	
Completeness to theta = 25.242°	99.9 %	
Absorption correction	Semi-empirical from equivalents	
Refinement method	Full-matrix least-squares on F^2	
Data / restraints / parameters	2325 / 0 / 166	
Goodness-of-fit on F^2	1.047	
Final R indices [I>2σ(I)]	R1 = 0.0493, wR2 = 0.1130	
R indices (all data)	R1 = 0.0654, wR2 = 0.1215	
Absolute structure parameter	0.15(7)	
Largest diff. peak and hole	0.448 and -0.352 e.Å^{-3}	

Table S2. Atomic coordinates (x 10^4) and equivalent isotropic displacement parameters ($Å^2$x 10^3) for **1**. U(eq) is defined as one third of the trace of the orthogonalized U^{ij} tensor.

	x	y	z	U(eq)
P(1)	1078(2)	3474(1)	4377(1)	23(1)
O(1)	2358(6)	4093(2)	5104(2)	26(1)
O(2)	1866(6)	4050(2)	3528(2)	23(1)
O(3)	-1488(6)	3332(2)	4488(2)	30(1)
C(1)	3832(9)	4811(3)	4712(3)	22(1)
C(2)	2735(9)	4968(3)	3792(3)	19(1)
C(3)	3849(9)	5655(3)	5279(3)	21(1)
C(4)	5720(8)	6287(3)	5225(3)	22(1)
C(5)	5726(9)	7084(3)	5721(3)	26(1)
C(6)	3860(9)	7264(3)	6294(3)	27(1)
C(7)	1982(8)	6637(3)	6363(3)	23(1)
C(8)	1945(9)	5835(4)	5863(3)	22(1)
C(9)	4427(8)	5327(3)	3100(3)	18(1)
C(10)	3891(9)	6137(3)	2655(3)	22(1)
C(11)	5432(9)	6468(4)	1995(3)	28(1)
C(12)	7471(9)	5976(4)	1790(3)	25(1)
C(13)	7992(9)	5168(4)	2239(3)	23(1)
C(14)	6471(9)	4837(3)	2889(3)	21(1)

Table S3. Bond lengths [Å] and angles [°] for **1**.

P(1)-O(3)	1.458(3)	O(3)-P(1)-H(1)	111(2)
P(1)-O(1)	1.591(4)	O(1)-P(1)-H(1)	111(2)
P(1)-O(2)	1.596(3)	O(2)-P(1)-H(1)	103(2)
P(1)-H(1)	1.21(5)	C(1)-O(1)-P(1)	111.9(3)
O(1)-C(1)	1.452(6)	C(2)-O(2)-P(1)	110.0(3)
O(2)-C(2)	1.469(6)	O(1)-C(1)-C(3)	110.5(4)
C(1)-C(3)	1.492(6)	O(1)-C(1)-C(2)	104.5(4)
C(1)-C(2)	1.542(6)	C(3)-C(1)-C(2)	113.9(4)
C(1)-H(1A)	1.0000	O(1)-C(1)-H(1A)	109.2
C(2)-C(9)	1.506(6)	C(3)-C(1)-H(1A)	109.2
C(2)-H(2)	1.0000	C(2)-C(1)-H(1A)	109.2
C(3)-C(4)	1.391(7)	O(2)-C(2)-C(9)	109.2(4)
C(3)-C(8)	1.409(7)	O(2)-C(2)-C(1)	104.2(3)
C(4)-C(5)	1.377(7)	C(9)-C(2)-C(1)	115.7(4)
C(4)-H(4)	0.9500	O(2)-C(2)-H(2)	109.2
C(5)-C(6)	1.383(7)	C(9)-C(2)-H(2)	109.2
C(5)-H(5)	0.9500	C(1)-C(2)-H(2)	109.2
C(6)-C(7)	1.391(7)	C(4)-C(3)-C(8)	118.9(5)
C(6)-H(6)	0.9500	C(4)-C(3)-C(1)	120.4(4)
C(7)-C(8)	1.386(7)	C(8)-C(3)-C(1)	120.6(4)
C(7)-H(7)	0.9500	C(5)-C(4)-C(3)	121.2(5)
C(8)-H(8)	0.9500	C(5)-C(4)-H(4)	119.4
C(9)-C(14)	1.382(7)	C(3)-C(4)-H(4)	119.4
C(9)-C(10)	1.384(7)	C(4)-C(5)-C(6)	120.0(5)
C(10)-C(11)	1.405(7)	C(4)-C(5)-H(5)	120.0
C(10)-H(10)	0.9500	C(6)-C(5)-H(5)	120.0
C(11)-C(12)	1.379(7)	C(5)-C(6)-C(7)	119.6(5)
C(11)-H(11)	0.9500	C(5)-C(6)-H(6)	120.2
C(12)-C(13)	1.382(7)	C(7)-C(6)-H(6)	120.2
C(12)-H(12)	0.9500	C(8)-C(7)-C(6)	121.0(5)
C(13)-C(14)	1.388(7)	C(8)-C(7)-H(7)	119.5
C(13)-H(13)	0.9500	C(6)-C(7)-H(7)	119.5
C(14)-H(14)	0.9500	C(7)-C(8)-C(3)	119.2(5)
		C(7)-C(8)-H(8)	120.4
O(3)-P(1)-O(1)	116.1(2)	C(3)-C(8)-H(8)	120.4
O(3)-P(1)-O(2)	116.0(2)	C(14)-C(9)-C(10)	119.9(4)
O(1)-P(1)-O(2)	98.24(17)	C(14)-C(9)-C(2)	120.3(4)

C(10)-C(9)-C(2)	119.8(4)
C(9)-C(10)-C(11)	120.2(5)
C(9)-C(10)-H(10)	119.9
C(11)-C(10)-H(10)	119.9
C(12)-C(11)-C(10)	119.5(5)
C(12)-C(11)-H(11)	120.3
C(10)-C(11)-H(11)	120.3
C(11)-C(12)-C(13)	119.9(5)
C(11)-C(12)-H(12)	120.1
C(13)-C(12)-H(12)	120.1
C(12)-C(13)-C(14)	120.9(5)
C(12)-C(13)-H(13)	119.6
C(14)-C(13)-H(13)	119.6
C(9)-C(14)-C(13)	119.7(4)
C(9)-C(14)-H(14)	120.2
C(13)-C(14)-H(14)	120.2

Table S4. Anisotropic displacement parameters ($Å^2 x 10^3$) for **1**. The anisotropic displacement factor exponent takes the form: $-2p^2[h^2 a^{*2}U^{11} + ... + 2 h k a^* b^* U^{12}]$

	U^{11}	U^{22}	U^{33}	U^{23}	U^{13}	U^{12}
P(1)	20(1)	21(1)	28(1)	4(1)	1(1)	1(1)
O(1)	33(2)	21(2)	23(2)	5(1)	3(2)	-5(2)
O(2)	18(2)	24(2)	26(2)	3(2)	1(2)	-3(2)
O(3)	16(2)	24(2)	48(2)	9(2)	0(2)	-5(1)
C(1)	20(2)	22(2)	24(2)	6(2)	3(2)	0(2)
C(2)	18(2)	20(2)	20(2)	4(2)	1(2)	1(2)
C(3)	18(2)	29(2)	16(2)	1(2)	-2(2)	1(2)
C(4)	10(2)	31(3)	25(2)	7(2)	4(2)	0(2)
C(5)	24(3)	28(3)	27(3)	0(2)	0(2)	-6(2)
C(6)	23(3)	29(3)	28(3)	-1(2)	-5(2)	-3(3)
C(7)	17(2)	29(3)	24(2)	3(2)	-1(2)	1(2)
C(8)	14(2)	29(3)	24(3)	3(2)	3(2)	-1(2)
C(9)	17(3)	22(2)	16(2)	-1(2)	-1(2)	-4(2)
C(10)	16(2)	25(2)	25(2)	-4(2)	4(2)	1(2)
C(11)	30(3)	20(2)	32(3)	5(2)	4(2)	-4(2)
C(12)	19(3)	29(3)	27(3)	-2(2)	9(2)	-5(2)
C(13)	17(2)	30(3)	21(2)	0(2)	1(2)	1(2)
C(14)	20(2)	25(2)	19(2)	-1(2)	-4(2)	0(2)

Table S5. Hydrogen coordinates (x 10^4) and isotropic displacement parameters ($Å^2$x 10^3) for **1**.

	x	y	z	U(eq)
H(1)	2110(90)	2750(30)	4280(30)	28
H(1A)	5505	4576	4645	26
H(2)	1345	5400	3845	23
H(4)	7019	6166	4838	26
H(5)	7013	7511	5670	31
H(6)	3860	7814	6638	32
H(7)	706	6760	6760	28
H(8)	652	5411	5914	27
H(10)	2477	6470	2795	26
H(11)	5072	7026	1692	33
H(12)	8515	6191	1341	30
H(13)	9409	4836	2101	27
H(14)	6834	4276	3187	26

Table S6. Hydrogen bonds for **1** [Å and °].

D-H...A	d(D-H)	d(H...A)	d(D...A)	<(DHA)
P(1)-H(1)...O(3)#1	1.21(5)	2.56(5)	3.410(3)	126(3)

Symmetry transformations used to generate equivalent atoms:

#1 x+1/2,-y+1/2,-z+1

References

1. Krause, L.; Herbst-Irmer, R.; Sheldrick, G.M.; Stalke, D. Comparison of silver and molybdenum microfocus X-ray sources for single-crystal structure determination. *Journal of Applied Crystallography* **2015**, *48*, 3–10, doi:10.1107/S1600576714022985.
2. Sheldrick, G.M. \it SHELXT - Integrated space-group and crystal-structure determination. *Acta Crystallographica Section A* **2015**, *71*, 3–8, doi:10.1107/S2053273314026370.
3. Sheldrick, G.M. Crystal structure refinement with \it SHELXL. *Acta Crystallographica Section C* **2015**, *71*, 3–8, doi:10.1107/S2053229614024218.
4. Parsons, S.; Flack, H.D.; Wagner, T. Use of intensity quotients and differences in absolute structure refinement. *Acta Crystallographica Section B* **2013**, *69*, 249–259, doi:10.1107/S2052519213010014.
5. Hooft, R.W.W.; Straver, L.H.; Spek, A.L. Using the \it t-distribution to improve the absolute structure assignment with likelihood calculations. *Journal of Applied Crystallography* **2010**, *43*, 665–668, doi:10.1107/S0021889810018601.
6. Spek, A.L. Single-crystal structure validation with the program \it PLATON. *Journal of Applied Crystallography* **2003**, *36*, 7–13, doi:10.1107/S0021889802022112.
